# Supplementary material for: Effects of acute lying and sleep deprivation on the behavior of lactating dairy cows
Source: PLoS One. 2019 Aug 28;14(8):e0212823. doi: 10.1371/journal.pone.0212823 (PMC6713338; doi:10.1371/journal.pone.0212823)
Supplement: S1 File — Supplemental data to support conclusions drawn on the effects of treatment on the number of steps cows took per day. (DOCX) [file pone.0212823.s003.docx]

**Steps**

| **Type III Tests of Fixed Effects** | | | | |
| --- | --- | --- | --- | --- |
| **Effect** | **Num DF** | **Den DF** | **F Value** | **Pr > F** |
| **TRT** | 1 | 14.8 | 0.01 | 0.9210 |
| **day** | 8 | 171.1 | 14.83 | <.0001 |
| **TRT*day** | 8 | 171.1 | 2.20 | 0.0296 |

| Steps Crossover Repeated Measures Analysis **** save settings of options that will be changed |
| --- |
| PDMIX Mean Separation for count |

Effect=TRT Method=LSD(P<.05) Set=1

| **Obs** | **TRT** | **day** | **Estimate** | **Standard Error** | **Mean** | **Standard Error of Mean** | **Letter Group** |
| --- | --- | --- | --- | --- | --- | --- | --- |
| **1** | Lying | _ | 1999.92 | 224.74 | 1999.92 | 224.74 | A |
| **2** | Sleep | _ | 2010.19 | 224.74 | 2010.19 | 224.74 | A |

# Steps.

| **TRT*day Least Squares Means** | | | | | | | | | | | | | |
| --- | --- | --- | --- | --- | --- | --- | --- | --- | --- | --- | --- | --- | --- |
| **TRT** | **day** | **Estimate** | **Standard Error** | **DF** | **t Value** | **Pr > \|t\|** | **Alpha** | **Lower** | **Upper** | **Mean** | **Standard Error Mean** | **Lower Mean** | **Upper Mean** |
| **Lying** | **0** | 2422.75 | 260.69 | 197 | 9.29 | <.0001 | 0.05 | 1908.64 | 2936.86 | 2422.75 | 260.69 | 1908.64 | 2936.86 |
| **Lying** | **1** | 3318.25 | 260.69 | 197 | 12.73 | <.0001 | 0.05 | 2804.14 | 3832.36 | 3318.25 | 260.69 | 2804.14 | 3832.36 |
| **Lying** | **2** | 1618.83 | 260.69 | 197 | 6.21 | <.0001 | 0.05 | 1104.72 | 2132.94 | 1618.83 | 260.69 | 1104.72 | 2132.94 |
| **Lying** | **3** | 1618.00 | 260.69 | 197 | 6.21 | <.0001 | 0.05 | 1103.89 | 2132.11 | 1618.00 | 260.69 | 1103.89 | 2132.11 |
| **Lying** | **4** | 1686.00 | 260.69 | 197 | 6.47 | <.0001 | 0.05 | 1171.89 | 2200.11 | 1686.00 | 260.69 | 1171.89 | 2200.11 |
| **Lying** | **5** | 2012.83 | 260.69 | 197 | 7.72 | <.0001 | 0.05 | 1498.72 | 2526.94 | 2012.83 | 260.69 | 1498.72 | 2526.94 |
| **Lying** | **6** | 1788.83 | 260.69 | 197 | 6.86 | <.0001 | 0.05 | 1274.72 | 2302.94 | 1788.83 | 260.69 | 1274.72 | 2302.94 |
| **Lying** | **7** | 1805.17 | 260.69 | 197 | 6.92 | <.0001 | 0.05 | 1291.06 | 2319.28 | 1805.17 | 260.69 | 1291.06 | 2319.28 |
| **Lying** | **8** | 1728.58 | 260.69 | 197 | 6.63 | <.0001 | 0.05 | 1214.47 | 2242.69 | 1728.58 | 260.69 | 1214.47 | 2242.69 |
| **Sleep** | **0** | 2623.33 | 287.38 | 197 | 9.13 | <.0001 | 0.05 | 2056.60 | 3190.07 | 2623.33 | 287.38 | 2056.60 | 3190.07 |
| **Sleep** | **1** | 2537.83 | 287.38 | 197 | 8.83 | <.0001 | 0.05 | 1971.10 | 3104.57 | 2537.83 | 287.38 | 1971.10 | 3104.57 |
| **Sleep** | **2** | 2010.33 | 287.38 | 197 | 7.00 | <.0001 | 0.05 | 1443.60 | 2577.07 | 2010.33 | 287.38 | 1443.60 | 2577.07 |
| **Sleep** | **3** | 1828.33 | 287.38 | 197 | 6.36 | <.0001 | 0.05 | 1261.60 | 2395.07 | 1828.33 | 287.38 | 1261.60 | 2395.07 |
| **Sleep** | **4** | 1756.75 | 287.38 | 197 | 6.11 | <.0001 | 0.05 | 1190.01 | 2323.49 | 1756.75 | 287.38 | 1190.01 | 2323.49 |
| **Sleep** | **5** | 1924.50 | 287.38 | 197 | 6.70 | <.0001 | 0.05 | 1357.76 | 2491.24 | 1924.50 | 287.38 | 1357.76 | 2491.24 |
| **Sleep** | **6** | 1819.08 | 287.38 | 197 | 6.33 | <.0001 | 0.05 | 1252.35 | 2385.82 | 1819.08 | 287.38 | 1252.35 | 2385.82 |
| **Sleep** | **7** | 1784.00 | 287.38 | 197 | 6.21 | <.0001 | 0.05 | 1217.26 | 2350.74 | 1784.00 | 287.38 | 1217.26 | 2350.74 |
| **Sleep** | **8** | 1807.58 | 287.38 | 197 | 6.29 | <.0001 | 0.05 | 1240.85 | 2374.32 | 1807.58 | 287.38 | 1240.85 | 2374.32 |


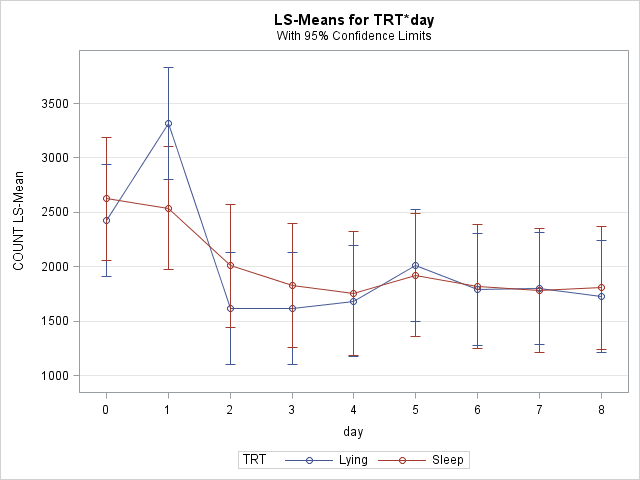


| **Differences of TRT*day Least Squares Means** | | | | | | | | | | | |
| --- | --- | --- | --- | --- | --- | --- | --- | --- | --- | --- | --- |
| **TRT** | **day** | **_TRT** | **_day** | **Estimate** | **Standard Error** | **DF** | **t Value** | **Pr > \|t\|** | **Alpha** | **Lower** | **Upper** |
| **Lying** | **0** | **Lying** | **1** | -895.50 | 192.78 | 197 | -4.65 | <.0001 | 0.05 | -1275.67 | -515.33 |
| **Lying** | **0** | **Lying** | **2** | 803.92 | 192.04 | 197 | 4.19 | <.0001 | 0.05 | 425.19 | 1182.64 |
| **Lying** | **0** | **Lying** | **3** | 804.75 | 191.09 | 197 | 4.21 | <.0001 | 0.05 | 427.90 | 1181.60 |
| **Lying** | **0** | **Lying** | **4** | 736.75 | 191.14 | 197 | 3.85 | 0.0002 | 0.05 | 359.80 | 1113.70 |
| **Lying** | **0** | **Lying** | **5** | 409.92 | 191.14 | 197 | 2.14 | 0.0332 | 0.05 | 32.9745 | 786.86 |
| **Lying** | **0** | **Lying** | **6** | 633.92 | 191.14 | 197 | 3.32 | 0.0011 | 0.05 | 256.97 | 1010.86 |
| **Lying** | **0** | **Lying** | **7** | 617.58 | 191.14 | 197 | 3.23 | 0.0014 | 0.05 | 240.64 | 994.53 |
| **Lying** | **0** | **Lying** | **8** | 694.17 | 191.14 | 197 | 3.63 | 0.0004 | 0.05 | 317.22 | 1071.11 |
| **Lying** | **0** | **Sleep** | **0** | -200.58 | 237.23 | 197 | -0.85 | 0.3988 | 0.05 | -668.42 | 267.25 |
| **Lying** | **0** | **Sleep** | **1** | -115.08 | 237.23 | 197 | -0.49 | 0.6281 | 0.05 | -582.92 | 352.75 |
| **Lying** | **0** | **Sleep** | **2** | 412.42 | 237.23 | 197 | 1.74 | 0.0837 | 0.05 | -55.4184 | 880.25 |
| **Lying** | **0** | **Sleep** | **3** | 594.42 | 237.23 | 197 | 2.51 | 0.0130 | 0.05 | 126.58 | 1062.25 |
| **Lying** | **0** | **Sleep** | **4** | 666.00 | 237.23 | 197 | 2.81 | 0.0055 | 0.05 | 198.16 | 1133.84 |
| **Lying** | **0** | **Sleep** | **5** | 498.25 | 237.23 | 197 | 2.10 | 0.0370 | 0.05 | 30.4149 | 966.09 |
| **Lying** | **0** | **Sleep** | **6** | 603.67 | 237.23 | 197 | 2.54 | 0.0117 | 0.05 | 135.83 | 1071.50 |
| **Lying** | **0** | **Sleep** | **7** | 638.75 | 237.23 | 197 | 2.69 | 0.0077 | 0.05 | 170.91 | 1106.59 |
| **Lying** | **0** | **Sleep** | **8** | 615.17 | 237.23 | 197 | 2.59 | 0.0102 | 0.05 | 147.33 | 1083.00 |
| **Lying** | **1** | **Lying** | **2** | 1699.42 | 192.78 | 197 | 8.82 | <.0001 | 0.05 | 1319.24 | 2079.59 |
| **Lying** | **1** | **Lying** | **3** | 1700.25 | 192.04 | 197 | 8.85 | <.0001 | 0.05 | 1321.52 | 2078.98 |
| **Lying** | **1** | **Lying** | **4** | 1632.25 | 191.09 | 197 | 8.54 | <.0001 | 0.05 | 1255.40 | 2009.10 |
| **Lying** | **1** | **Lying** | **5** | 1305.42 | 191.14 | 197 | 6.83 | <.0001 | 0.05 | 928.47 | 1682.36 |
| **Lying** | **1** | **Lying** | **6** | 1529.42 | 191.14 | 197 | 8.00 | <.0001 | 0.05 | 1152.47 | 1906.36 |
| **Lying** | **1** | **Lying** | **7** | 1513.08 | 191.14 | 197 | 7.92 | <.0001 | 0.05 | 1136.14 | 1890.03 |
| **Lying** | **1** | **Lying** | **8** | 1589.67 | 191.14 | 197 | 8.32 | <.0001 | 0.05 | 1212.72 | 1966.61 |
| **Lying** | **1** | **Sleep** | **0** | 694.92 | 237.23 | 197 | 2.93 | 0.0038 | 0.05 | 227.08 | 1162.75 |
| **Lying** | **1** | **Sleep** | **1** | 780.42 | 237.23 | 197 | 3.29 | 0.0012 | 0.05 | 312.58 | 1248.25 |
| **Lying** | **1** | **Sleep** | **2** | 1307.92 | 237.23 | 197 | 5.51 | <.0001 | 0.05 | 840.08 | 1775.75 |
| **Lying** | **1** | **Sleep** | **3** | 1489.92 | 237.23 | 197 | 6.28 | <.0001 | 0.05 | 1022.08 | 1957.75 |
| **Lying** | **1** | **Sleep** | **4** | 1561.50 | 237.23 | 197 | 6.58 | <.0001 | 0.05 | 1093.66 | 2029.34 |
| **Lying** | **1** | **Sleep** | **5** | 1393.75 | 237.23 | 197 | 5.88 | <.0001 | 0.05 | 925.91 | 1861.59 |
| **Lying** | **1** | **Sleep** | **6** | 1499.17 | 237.23 | 197 | 6.32 | <.0001 | 0.05 | 1031.33 | 1967.00 |
| **Lying** | **1** | **Sleep** | **7** | 1534.25 | 237.23 | 197 | 6.47 | <.0001 | 0.05 | 1066.41 | 2002.09 |
| **Lying** | **1** | **Sleep** | **8** | 1510.67 | 237.23 | 197 | 6.37 | <.0001 | 0.05 | 1042.83 | 1978.50 |
| **Lying** | **2** | **Lying** | **3** | 0.8333 | 192.78 | 197 | 0.00 | 0.9966 | 0.05 | -379.34 | 381.01 |
| **Lying** | **2** | **Lying** | **4** | -67.1667 | 192.04 | 197 | -0.35 | 0.7269 | 0.05 | -445.89 | 311.56 |
| **Lying** | **2** | **Lying** | **5** | -394.00 | 191.09 | 197 | -2.06 | 0.0405 | 0.05 | -770.85 | -17.1520 |
| **Lying** | **2** | **Lying** | **6** | -170.00 | 191.14 | 197 | -0.89 | 0.3749 | 0.05 | -546.95 | 206.95 |
| **Lying** | **2** | **Lying** | **7** | -186.33 | 191.14 | 197 | -0.97 | 0.3308 | 0.05 | -563.28 | 190.61 |
| **Lying** | **2** | **Lying** | **8** | -109.75 | 191.14 | 197 | -0.57 | 0.5665 | 0.05 | -486.69 | 267.19 |
| **Lying** | **2** | **Sleep** | **0** | -1004.50 | 237.23 | 197 | -4.23 | <.0001 | 0.05 | -1472.34 | -536.66 |
| **Lying** | **2** | **Sleep** | **1** | -919.00 | 237.23 | 197 | -3.87 | 0.0001 | 0.05 | -1386.84 | -451.16 |
| **Lying** | **2** | **Sleep** | **2** | -391.50 | 237.23 | 197 | -1.65 | 0.1005 | 0.05 | -859.34 | 76.3351 |
| **Lying** | **2** | **Sleep** | **3** | -209.50 | 237.23 | 197 | -0.88 | 0.3783 | 0.05 | -677.34 | 258.34 |
| **Lying** | **2** | **Sleep** | **4** | -137.92 | 237.23 | 197 | -0.58 | 0.5617 | 0.05 | -605.75 | 329.92 |
| **Lying** | **2** | **Sleep** | **5** | -305.67 | 237.23 | 197 | -1.29 | 0.1991 | 0.05 | -773.50 | 162.17 |
| **Lying** | **2** | **Sleep** | **6** | -200.25 | 237.23 | 197 | -0.84 | 0.3996 | 0.05 | -668.09 | 267.59 |
| **Lying** | **2** | **Sleep** | **7** | -165.17 | 237.23 | 197 | -0.70 | 0.4871 | 0.05 | -633.00 | 302.67 |
| **Lying** | **2** | **Sleep** | **8** | -188.75 | 237.23 | 197 | -0.80 | 0.4272 | 0.05 | -656.59 | 279.09 |
| **Lying** | **3** | **Lying** | **4** | -68.0000 | 192.78 | 197 | -0.35 | 0.7247 | 0.05 | -448.17 | 312.17 |
| **Lying** | **3** | **Lying** | **5** | -394.83 | 192.04 | 197 | -2.06 | 0.0411 | 0.05 | -773.56 | -16.1070 |
| **Lying** | **3** | **Lying** | **6** | -170.83 | 191.09 | 197 | -0.89 | 0.3724 | 0.05 | -547.68 | 206.01 |
| **Lying** | **3** | **Lying** | **7** | -187.17 | 191.14 | 197 | -0.98 | 0.3287 | 0.05 | -564.11 | 189.78 |
| **Lying** | **3** | **Lying** | **8** | -110.58 | 191.14 | 197 | -0.58 | 0.5636 | 0.05 | -487.53 | 266.36 |
| **Lying** | **3** | **Sleep** | **0** | -1005.33 | 237.23 | 197 | -4.24 | <.0001 | 0.05 | -1473.17 | -537.50 |
| **Lying** | **3** | **Sleep** | **1** | -919.83 | 237.23 | 197 | -3.88 | 0.0001 | 0.05 | -1387.67 | -452.00 |
| **Lying** | **3** | **Sleep** | **2** | -392.33 | 237.23 | 197 | -1.65 | 0.0998 | 0.05 | -860.17 | 75.5018 |
| **Lying** | **3** | **Sleep** | **3** | -210.33 | 237.23 | 197 | -0.89 | 0.3764 | 0.05 | -678.17 | 257.50 |
| **Lying** | **3** | **Sleep** | **4** | -138.75 | 237.23 | 197 | -0.58 | 0.5593 | 0.05 | -606.59 | 329.09 |
| **Lying** | **3** | **Sleep** | **5** | -306.50 | 237.23 | 197 | -1.29 | 0.1979 | 0.05 | -774.34 | 161.34 |
| **Lying** | **3** | **Sleep** | **6** | -201.08 | 237.23 | 197 | -0.85 | 0.3977 | 0.05 | -668.92 | 266.75 |
| **Lying** | **3** | **Sleep** | **7** | -166.00 | 237.23 | 197 | -0.70 | 0.4849 | 0.05 | -633.84 | 301.84 |
| **Lying** | **3** | **Sleep** | **8** | -189.58 | 237.23 | 197 | -0.80 | 0.4252 | 0.05 | -657.42 | 278.25 |
| **Lying** | **4** | **Lying** | **5** | -326.83 | 192.78 | 197 | -1.70 | 0.0916 | 0.05 | -707.01 | 53.3408 |
| **Lying** | **4** | **Lying** | **6** | -102.83 | 192.04 | 197 | -0.54 | 0.5929 | 0.05 | -481.56 | 275.89 |
| **Lying** | **4** | **Lying** | **7** | -119.17 | 191.09 | 197 | -0.62 | 0.5336 | 0.05 | -496.01 | 257.68 |
| **Lying** | **4** | **Lying** | **8** | -42.5833 | 191.14 | 197 | -0.22 | 0.8239 | 0.05 | -419.53 | 334.36 |
| **Lying** | **4** | **Sleep** | **0** | -937.33 | 237.23 | 197 | -3.95 | 0.0001 | 0.05 | -1405.17 | -469.50 |
| **Lying** | **4** | **Sleep** | **1** | -851.83 | 237.23 | 197 | -3.59 | 0.0004 | 0.05 | -1319.67 | -384.00 |
| **Lying** | **4** | **Sleep** | **2** | -324.33 | 237.23 | 197 | -1.37 | 0.1731 | 0.05 | -792.17 | 143.50 |
| **Lying** | **4** | **Sleep** | **3** | -142.33 | 237.23 | 197 | -0.60 | 0.5492 | 0.05 | -610.17 | 325.50 |
| **Lying** | **4** | **Sleep** | **4** | -70.7500 | 237.23 | 197 | -0.30 | 0.7658 | 0.05 | -538.59 | 397.09 |
| **Lying** | **4** | **Sleep** | **5** | -238.50 | 237.23 | 197 | -1.01 | 0.3160 | 0.05 | -706.34 | 229.34 |
| **Lying** | **4** | **Sleep** | **6** | -133.08 | 237.23 | 197 | -0.56 | 0.5754 | 0.05 | -600.92 | 334.75 |
| **Lying** | **4** | **Sleep** | **7** | -98.0000 | 237.23 | 197 | -0.41 | 0.6800 | 0.05 | -565.84 | 369.84 |
| **Lying** | **4** | **Sleep** | **8** | -121.58 | 237.23 | 197 | -0.51 | 0.6089 | 0.05 | -589.42 | 346.25 |
| **Lying** | **5** | **Lying** | **6** | 224.00 | 192.78 | 197 | 1.16 | 0.2467 | 0.05 | -156.17 | 604.17 |
| **Lying** | **5** | **Lying** | **7** | 207.67 | 192.04 | 197 | 1.08 | 0.2809 | 0.05 | -171.06 | 586.39 |
| **Lying** | **5** | **Lying** | **8** | 284.25 | 191.09 | 197 | 1.49 | 0.1385 | 0.05 | -92.5980 | 661.10 |
| **Lying** | **5** | **Sleep** | **0** | -610.50 | 237.23 | 197 | -2.57 | 0.0108 | 0.05 | -1078.34 | -142.66 |
| **Lying** | **5** | **Sleep** | **1** | -525.00 | 237.23 | 197 | -2.21 | 0.0280 | 0.05 | -992.84 | -57.1649 |
| **Lying** | **5** | **Sleep** | **2** | 2.5000 | 237.23 | 197 | 0.01 | 0.9916 | 0.05 | -465.34 | 470.34 |
| **Lying** | **5** | **Sleep** | **3** | 184.50 | 237.23 | 197 | 0.78 | 0.4377 | 0.05 | -283.34 | 652.34 |
| **Lying** | **5** | **Sleep** | **4** | 256.08 | 237.23 | 197 | 1.08 | 0.2817 | 0.05 | -211.75 | 723.92 |
| **Lying** | **5** | **Sleep** | **5** | 88.3333 | 237.23 | 197 | 0.37 | 0.7100 | 0.05 | -379.50 | 556.17 |
| **Lying** | **5** | **Sleep** | **6** | 193.75 | 237.23 | 197 | 0.82 | 0.4151 | 0.05 | -274.09 | 661.59 |
| **Lying** | **5** | **Sleep** | **7** | 228.83 | 237.23 | 197 | 0.96 | 0.3359 | 0.05 | -239.00 | 696.67 |
| **Lying** | **5** | **Sleep** | **8** | 205.25 | 237.23 | 197 | 0.87 | 0.3880 | 0.05 | -262.59 | 673.09 |
| **Lying** | **6** | **Lying** | **7** | -16.3333 | 192.78 | 197 | -0.08 | 0.9326 | 0.05 | -396.51 | 363.84 |
| **Lying** | **6** | **Lying** | **8** | 60.2500 | 192.04 | 197 | 0.31 | 0.7541 | 0.05 | -318.48 | 438.98 |
| **Lying** | **6** | **Sleep** | **0** | -834.50 | 237.23 | 197 | -3.52 | 0.0005 | 0.05 | -1302.34 | -366.66 |
| **Lying** | **6** | **Sleep** | **1** | -749.00 | 237.23 | 197 | -3.16 | 0.0018 | 0.05 | -1216.84 | -281.16 |
| **Lying** | **6** | **Sleep** | **2** | -221.50 | 237.23 | 197 | -0.93 | 0.3516 | 0.05 | -689.34 | 246.34 |
| **Lying** | **6** | **Sleep** | **3** | -39.5000 | 237.23 | 197 | -0.17 | 0.8679 | 0.05 | -507.34 | 428.34 |
| **Lying** | **6** | **Sleep** | **4** | 32.0833 | 237.23 | 197 | 0.14 | 0.8926 | 0.05 | -435.75 | 499.92 |
| **Lying** | **6** | **Sleep** | **5** | -135.67 | 237.23 | 197 | -0.57 | 0.5681 | 0.05 | -603.50 | 332.17 |
| **Lying** | **6** | **Sleep** | **6** | -30.2500 | 237.23 | 197 | -0.13 | 0.8987 | 0.05 | -498.09 | 437.59 |
| **Lying** | **6** | **Sleep** | **7** | 4.8333 | 237.23 | 197 | 0.02 | 0.9838 | 0.05 | -463.00 | 472.67 |
| **Lying** | **6** | **Sleep** | **8** | -18.7500 | 237.23 | 197 | -0.08 | 0.9371 | 0.05 | -486.59 | 449.09 |
| **Lying** | **7** | **Lying** | **8** | 76.5833 | 192.78 | 197 | 0.40 | 0.6916 | 0.05 | -303.59 | 456.76 |
| **Lying** | **7** | **Sleep** | **0** | -818.17 | 237.23 | 197 | -3.45 | 0.0007 | 0.05 | -1286.00 | -350.33 |
| **Lying** | **7** | **Sleep** | **1** | -732.67 | 237.23 | 197 | -3.09 | 0.0023 | 0.05 | -1200.50 | -264.83 |
| **Lying** | **7** | **Sleep** | **2** | -205.17 | 237.23 | 197 | -0.86 | 0.3882 | 0.05 | -673.00 | 262.67 |
| **Lying** | **7** | **Sleep** | **3** | -23.1667 | 237.23 | 197 | -0.10 | 0.9223 | 0.05 | -491.00 | 444.67 |
| **Lying** | **7** | **Sleep** | **4** | 48.4167 | 237.23 | 197 | 0.20 | 0.8385 | 0.05 | -419.42 | 516.25 |
| **Lying** | **7** | **Sleep** | **5** | -119.33 | 237.23 | 197 | -0.50 | 0.6155 | 0.05 | -587.17 | 348.50 |
| **Lying** | **7** | **Sleep** | **6** | -13.9167 | 237.23 | 197 | -0.06 | 0.9533 | 0.05 | -481.75 | 453.92 |
| **Lying** | **7** | **Sleep** | **7** | 21.1667 | 237.23 | 197 | 0.09 | 0.9290 | 0.05 | -446.67 | 489.00 |
| **Lying** | **7** | **Sleep** | **8** | -2.4167 | 237.23 | 197 | -0.01 | 0.9919 | 0.05 | -470.25 | 465.42 |
| **Lying** | **8** | **Sleep** | **0** | -894.75 | 237.23 | 197 | -3.77 | 0.0002 | 0.05 | -1362.59 | -426.91 |
| **Lying** | **8** | **Sleep** | **1** | -809.25 | 237.23 | 197 | -3.41 | 0.0008 | 0.05 | -1277.09 | -341.41 |
| **Lying** | **8** | **Sleep** | **2** | -281.75 | 237.23 | 197 | -1.19 | 0.2364 | 0.05 | -749.59 | 186.09 |
| **Lying** | **8** | **Sleep** | **3** | -99.7500 | 237.23 | 197 | -0.42 | 0.6746 | 0.05 | -567.59 | 368.09 |
| **Lying** | **8** | **Sleep** | **4** | -28.1667 | 237.23 | 197 | -0.12 | 0.9056 | 0.05 | -496.00 | 439.67 |
| **Lying** | **8** | **Sleep** | **5** | -195.92 | 237.23 | 197 | -0.83 | 0.4099 | 0.05 | -663.75 | 271.92 |
| **Lying** | **8** | **Sleep** | **6** | -90.5000 | 237.23 | 197 | -0.38 | 0.7033 | 0.05 | -558.34 | 377.34 |
| **Lying** | **8** | **Sleep** | **7** | -55.4167 | 237.23 | 197 | -0.23 | 0.8155 | 0.05 | -523.25 | 412.42 |
| **Lying** | **8** | **Sleep** | **8** | -79.0000 | 237.23 | 197 | -0.33 | 0.7395 | 0.05 | -546.84 | 388.84 |
| **Sleep** | **0** | **Sleep** | **1** | 85.5000 | 153.84 | 80.17 | 0.56 | 0.5799 | 0.05 | -220.64 | 391.64 |
| **Sleep** | **0** | **Sleep** | **2** | 613.00 | 197.56 | 131.5 | 3.10 | 0.0023 | 0.05 | 222.20 | 1003.80 |
| **Sleep** | **0** | **Sleep** | **3** | 795.00 | 221.19 | 197 | 3.59 | 0.0004 | 0.05 | 358.79 | 1231.21 |
| **Sleep** | **0** | **Sleep** | **4** | 866.58 | 235.06 | 197 | 3.69 | 0.0003 | 0.05 | 403.04 | 1330.13 |
| **Sleep** | **0** | **Sleep** | **5** | 698.83 | 243.44 | 197 | 2.87 | 0.0045 | 0.05 | 218.75 | 1178.91 |
| **Sleep** | **0** | **Sleep** | **6** | 804.25 | 248.57 | 197 | 3.24 | 0.0014 | 0.05 | 314.05 | 1294.45 |
| **Sleep** | **0** | **Sleep** | **7** | 839.33 | 251.72 | 197 | 3.33 | 0.0010 | 0.05 | 342.92 | 1335.74 |
| **Sleep** | **0** | **Sleep** | **8** | 815.75 | 253.65 | 197 | 3.22 | 0.0015 | 0.05 | 315.54 | 1315.96 |
| **Sleep** | **1** | **Sleep** | **2** | 527.50 | 153.84 | 80.17 | 3.43 | 0.0010 | 0.05 | 221.36 | 833.64 |
| **Sleep** | **1** | **Sleep** | **3** | 709.50 | 197.56 | 131.5 | 3.59 | 0.0005 | 0.05 | 318.70 | 1100.30 |
| **Sleep** | **1** | **Sleep** | **4** | 781.08 | 221.19 | 197 | 3.53 | 0.0005 | 0.05 | 344.88 | 1217.29 |
| **Sleep** | **1** | **Sleep** | **5** | 613.33 | 235.06 | 197 | 2.61 | 0.0098 | 0.05 | 149.79 | 1076.88 |
| **Sleep** | **1** | **Sleep** | **6** | 718.75 | 243.44 | 197 | 2.95 | 0.0035 | 0.05 | 238.67 | 1198.83 |
| **Sleep** | **1** | **Sleep** | **7** | 753.83 | 248.57 | 197 | 3.03 | 0.0028 | 0.05 | 263.63 | 1244.03 |
| **Sleep** | **1** | **Sleep** | **8** | 730.25 | 251.72 | 197 | 2.90 | 0.0041 | 0.05 | 233.84 | 1226.66 |
| **Sleep** | **2** | **Sleep** | **3** | 182.00 | 153.84 | 80.17 | 1.18 | 0.2403 | 0.05 | -124.14 | 488.14 |
| **Sleep** | **2** | **Sleep** | **4** | 253.58 | 197.56 | 131.5 | 1.28 | 0.2015 | 0.05 | -137.22 | 644.39 |
| **Sleep** | **2** | **Sleep** | **5** | 85.8333 | 221.19 | 197 | 0.39 | 0.6984 | 0.05 | -350.37 | 522.04 |
| **Sleep** | **2** | **Sleep** | **6** | 191.25 | 235.06 | 197 | 0.81 | 0.4168 | 0.05 | -272.30 | 654.80 |
| **Sleep** | **2** | **Sleep** | **7** | 226.33 | 243.44 | 197 | 0.93 | 0.3536 | 0.05 | -253.75 | 706.41 |
| **Sleep** | **2** | **Sleep** | **8** | 202.75 | 248.57 | 197 | 0.82 | 0.4157 | 0.05 | -287.45 | 692.95 |
| **Sleep** | **3** | **Sleep** | **4** | 71.5833 | 153.84 | 80.17 | 0.47 | 0.6430 | 0.05 | -234.55 | 377.72 |
| **Sleep** | **3** | **Sleep** | **5** | -96.1667 | 197.56 | 131.5 | -0.49 | 0.6272 | 0.05 | -486.97 | 294.64 |
| **Sleep** | **3** | **Sleep** | **6** | 9.2500 | 221.19 | 197 | 0.04 | 0.9667 | 0.05 | -426.96 | 445.46 |
| **Sleep** | **3** | **Sleep** | **7** | 44.3333 | 235.06 | 197 | 0.19 | 0.8506 | 0.05 | -419.21 | 507.88 |
| **Sleep** | **3** | **Sleep** | **8** | 20.7500 | 243.44 | 197 | 0.09 | 0.9322 | 0.05 | -459.33 | 500.83 |
| **Sleep** | **4** | **Sleep** | **5** | -167.75 | 153.84 | 80.17 | -1.09 | 0.2788 | 0.05 | -473.89 | 138.39 |
| **Sleep** | **4** | **Sleep** | **6** | -62.3333 | 197.56 | 131.5 | -0.32 | 0.7529 | 0.05 | -453.14 | 328.47 |
| **Sleep** | **4** | **Sleep** | **7** | -27.2500 | 221.19 | 197 | -0.12 | 0.9021 | 0.05 | -463.46 | 408.96 |
| **Sleep** | **4** | **Sleep** | **8** | -50.8333 | 235.06 | 197 | -0.22 | 0.8290 | 0.05 | -514.38 | 412.71 |
| **Sleep** | **5** | **Sleep** | **6** | 105.42 | 153.84 | 80.17 | 0.69 | 0.4952 | 0.05 | -200.72 | 411.55 |
| **Sleep** | **5** | **Sleep** | **7** | 140.50 | 197.56 | 131.5 | 0.71 | 0.4782 | 0.05 | -250.30 | 531.30 |
| **Sleep** | **5** | **Sleep** | **8** | 116.92 | 221.19 | 197 | 0.53 | 0.5977 | 0.05 | -319.29 | 553.12 |
| **Sleep** | **6** | **Sleep** | **7** | 35.0833 | 153.84 | 80.17 | 0.23 | 0.8202 | 0.05 | -271.05 | 341.22 |
| **Sleep** | **6** | **Sleep** | **8** | 11.5000 | 197.56 | 131.5 | 0.06 | 0.9537 | 0.05 | -379.30 | 402.30 |
| **Sleep** | **7** | **Sleep** | **8** | -23.5833 | 153.84 | 80.17 | -0.15 | 0.8785 | 0.05 | -329.72 | 282.55 |
