# Supplementary material for: Effects of acute lying and sleep deprivation on the behavior of lactating dairy cows
Source: PLoS One. 2019 Aug 28;14(8):e0212823. doi: 10.1371/journal.pone.0212823 (PMC6713338; doi:10.1371/journal.pone.0212823)
Supplement: S2 File — Supplemental data to support conclusions drawn on the effects of treatment on the daily lying time of cows. (DOCX) [file pone.0212823.s004.docx]

Lying time day and trt*day information

| **Type III Tests of Fixed Effects** | | | | |
| --- | --- | --- | --- | --- |
| **Effect** | **Num DF** | **Den DF** | **F Value** | **Pr > F** |
| **Period2** | 1 | 10.03 | 0.06 | 0.8115 |
| **TRT** | 1 | 10.42 | 0.12 | 0.7364 |
| **day** | 8 | 125.5 | 47.43 | <.0001 |
| **TRT*day** | 8 | 125.5 | 8.41 | <.0001 |

| **TRT Least Squares Means** | | | | | | | | | | | | |
| --- | --- | --- | --- | --- | --- | --- | --- | --- | --- | --- | --- | --- |
| **TRT** | **Estimate** | **Standard Error** | **DF** | **t Value** | **Pr > \|t\|** | **Alpha** | **Lower** | **Upper** | **Mean** | **Standard Error Mean** | **Lower Mean** | **Upper Mean** |
| **Lying** | 11.2918 | 0.3493 | 11.28 | 32.33 | <.0001 | 0.05 | 10.5254 | 12.0582 | 11.2918 | 0.3493 | 10.5254 | 12.0582 |
| **Sleep** | 11.4193 | 0.4244 | 11.39 | 26.91 | <.0001 | 0.05 | 10.4891 | 12.3494 | 11.4193 | 0.4244 | 10.4891 | 12.3494 |


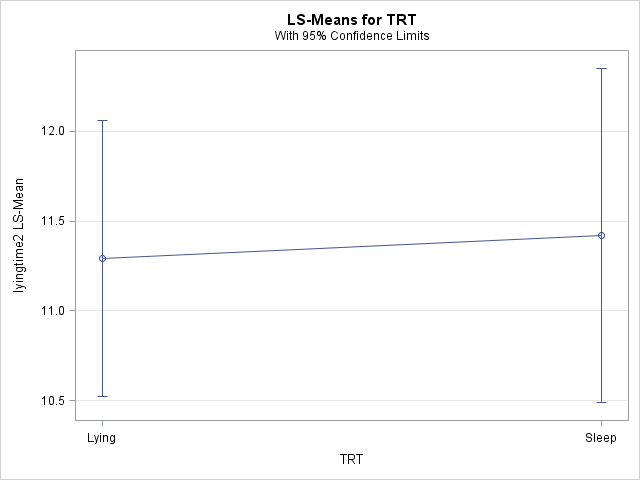


| **Differences of TRT Least Squares Means** | | | | | | | | | |
| --- | --- | --- | --- | --- | --- | --- | --- | --- | --- |
| **TRT** | **_TRT** | **Estimate** | **Standard Error** | **DF** | **t Value** | **Pr > \|t\|** | **Alpha** | **Lower** | **Upper** |
| **Lying** | **Sleep** | -0.1275 | 0.3686 | 10.42 | -0.35 | 0.7364 | 0.05 | -0.9443 | 0.6894 |

| **day Least Squares Means** | | | | | | | | | | | | |
| --- | --- | --- | --- | --- | --- | --- | --- | --- | --- | --- | --- | --- |
| **day** | **Estimate** | **Standard Error** | **DF** | **t Value** | **Pr > \|t\|** | **Alpha** | **Lower** | **Upper** | **Mean** | **Standard Error Mean** | **Lower Mean** | **Upper Mean** |
| **0** | 8.7083 | 0.5043 | 45.07 | 17.27 | <.0001 | 0.05 | 7.6927 | 9.7239 | 8.7083 | 0.5043 | 7.6927 | 9.7239 |
| **1** | 5.1251 | 0.5654 | 63.08 | 9.06 | <.0001 | 0.05 | 3.9953 | 6.2549 | 5.1251 | 0.5654 | 3.9953 | 6.2549 |
| **2** | 15.2250 | 0.5043 | 45.07 | 30.19 | <.0001 | 0.05 | 14.2094 | 16.2406 | 15.2250 | 0.5043 | 14.2094 | 16.2406 |
| **3** | 13.3479 | 0.5043 | 45.07 | 26.47 | <.0001 | 0.05 | 12.3323 | 14.3635 | 13.3479 | 0.5043 | 12.3323 | 14.3635 |
| **4** | 12.8371 | 0.5043 | 45.07 | 25.46 | <.0001 | 0.05 | 11.8215 | 13.8527 | 12.8371 | 0.5043 | 11.8215 | 13.8527 |
| **5** | 11.7404 | 0.5043 | 45.07 | 23.28 | <.0001 | 0.05 | 10.7248 | 12.7560 | 11.7404 | 0.5043 | 10.7248 | 12.7560 |
| **6** | 11.7913 | 0.5043 | 45.07 | 23.38 | <.0001 | 0.05 | 10.7756 | 12.8069 | 11.7913 | 0.5043 | 10.7756 | 12.8069 |
| **7** | 12.1213 | 0.5043 | 45.07 | 24.04 | <.0001 | 0.05 | 11.1056 | 13.1369 | 12.1213 | 0.5043 | 11.1056 | 13.1369 |
| **8** | 11.3033 | 0.5043 | 45.07 | 22.42 | <.0001 | 0.05 | 10.2877 | 12.3189 | 11.3033 | 0.5043 | 10.2877 | 12.3189 |


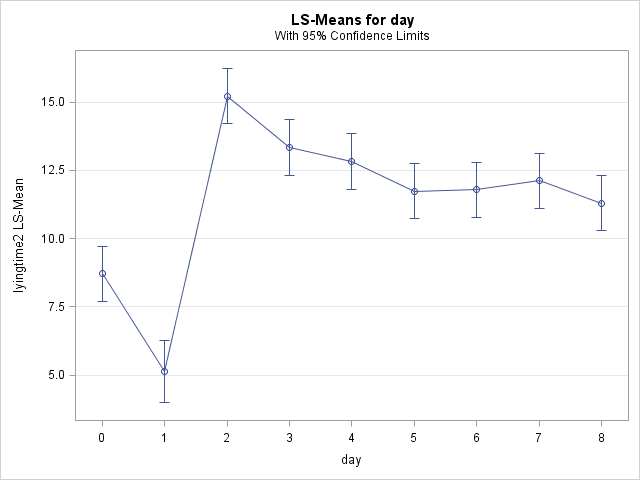


| **Differences of day Least Squares Means** | | | | | | | | | | | | | | | | | | | | | |  |
| --- | --- | --- | --- | --- | --- | --- | --- | --- | --- | --- | --- | --- | --- | --- | --- | --- | --- | --- | --- | --- | --- | --- |
| **day** | **_day** | | | **Estimate** | | **Standard Error** | | | | **DF** | | **t Value** | | **Pr > \|t\|** | | **Alpha** | | **Lower** | | **Upper** | |  |
| **0** | **1** | | | 3.5832 | | 0.6248 | | | | 116.5 | | 5.74 | | <.0001 | | 0.05 | | 2.3458 | | 4.8206 | |  |
| **0** | **2** | | | -6.5167 | | 0.5275 | | | | 146.5 | | -12.35 | | <.0001 | | 0.05 | | -7.5591 | | -5.4742 | |  |
| **0** | **3** | | | -4.6396 | | 0.5601 | | | | 121.1 | | -8.28 | | <.0001 | | 0.05 | | -5.7484 | | -3.5307 | |  |
| **0** | **4** | | | -4.1288 | | 0.5567 | | | | 143.6 | | -7.42 | | <.0001 | | 0.05 | | -5.2291 | | -3.0284 | |  |
| **0** | **5** | | | -3.0321 | | 0.5593 | | | | 134 | | -5.42 | | <.0001 | | 0.05 | | -4.1383 | | -1.9259 | |  |
| **0** | **6** | | | -3.0829 | | 0.5712 | | | | 138.7 | | -5.40 | | <.0001 | | 0.05 | | -4.2123 | | -1.9536 | |  |
| **0** | **7** | | | -3.4129 | | 0.5644 | | | | 136.5 | | -6.05 | | <.0001 | | 0.05 | | -4.5290 | | -2.2968 | |  |
| **0** | **8** | | | -2.5950 | | 0.5779 | | | | 134.2 | | -4.49 | | <.0001 | | 0.05 | | -3.7380 | | -1.4520 | |  |
| **1** | **2** | | | -10.0999 | | 0.6248 | | | | 116.5 | | -16.17 | | <.0001 | | 0.05 | | -11.3373 | | -8.8625 | |  |
| **1** | **3** | | | -8.2228 | | 0.5862 | | | | 146.9 | | -14.03 | | <.0001 | | 0.05 | | -9.3813 | | -7.0643 | |  |
| **1** | **4** | | | -7.7120 | | 0.6157 | | | | 135.5 | | -12.53 | | <.0001 | | 0.05 | | -8.9296 | | -6.4943 | |  |
| **1** | **5** | | | -6.6153 | | 0.6126 | | | | 146.2 | | -10.80 | | <.0001 | | 0.05 | | -7.8260 | | -5.4046 | |  |
| **1** | **6** | | | -6.6661 | | 0.6150 | | | | 148.8 | | -10.84 | | <.0001 | | 0.05 | | -7.8813 | | -5.4509 | |  |
| **1** | **7** | | | -6.9961 | | 0.6258 | | | | 146 | | -11.18 | | <.0001 | | 0.05 | | -8.2329 | | -5.7593 | |  |
| **1** | **8** | | | -6.1782 | | 0.6196 | | | | 151 | | -9.97 | | <.0001 | | 0.05 | | -7.4025 | | -4.9540 | |  |
| **2** | **3** | | | 1.8771 | | 0.5701 | | | | 108.6 | | 3.29 | | 0.0013 | | 0.05 | | 0.7472 | | 3.0070 | |  |
| **2** | **4** | | | 2.3879 | | 0.5275 | | | | 146.5 | | 4.53 | | <.0001 | | 0.05 | | 1.3455 | | 3.4304 | |  |
| **2** | **5** | | | 3.4846 | | 0.5601 | | | | 121.1 | | 6.22 | | <.0001 | | 0.05 | | 2.3757 | | 4.5934 | |  |
| **2** | **6** | | | 3.4338 | | 0.5567 | | | | 143.6 | | 6.17 | | <.0001 | | 0.05 | | 2.3334 | | 4.5341 | |  |
| **2** | **7** | | | 3.1038 | | 0.5593 | | | | 134 | | 5.55 | | <.0001 | | 0.05 | | 1.9976 | | 4.2099 | |  |
| **2** | **8** | | | 3.9217 | | 0.5712 | | | | 138.7 | | 6.87 | | <.0001 | | 0.05 | | 2.7923 | | 5.0510 | |  |
| **3** | **4** | | | 0.5108 | | 0.5701 | | | | 108.6 | | 0.90 | | 0.3722 | | 0.05 | | -0.6191 | | 1.6407 | |  |
| **3** | **5** | | | 1.6075 | | 0.5275 | | | | 146.5 | | 3.05 | | 0.0027 | | 0.05 | | 0.5650 | | 2.6500 | |  |
| **3** | **6** | | | 1.5567 | | 0.5601 | | | | 121.1 | | 2.78 | | 0.0063 | | 0.05 | | 0.4478 | | 2.6655 | |  |
| **3** | **7** | | | 1.2267 | | 0.5567 | | | | 143.6 | | 2.20 | | 0.0291 | | 0.05 | | 0.1263 | | 2.3270 | |  |
| **3** | **8** | | | 2.0446 | | 0.5593 | | | | 134 | | 3.66 | | 0.0004 | | 0.05 | | 0.9384 | | 3.1508 | |  |
| **4** | **5** | | | 1.0967 | | 0.5701 | | | | 108.6 | | 1.92 | | 0.0570 | | 0.05 | | -0.03322 | | 2.2266 | |  |
| **4** | **6** | | | 1.0458 | | 0.5275 | | | | 146.5 | | 1.98 | | 0.0493 | | 0.05 | | 0.003374 | | 2.0883 | |  |
| **4** | **7** | | | 0.7158 | | 0.5601 | | | | 121.1 | | 1.28 | | 0.2037 | | 0.05 | | -0.3930 | | 1.8247 | |  |
| **4** | **8** | | | 1.5338 | | 0.5567 | | | | 143.6 | | 2.76 | | 0.0066 | | 0.05 | | 0.4334 | | 2.6341 | |  |
| **5** | **6** | | | -0.05083 | | 0.5701 | | | | 108.6 | | -0.09 | | 0.9291 | | 0.05 | | -1.1807 | | 1.0791 | |  |
| **5** | **7** | | | -0.3808 | | 0.5275 | | | | 146.5 | | -0.72 | | 0.4715 | | 0.05 | | -1.4233 | | 0.6616 | |  |
| **5** | **8** | | | 0.4371 | | 0.5601 | | | | 121.1 | | 0.78 | | 0.4367 | | 0.05 | | -0.6718 | | 1.5459 | |  |
| **6** | **7** | | | -0.3300 | | 0.5701 | | | | 108.6 | | -0.58 | | 0.5639 | | 0.05 | | -1.4599 | | 0.7999 | |  |
| **6** | **8** | | | 0.4879 | | 0.5275 | | | | 146.5 | | 0.92 | | 0.3565 | | 0.05 | | -0.5545 | | 1.5304 | |  |
| **7** | **8** | | | 0.8179 | | 0.5701 | | | | 108.6 | | 1.43 | | 0.1542 | | 0.05 | | -0.3120 | | 1.9478 | |  |
| **TRT*day Least Squares Means** | | | | | | | | | | | | | | | | | | | | | | |
| **TRT** | | **day** | **Estimate** | | **Standard Error** | | **DF** | **t Value** | **Pr > \|t\|** | | **Alpha** | | **Lower** | | **Upper** | | **Mean** | | **Standard Error Mean** | | **Lower Mean** | **Upper Mean** |
| **Lying** | | **0** | 8.7842 | | 0.6483 | | 70.87 | 13.55 | <.0001 | | 0.05 | | 7.4916 | | 10.0768 | | 8.7842 | | 0.6483 | | 7.4916 | 10.0768 |
| **Lying** | | **1** | 1.8802 | | 0.8257 | | 95.45 | 2.28 | 0.0250 | | 0.05 | | 0.2412 | | 3.5193 | | 1.8802 | | 0.8257 | | 0.2412 | 3.5193 |
| **Lying** | | **2** | 16.8125 | | 0.6483 | | 70.87 | 25.94 | <.0001 | | 0.05 | | 15.5199 | | 18.1051 | | 16.8125 | | 0.6483 | | 15.5199 | 18.1051 |
| **Lying** | | **3** | 13.9883 | | 0.6483 | | 70.87 | 21.58 | <.0001 | | 0.05 | | 12.6957 | | 15.2809 | | 13.9883 | | 0.6483 | | 12.6957 | 15.2809 |
| **Lying** | | **4** | 12.8083 | | 0.6483 | | 70.87 | 19.76 | <.0001 | | 0.05 | | 11.5157 | | 14.1009 | | 12.8083 | | 0.6483 | | 11.5157 | 14.1009 |
| **Lying** | | **5** | 11.8983 | | 0.6483 | | 70.87 | 18.35 | <.0001 | | 0.05 | | 10.6057 | | 13.1909 | | 11.8983 | | 0.6483 | | 10.6057 | 13.1909 |
| **Lying** | | **6** | 11.8342 | | 0.6483 | | 70.87 | 18.26 | <.0001 | | 0.05 | | 10.5416 | | 13.1268 | | 11.8342 | | 0.6483 | | 10.5416 | 13.1268 |
| **Lying** | | **7** | 12.2150 | | 0.6483 | | 70.87 | 18.84 | <.0001 | | 0.05 | | 10.9224 | | 13.5076 | | 12.2150 | | 0.6483 | | 10.9224 | 13.5076 |
| **Lying** | | **8** | 11.4050 | | 0.6483 | | 70.87 | 17.59 | <.0001 | | 0.05 | | 10.1124 | | 12.6976 | | 11.4050 | | 0.6483 | | 10.1124 | 12.6976 |
| **Sleep** | | **0** | 8.6325 | | 0.6563 | | 47.4 | 13.15 | <.0001 | | 0.05 | | 7.3125 | | 9.9525 | | 8.6325 | | 0.6563 | | 7.3125 | 9.9525 |
| **Sleep** | | **1** | 8.3700 | | 0.6563 | | 47.4 | 12.75 | <.0001 | | 0.05 | | 7.0500 | | 9.6900 | | 8.3700 | | 0.6563 | | 7.0500 | 9.6900 |
| **Sleep** | | **2** | 13.6375 | | 0.6563 | | 47.4 | 20.78 | <.0001 | | 0.05 | | 12.3175 | | 14.9575 | | 13.6375 | | 0.6563 | | 12.3175 | 14.9575 |
| **Sleep** | | **3** | 12.7075 | | 0.6563 | | 47.4 | 19.36 | <.0001 | | 0.05 | | 11.3875 | | 14.0275 | | 12.7075 | | 0.6563 | | 11.3875 | 14.0275 |
| **Sleep** | | **4** | 12.8658 | | 0.6563 | | 47.4 | 19.60 | <.0001 | | 0.05 | | 11.5458 | | 14.1858 | | 12.8658 | | 0.6563 | | 11.5458 | 14.1858 |
| **Sleep** | | **5** | 11.5825 | | 0.6563 | | 47.4 | 17.65 | <.0001 | | 0.05 | | 10.2625 | | 12.9025 | | 11.5825 | | 0.6563 | | 10.2625 | 12.9025 |
| **Sleep** | | **6** | 11.7483 | | 0.6563 | | 47.4 | 17.90 | <.0001 | | 0.05 | | 10.4283 | | 13.0683 | | 11.7483 | | 0.6563 | | 10.4283 | 13.0683 |
| **Sleep** | | **7** | 12.0275 | | 0.6563 | | 47.4 | 18.33 | <.0001 | | 0.05 | | 10.7075 | | 13.3475 | | 12.0275 | | 0.6563 | | 10.7075 | 13.3475 |
| **Sleep** | | **8** | 11.2017 | | 0.6563 | | 47.4 | 17.07 | <.0001 | | 0.05 | | 9.8817 | | 12.5217 | | 11.2017 | | 0.6563 | | 9.8817 | 12.5217 |


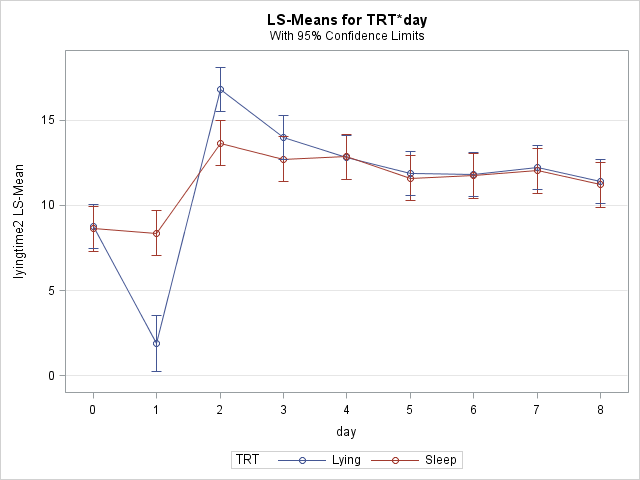


| **Differences of TRT*day Least Squares Means** | | | | | | | | | | | |
| --- | --- | --- | --- | --- | --- | --- | --- | --- | --- | --- | --- |
| **TRT** | **day** | **_TRT** | **_day** | **Estimate** | **Standard Error** | **DF** | **t Value** | **Pr > \|t\|** | **Alpha** | **Lower** | **Upper** |
| **Lying** | **0** | **Lying** | **1** | 6.9039 | 1.0282 | 63.2 | 6.71 | <.0001 | 0.05 | 4.8494 | 8.9585 |
| **Lying** | **0** | **Lying** | **2** | -8.0283 | 0.7606 | 71.13 | -10.56 | <.0001 | 0.05 | -9.5448 | -6.5118 |
| **Lying** | **0** | **Lying** | **3** | -5.2042 | 0.8327 | 59.93 | -6.25 | <.0001 | 0.05 | -6.8699 | -3.5385 |
| **Lying** | **0** | **Lying** | **4** | -4.0242 | 0.8089 | 69.05 | -4.97 | <.0001 | 0.05 | -5.6379 | -2.4105 |
| **Lying** | **0** | **Lying** | **5** | -3.1142 | 0.8032 | 73.72 | -3.88 | 0.0002 | 0.05 | -4.7147 | -1.5137 |
| **Lying** | **0** | **Lying** | **6** | -3.0500 | 0.8250 | 78.33 | -3.70 | 0.0004 | 0.05 | -4.6924 | -1.4076 |
| **Lying** | **0** | **Lying** | **7** | -3.4308 | 0.7962 | 84.04 | -4.31 | <.0001 | 0.05 | -5.0142 | -1.8474 |
| **Lying** | **0** | **Lying** | **8** | -2.6208 | 0.8260 | 84.39 | -3.17 | 0.0021 | 0.05 | -4.2633 | -0.9783 |
| **Lying** | **0** | **Sleep** | **0** | 0.1517 | 0.8275 | 117.8 | 0.18 | 0.8549 | 0.05 | -1.4870 | 1.7904 |
| **Lying** | **0** | **Sleep** | **1** | 0.4142 | 0.8275 | 117.8 | 0.50 | 0.6177 | 0.05 | -1.2245 | 2.0529 |
| **Lying** | **0** | **Sleep** | **2** | -4.8533 | 0.8275 | 117.8 | -5.87 | <.0001 | 0.05 | -6.4920 | -3.2146 |
| **Lying** | **0** | **Sleep** | **3** | -3.9233 | 0.8275 | 117.8 | -4.74 | <.0001 | 0.05 | -5.5620 | -2.2846 |
| **Lying** | **0** | **Sleep** | **4** | -4.0817 | 0.8275 | 117.8 | -4.93 | <.0001 | 0.05 | -5.7204 | -2.4430 |
| **Lying** | **0** | **Sleep** | **5** | -2.7983 | 0.8275 | 117.8 | -3.38 | 0.0010 | 0.05 | -4.4370 | -1.1596 |
| **Lying** | **0** | **Sleep** | **6** | -2.9642 | 0.8275 | 117.8 | -3.58 | 0.0005 | 0.05 | -4.6029 | -1.3255 |
| **Lying** | **0** | **Sleep** | **7** | -3.2433 | 0.8275 | 117.8 | -3.92 | 0.0001 | 0.05 | -4.8820 | -1.6046 |
| **Lying** | **0** | **Sleep** | **8** | -2.4175 | 0.8275 | 117.8 | -2.92 | 0.0042 | 0.05 | -4.0562 | -0.7788 |
| **Lying** | **1** | **Lying** | **2** | -14.9323 | 1.0282 | 63.2 | -14.52 | <.0001 | 0.05 | -16.9868 | -12.8777 |
| **Lying** | **1** | **Lying** | **3** | -12.1081 | 0.9165 | 81.8 | -13.21 | <.0001 | 0.05 | -13.9314 | -10.2848 |
| **Lying** | **1** | **Lying** | **4** | -10.9281 | 0.9772 | 78.17 | -11.18 | <.0001 | 0.05 | -12.8735 | -8.9827 |
| **Lying** | **1** | **Lying** | **5** | -10.0181 | 0.9570 | 78.74 | -10.47 | <.0001 | 0.05 | -11.9230 | -8.1131 |
| **Lying** | **1** | **Lying** | **6** | -9.9539 | 0.9522 | 90.31 | -10.45 | <.0001 | 0.05 | -11.8455 | -8.0623 |
| **Lying** | **1** | **Lying** | **7** | -10.3348 | 0.9707 | 86.11 | -10.65 | <.0001 | 0.05 | -12.2643 | -8.4052 |
| **Lying** | **1** | **Lying** | **8** | -9.5248 | 0.9463 | 96.53 | -10.06 | <.0001 | 0.05 | -11.4031 | -7.6464 |
| **Lying** | **1** | **Sleep** | **0** | -6.7523 | 0.9728 | 136.9 | -6.94 | <.0001 | 0.05 | -8.6759 | -4.8287 |
| **Lying** | **1** | **Sleep** | **1** | -6.4898 | 0.9728 | 136.9 | -6.67 | <.0001 | 0.05 | -8.4134 | -4.5662 |
| **Lying** | **1** | **Sleep** | **2** | -11.7573 | 0.9728 | 136.9 | -12.09 | <.0001 | 0.05 | -13.6809 | -9.8337 |
| **Lying** | **1** | **Sleep** | **3** | -10.8273 | 0.9728 | 136.9 | -11.13 | <.0001 | 0.05 | -12.7509 | -8.9037 |
| **Lying** | **1** | **Sleep** | **4** | -10.9856 | 0.9728 | 136.9 | -11.29 | <.0001 | 0.05 | -12.9092 | -9.0620 |
| **Lying** | **1** | **Sleep** | **5** | -9.7023 | 0.9728 | 136.9 | -9.97 | <.0001 | 0.05 | -11.6259 | -7.7787 |
| **Lying** | **1** | **Sleep** | **6** | -9.8681 | 0.9728 | 136.9 | -10.14 | <.0001 | 0.05 | -11.7917 | -7.9445 |
| **Lying** | **1** | **Sleep** | **7** | -10.1473 | 0.9728 | 136.9 | -10.43 | <.0001 | 0.05 | -12.0709 | -8.2237 |
| **Lying** | **1** | **Sleep** | **8** | -9.3214 | 0.9728 | 136.9 | -9.58 | <.0001 | 0.05 | -11.2450 | -7.3978 |
| **Lying** | **2** | **Lying** | **3** | 2.8242 | 0.8920 | 49.19 | 3.17 | 0.0027 | 0.05 | 1.0318 | 4.6165 |
| **Lying** | **2** | **Lying** | **4** | 4.0042 | 0.7606 | 71.13 | 5.26 | <.0001 | 0.05 | 2.4877 | 5.5207 |
| **Lying** | **2** | **Lying** | **5** | 4.9142 | 0.8327 | 59.93 | 5.90 | <.0001 | 0.05 | 3.2485 | 6.5799 |
| **Lying** | **2** | **Lying** | **6** | 4.9783 | 0.8089 | 69.05 | 6.15 | <.0001 | 0.05 | 3.3646 | 6.5920 |
| **Lying** | **2** | **Lying** | **7** | 4.5975 | 0.8032 | 73.72 | 5.72 | <.0001 | 0.05 | 2.9970 | 6.1980 |
| **Lying** | **2** | **Lying** | **8** | 5.4075 | 0.8250 | 78.33 | 6.55 | <.0001 | 0.05 | 3.7651 | 7.0499 |
| **Lying** | **2** | **Sleep** | **0** | 8.1800 | 0.8275 | 117.8 | 9.89 | <.0001 | 0.05 | 6.5413 | 9.8187 |
| **Lying** | **2** | **Sleep** | **1** | 8.4425 | 0.8275 | 117.8 | 10.20 | <.0001 | 0.05 | 6.8038 | 10.0812 |
| **Lying** | **2** | **Sleep** | **2** | 3.1750 | 0.8275 | 117.8 | 3.84 | 0.0002 | 0.05 | 1.5363 | 4.8137 |
| **Lying** | **2** | **Sleep** | **3** | 4.1050 | 0.8275 | 117.8 | 4.96 | <.0001 | 0.05 | 2.4663 | 5.7437 |
| **Lying** | **2** | **Sleep** | **4** | 3.9467 | 0.8275 | 117.8 | 4.77 | <.0001 | 0.05 | 2.3080 | 5.5854 |
| **Lying** | **2** | **Sleep** | **5** | 5.2300 | 0.8275 | 117.8 | 6.32 | <.0001 | 0.05 | 3.5913 | 6.8687 |
| **Lying** | **2** | **Sleep** | **6** | 5.0642 | 0.8275 | 117.8 | 6.12 | <.0001 | 0.05 | 3.4255 | 6.7029 |
| **Lying** | **2** | **Sleep** | **7** | 4.7850 | 0.8275 | 117.8 | 5.78 | <.0001 | 0.05 | 3.1463 | 6.4237 |
| **Lying** | **2** | **Sleep** | **8** | 5.6108 | 0.8275 | 117.8 | 6.78 | <.0001 | 0.05 | 3.9721 | 7.2495 |
| **Lying** | **3** | **Lying** | **4** | 1.1800 | 0.8920 | 49.19 | 1.32 | 0.1920 | 0.05 | -0.6124 | 2.9724 |
| **Lying** | **3** | **Lying** | **5** | 2.0900 | 0.7606 | 71.13 | 2.75 | 0.0076 | 0.05 | 0.5735 | 3.6065 |
| **Lying** | **3** | **Lying** | **6** | 2.1542 | 0.8327 | 59.93 | 2.59 | 0.0121 | 0.05 | 0.4885 | 3.8199 |
| **Lying** | **3** | **Lying** | **7** | 1.7733 | 0.8089 | 69.05 | 2.19 | 0.0317 | 0.05 | 0.1596 | 3.3870 |
| **Lying** | **3** | **Lying** | **8** | 2.5833 | 0.8032 | 73.72 | 3.22 | 0.0019 | 0.05 | 0.9828 | 4.1838 |
| **Lying** | **3** | **Sleep** | **0** | 5.3558 | 0.8275 | 117.8 | 6.47 | <.0001 | 0.05 | 3.7171 | 6.9945 |
| **Lying** | **3** | **Sleep** | **1** | 5.6183 | 0.8275 | 117.8 | 6.79 | <.0001 | 0.05 | 3.9796 | 7.2570 |
| **Lying** | **3** | **Sleep** | **2** | 0.3508 | 0.8275 | 117.8 | 0.42 | 0.6724 | 0.05 | -1.2879 | 1.9895 |
| **Lying** | **3** | **Sleep** | **3** | 1.2808 | 0.8275 | 117.8 | 1.55 | 0.1243 | 0.05 | -0.3579 | 2.9195 |
| **Lying** | **3** | **Sleep** | **4** | 1.1225 | 0.8275 | 117.8 | 1.36 | 0.1775 | 0.05 | -0.5162 | 2.7612 |
| **Lying** | **3** | **Sleep** | **5** | 2.4058 | 0.8275 | 117.8 | 2.91 | 0.0044 | 0.05 | 0.7671 | 4.0445 |
| **Lying** | **3** | **Sleep** | **6** | 2.2400 | 0.8275 | 117.8 | 2.71 | 0.0078 | 0.05 | 0.6013 | 3.8787 |
| **Lying** | **3** | **Sleep** | **7** | 1.9608 | 0.8275 | 117.8 | 2.37 | 0.0194 | 0.05 | 0.3221 | 3.5995 |
| **Lying** | **3** | **Sleep** | **8** | 2.7867 | 0.8275 | 117.8 | 3.37 | 0.0010 | 0.05 | 1.1480 | 4.4254 |
| **Lying** | **4** | **Lying** | **5** | 0.9100 | 0.8920 | 49.19 | 1.02 | 0.3126 | 0.05 | -0.8824 | 2.7024 |
| **Lying** | **4** | **Lying** | **6** | 0.9742 | 0.7606 | 71.13 | 1.28 | 0.2044 | 0.05 | -0.5423 | 2.4907 |
| **Lying** | **4** | **Lying** | **7** | 0.5933 | 0.8327 | 59.93 | 0.71 | 0.4789 | 0.05 | -1.0724 | 2.2590 |
| **Lying** | **4** | **Lying** | **8** | 1.4033 | 0.8089 | 69.05 | 1.73 | 0.0872 | 0.05 | -0.2104 | 3.0170 |
| **Lying** | **4** | **Sleep** | **0** | 4.1758 | 0.8275 | 117.8 | 5.05 | <.0001 | 0.05 | 2.5371 | 5.8145 |
| **Lying** | **4** | **Sleep** | **1** | 4.4383 | 0.8275 | 117.8 | 5.36 | <.0001 | 0.05 | 2.7996 | 6.0770 |
| **Lying** | **4** | **Sleep** | **2** | -0.8292 | 0.8275 | 117.8 | -1.00 | 0.3184 | 0.05 | -2.4679 | 0.8095 |
| **Lying** | **4** | **Sleep** | **3** | 0.1008 | 0.8275 | 117.8 | 0.12 | 0.9032 | 0.05 | -1.5379 | 1.7395 |
| **Lying** | **4** | **Sleep** | **4** | -0.05750 | 0.8275 | 117.8 | -0.07 | 0.9447 | 0.05 | -1.6962 | 1.5812 |
| **Lying** | **4** | **Sleep** | **5** | 1.2258 | 0.8275 | 117.8 | 1.48 | 0.1412 | 0.05 | -0.4129 | 2.8645 |
| **Lying** | **4** | **Sleep** | **6** | 1.0600 | 0.8275 | 117.8 | 1.28 | 0.2027 | 0.05 | -0.5787 | 2.6987 |
| **Lying** | **4** | **Sleep** | **7** | 0.7808 | 0.8275 | 117.8 | 0.94 | 0.3473 | 0.05 | -0.8579 | 2.4195 |
| **Lying** | **4** | **Sleep** | **8** | 1.6067 | 0.8275 | 117.8 | 1.94 | 0.0546 | 0.05 | -0.03203 | 3.2454 |
| **Lying** | **5** | **Lying** | **6** | 0.06417 | 0.8920 | 49.19 | 0.07 | 0.9429 | 0.05 | -1.7282 | 1.8565 |
| **Lying** | **5** | **Lying** | **7** | -0.3167 | 0.7606 | 71.13 | -0.42 | 0.6784 | 0.05 | -1.8332 | 1.1998 |
| **Lying** | **5** | **Lying** | **8** | 0.4933 | 0.8327 | 59.93 | 0.59 | 0.5558 | 0.05 | -1.1724 | 2.1590 |
| **Lying** | **5** | **Sleep** | **0** | 3.2658 | 0.8275 | 117.8 | 3.95 | 0.0001 | 0.05 | 1.6271 | 4.9045 |
| **Lying** | **5** | **Sleep** | **1** | 3.5283 | 0.8275 | 117.8 | 4.26 | <.0001 | 0.05 | 1.8896 | 5.1670 |
| **Lying** | **5** | **Sleep** | **2** | -1.7392 | 0.8275 | 117.8 | -2.10 | 0.0377 | 0.05 | -3.3779 | -0.1005 |
| **Lying** | **5** | **Sleep** | **3** | -0.8092 | 0.8275 | 117.8 | -0.98 | 0.3302 | 0.05 | -2.4479 | 0.8295 |
| **Lying** | **5** | **Sleep** | **4** | -0.9675 | 0.8275 | 117.8 | -1.17 | 0.2447 | 0.05 | -2.6062 | 0.6712 |
| **Lying** | **5** | **Sleep** | **5** | 0.3158 | 0.8275 | 117.8 | 0.38 | 0.7034 | 0.05 | -1.3229 | 1.9545 |
| **Lying** | **5** | **Sleep** | **6** | 0.1500 | 0.8275 | 117.8 | 0.18 | 0.8565 | 0.05 | -1.4887 | 1.7887 |
| **Lying** | **5** | **Sleep** | **7** | -0.1292 | 0.8275 | 117.8 | -0.16 | 0.8762 | 0.05 | -1.7679 | 1.5095 |
| **Lying** | **5** | **Sleep** | **8** | 0.6967 | 0.8275 | 117.8 | 0.84 | 0.4016 | 0.05 | -0.9420 | 2.3354 |
| **Lying** | **6** | **Lying** | **7** | -0.3808 | 0.8920 | 49.19 | -0.43 | 0.6713 | 0.05 | -2.1732 | 1.4115 |
| **Lying** | **6** | **Lying** | **8** | 0.4292 | 0.7606 | 71.13 | 0.56 | 0.5744 | 0.05 | -1.0873 | 1.9457 |
| **Lying** | **6** | **Sleep** | **0** | 3.2017 | 0.8275 | 117.8 | 3.87 | 0.0002 | 0.05 | 1.5630 | 4.8404 |
| **Lying** | **6** | **Sleep** | **1** | 3.4642 | 0.8275 | 117.8 | 4.19 | <.0001 | 0.05 | 1.8255 | 5.1029 |
| **Lying** | **6** | **Sleep** | **2** | -1.8033 | 0.8275 | 117.8 | -2.18 | 0.0313 | 0.05 | -3.4420 | -0.1646 |
| **Lying** | **6** | **Sleep** | **3** | -0.8733 | 0.8275 | 117.8 | -1.06 | 0.2934 | 0.05 | -2.5120 | 0.7654 |
| **Lying** | **6** | **Sleep** | **4** | -1.0317 | 0.8275 | 117.8 | -1.25 | 0.2150 | 0.05 | -2.6704 | 0.6070 |
| **Lying** | **6** | **Sleep** | **5** | 0.2517 | 0.8275 | 117.8 | 0.30 | 0.7616 | 0.05 | -1.3870 | 1.8904 |
| **Lying** | **6** | **Sleep** | **6** | 0.08583 | 0.8275 | 117.8 | 0.10 | 0.9176 | 0.05 | -1.5529 | 1.7245 |
| **Lying** | **6** | **Sleep** | **7** | -0.1933 | 0.8275 | 117.8 | -0.23 | 0.8157 | 0.05 | -1.8320 | 1.4454 |
| **Lying** | **6** | **Sleep** | **8** | 0.6325 | 0.8275 | 117.8 | 0.76 | 0.4462 | 0.05 | -1.0062 | 2.2712 |
| **Lying** | **7** | **Lying** | **8** | 0.8100 | 0.8920 | 49.19 | 0.91 | 0.3683 | 0.05 | -0.9824 | 2.6024 |
| **Lying** | **7** | **Sleep** | **0** | 3.5825 | 0.8275 | 117.8 | 4.33 | <.0001 | 0.05 | 1.9438 | 5.2212 |
| **Lying** | **7** | **Sleep** | **1** | 3.8450 | 0.8275 | 117.8 | 4.65 | <.0001 | 0.05 | 2.2063 | 5.4837 |
| **Lying** | **7** | **Sleep** | **2** | -1.4225 | 0.8275 | 117.8 | -1.72 | 0.0882 | 0.05 | -3.0612 | 0.2162 |
| **Lying** | **7** | **Sleep** | **3** | -0.4925 | 0.8275 | 117.8 | -0.60 | 0.5529 | 0.05 | -2.1312 | 1.1462 |
| **Lying** | **7** | **Sleep** | **4** | -0.6508 | 0.8275 | 117.8 | -0.79 | 0.4331 | 0.05 | -2.2895 | 0.9879 |
| **Lying** | **7** | **Sleep** | **5** | 0.6325 | 0.8275 | 117.8 | 0.76 | 0.4462 | 0.05 | -1.0062 | 2.2712 |
| **Lying** | **7** | **Sleep** | **6** | 0.4667 | 0.8275 | 117.8 | 0.56 | 0.5739 | 0.05 | -1.1720 | 2.1054 |
| **Lying** | **7** | **Sleep** | **7** | 0.1875 | 0.8275 | 117.8 | 0.23 | 0.8211 | 0.05 | -1.4512 | 1.8262 |
| **Lying** | **7** | **Sleep** | **8** | 1.0133 | 0.8275 | 117.8 | 1.22 | 0.2232 | 0.05 | -0.6254 | 2.6520 |
| **Lying** | **8** | **Sleep** | **0** | 2.7725 | 0.8275 | 117.8 | 3.35 | 0.0011 | 0.05 | 1.1338 | 4.4112 |
| **Lying** | **8** | **Sleep** | **1** | 3.0350 | 0.8275 | 117.8 | 3.67 | 0.0004 | 0.05 | 1.3963 | 4.6737 |
| **Lying** | **8** | **Sleep** | **2** | -2.2325 | 0.8275 | 117.8 | -2.70 | 0.0080 | 0.05 | -3.8712 | -0.5938 |
| **Lying** | **8** | **Sleep** | **3** | -1.3025 | 0.8275 | 117.8 | -1.57 | 0.1182 | 0.05 | -2.9412 | 0.3362 |
| **Lying** | **8** | **Sleep** | **4** | -1.4608 | 0.8275 | 117.8 | -1.77 | 0.0801 | 0.05 | -3.0995 | 0.1779 |
| **Lying** | **8** | **Sleep** | **5** | -0.1775 | 0.8275 | 117.8 | -0.21 | 0.8305 | 0.05 | -1.8162 | 1.4612 |
| **Lying** | **8** | **Sleep** | **6** | -0.3433 | 0.8275 | 117.8 | -0.41 | 0.6790 | 0.05 | -1.9820 | 1.2954 |
| **Lying** | **8** | **Sleep** | **7** | -0.6225 | 0.8275 | 117.8 | -0.75 | 0.4534 | 0.05 | -2.2612 | 1.0162 |
| **Lying** | **8** | **Sleep** | **8** | 0.2033 | 0.8275 | 117.8 | 0.25 | 0.8063 | 0.05 | -1.4354 | 1.8420 |
| **Sleep** | **0** | **Sleep** | **1** | 0.2625 | 0.7101 | 83.29 | 0.37 | 0.7126 | 0.05 | -1.1498 | 1.6748 |
| **Sleep** | **0** | **Sleep** | **2** | -5.0050 | 0.7311 | 95.97 | -6.85 | <.0001 | 0.05 | -6.4562 | -3.5538 |
| **Sleep** | **0** | **Sleep** | **3** | -4.0750 | 0.7493 | 88.45 | -5.44 | <.0001 | 0.05 | -5.5639 | -2.5861 |
| **Sleep** | **0** | **Sleep** | **4** | -4.2333 | 0.7650 | 77.09 | -5.53 | <.0001 | 0.05 | -5.7566 | -2.7101 |
| **Sleep** | **0** | **Sleep** | **5** | -2.9500 | 0.7785 | 68.14 | -3.79 | 0.0003 | 0.05 | -4.5035 | -1.3965 |
| **Sleep** | **0** | **Sleep** | **6** | -3.1158 | 0.7902 | 61.96 | -3.94 | 0.0002 | 0.05 | -4.6954 | -1.5363 |
| **Sleep** | **0** | **Sleep** | **7** | -3.3950 | 0.8001 | 57.82 | -4.24 | <.0001 | 0.05 | -4.9967 | -1.7933 |
| **Sleep** | **0** | **Sleep** | **8** | -2.5692 | 0.8085 | 55.03 | -3.18 | 0.0024 | 0.05 | -4.1895 | -0.9488 |
| **Sleep** | **1** | **Sleep** | **2** | -5.2675 | 0.7101 | 83.29 | -7.42 | <.0001 | 0.05 | -6.6798 | -3.8552 |
| **Sleep** | **1** | **Sleep** | **3** | -4.3375 | 0.7311 | 95.97 | -5.93 | <.0001 | 0.05 | -5.7887 | -2.8863 |
| **Sleep** | **1** | **Sleep** | **4** | -4.4958 | 0.7493 | 88.45 | -6.00 | <.0001 | 0.05 | -5.9847 | -3.0069 |
| **Sleep** | **1** | **Sleep** | **5** | -3.2125 | 0.7650 | 77.09 | -4.20 | <.0001 | 0.05 | -4.7358 | -1.6892 |
| **Sleep** | **1** | **Sleep** | **6** | -3.3783 | 0.7785 | 68.14 | -4.34 | <.0001 | 0.05 | -4.9318 | -1.8248 |
| **Sleep** | **1** | **Sleep** | **7** | -3.6575 | 0.7902 | 61.96 | -4.63 | <.0001 | 0.05 | -5.2371 | -2.0779 |
| **Sleep** | **1** | **Sleep** | **8** | -2.8317 | 0.8001 | 57.82 | -3.54 | 0.0008 | 0.05 | -4.4334 | -1.2300 |
| **Sleep** | **2** | **Sleep** | **3** | 0.9300 | 0.7101 | 83.29 | 1.31 | 0.1939 | 0.05 | -0.4823 | 2.3423 |
| **Sleep** | **2** | **Sleep** | **4** | 0.7717 | 0.7311 | 95.97 | 1.06 | 0.2938 | 0.05 | -0.6795 | 2.2228 |
| **Sleep** | **2** | **Sleep** | **5** | 2.0550 | 0.7493 | 88.45 | 2.74 | 0.0074 | 0.05 | 0.5661 | 3.5439 |
| **Sleep** | **2** | **Sleep** | **6** | 1.8892 | 0.7650 | 77.09 | 2.47 | 0.0157 | 0.05 | 0.3659 | 3.4124 |
| **Sleep** | **2** | **Sleep** | **7** | 1.6100 | 0.7785 | 68.14 | 2.07 | 0.0424 | 0.05 | 0.05650 | 3.1635 |
| **Sleep** | **2** | **Sleep** | **8** | 2.4358 | 0.7902 | 61.96 | 3.08 | 0.0031 | 0.05 | 0.8563 | 4.0154 |
| **Sleep** | **3** | **Sleep** | **4** | -0.1583 | 0.7101 | 83.29 | -0.22 | 0.8241 | 0.05 | -1.5706 | 1.2539 |
| **Sleep** | **3** | **Sleep** | **5** | 1.1250 | 0.7311 | 95.97 | 1.54 | 0.1271 | 0.05 | -0.3262 | 2.5762 |
| **Sleep** | **3** | **Sleep** | **6** | 0.9592 | 0.7493 | 88.45 | 1.28 | 0.2038 | 0.05 | -0.5297 | 2.4481 |
| **Sleep** | **3** | **Sleep** | **7** | 0.6800 | 0.7650 | 77.09 | 0.89 | 0.3768 | 0.05 | -0.8433 | 2.2033 |
| **Sleep** | **3** | **Sleep** | **8** | 1.5058 | 0.7785 | 68.14 | 1.93 | 0.0572 | 0.05 | -0.04767 | 3.0593 |
| **Sleep** | **4** | **Sleep** | **5** | 1.2833 | 0.7101 | 83.29 | 1.81 | 0.0743 | 0.05 | -0.1289 | 2.6956 |
| **Sleep** | **4** | **Sleep** | **6** | 1.1175 | 0.7311 | 95.97 | 1.53 | 0.1297 | 0.05 | -0.3337 | 2.5687 |
| **Sleep** | **4** | **Sleep** | **7** | 0.8383 | 0.7493 | 88.45 | 1.12 | 0.2662 | 0.05 | -0.6506 | 2.3272 |
| **Sleep** | **4** | **Sleep** | **8** | 1.6642 | 0.7650 | 77.09 | 2.18 | 0.0327 | 0.05 | 0.1409 | 3.1874 |
| **Sleep** | **5** | **Sleep** | **6** | -0.1658 | 0.7101 | 83.29 | -0.23 | 0.8159 | 0.05 | -1.5781 | 1.2464 |
| **Sleep** | **5** | **Sleep** | **7** | -0.4450 | 0.7311 | 95.97 | -0.61 | 0.5442 | 0.05 | -1.8962 | 1.0062 |
| **Sleep** | **5** | **Sleep** | **8** | 0.3808 | 0.7493 | 88.45 | 0.51 | 0.6125 | 0.05 | -1.1081 | 1.8697 |
| **Sleep** | **6** | **Sleep** | **7** | -0.2792 | 0.7101 | 83.29 | -0.39 | 0.6952 | 0.05 | -1.6914 | 1.1331 |
| **Sleep** | **6** | **Sleep** | **8** | 0.5467 | 0.7311 | 95.97 | 0.75 | 0.4564 | 0.05 | -0.9045 | 1.9978 |
| **Sleep** | **7** | **Sleep** | **8** | 0.8258 | 0.7101 | 83.29 | 1.16 | 0.2482 | 0.05 | -0.5864 | 2.2381 |

# Steps

| **Type III Tests of Fixed Effects** | | | | |
| --- | --- | --- | --- | --- |
| **Effect** | **Num DF** | **Den DF** | **F Value** | **Pr > F** |
| **Period2** | 1 | 197 | 0.08 | 0.7754 |
| **TRT** | 1 | 197 | 0.01 | 0.9416 |
| **day** | 8 | 197 | 15.01 | <.0001 |
| **TRT*day** | 8 | 197 | 3.68 | 0.0005 |

| **TRT Least Squares Means** | | | | | | | | | | | | |
| --- | --- | --- | --- | --- | --- | --- | --- | --- | --- | --- | --- | --- |
| **TRT** | **Estimate** | **Standard Error** | **DF** | **t Value** | **Pr > \|t\|** | **Alpha** | **Lower** | **Upper** | **Mean** | **Standard Error Mean** | **Lower Mean** | **Upper Mean** |
| **Lying** | 1999.92 | 227.23 | 197 | 8.80 | <.0001 | 0.05 | 1551.80 | 2448.03 | 1999.92 | 227.23 | 1551.80 | 2448.03 |
| **Sleep** | 2010.19 | 249.54 | 197 | 8.06 | <.0001 | 0.05 | 1518.09 | 2502.30 | 2010.19 | 249.54 | 1518.09 | 2502.30 |


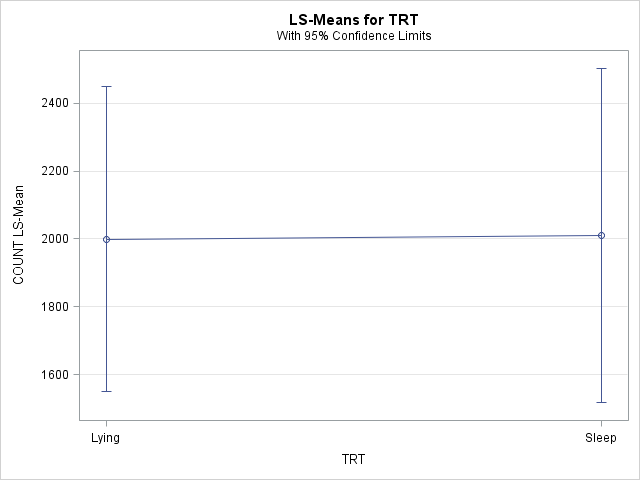


| **Differences of TRT Least Squares Means** | | | | | | | | | |
| --- | --- | --- | --- | --- | --- | --- | --- | --- | --- |
| **TRT** | **_TRT** | **Estimate** | **Standard Error** | **DF** | **t Value** | **Pr > \|t\|** | **Alpha** | **Lower** | **Upper** |
| **Lying** | **Sleep** | -10.2778 | 140.11 | 197 | -0.07 | 0.9416 | 0.05 | -286.59 | 266.03 |

| **day Least Squares Means** | | | | | | | | | | | | |
| --- | --- | --- | --- | --- | --- | --- | --- | --- | --- | --- | --- | --- |
| **day** | **Estimate** | **Standard Error** | **DF** | **t Value** | **Pr > \|t\|** | **Alpha** | **Lower** | **Upper** | **Mean** | **Standard Error Mean** | **Lower Mean** | **Upper Mean** |
| **0** | 2523.04 | 247.40 | 197 | 10.20 | <.0001 | 0.05 | 2035.16 | 3010.93 | 2523.04 | 247.40 | 2035.16 | 3010.93 |
| **1** | 2928.04 | 247.40 | 197 | 11.84 | <.0001 | 0.05 | 2440.16 | 3415.93 | 2928.04 | 247.40 | 2440.16 | 3415.93 |
| **2** | 1814.58 | 247.40 | 197 | 7.33 | <.0001 | 0.05 | 1326.70 | 2302.47 | 1814.58 | 247.40 | 1326.70 | 2302.47 |
| **3** | 1723.17 | 247.40 | 197 | 6.97 | <.0001 | 0.05 | 1235.28 | 2211.05 | 1723.17 | 247.40 | 1235.28 | 2211.05 |
| **4** | 1721.37 | 247.40 | 197 | 6.96 | <.0001 | 0.05 | 1233.49 | 2209.26 | 1721.37 | 247.40 | 1233.49 | 2209.26 |
| **5** | 1968.67 | 247.40 | 197 | 7.96 | <.0001 | 0.05 | 1480.78 | 2456.55 | 1968.67 | 247.40 | 1480.78 | 2456.55 |
| **6** | 1803.96 | 247.40 | 197 | 7.29 | <.0001 | 0.05 | 1316.07 | 2291.84 | 1803.96 | 247.40 | 1316.07 | 2291.84 |
| **7** | 1794.58 | 247.40 | 197 | 7.25 | <.0001 | 0.05 | 1306.70 | 2282.47 | 1794.58 | 247.40 | 1306.70 | 2282.47 |
| **8** | 1768.08 | 247.40 | 197 | 7.15 | <.0001 | 0.05 | 1280.20 | 2255.97 | 1768.08 | 247.40 | 1280.20 | 2255.97 |


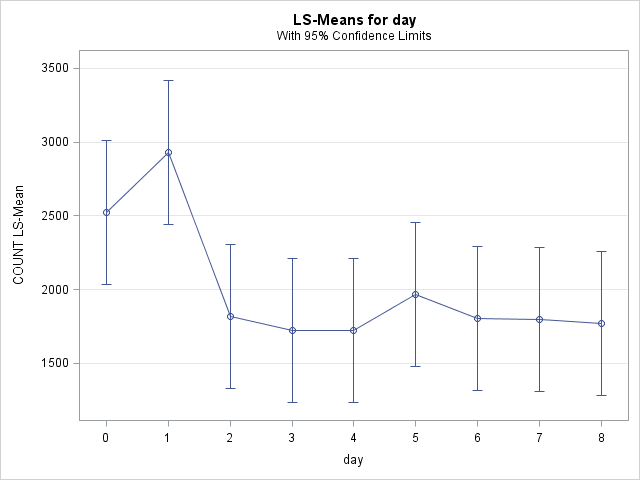


| **Differences of day Least Squares Means** | | | | | | | | | |
| --- | --- | --- | --- | --- | --- | --- | --- | --- | --- |
| **day** | **_day** | **Estimate** | **Standard Error** | **DF** | **t Value** | **Pr > \|t\|** | **Alpha** | **Lower** | **Upper** |
| **0** | **1** | -405.00 | 123.32 | 197 | -3.28 | 0.0012 | 0.05 | -648.19 | -161.81 |
| **0** | **2** | 708.46 | 137.76 | 197 | 5.14 | <.0001 | 0.05 | 436.79 | 980.13 |
| **0** | **3** | 799.88 | 146.15 | 197 | 5.47 | <.0001 | 0.05 | 511.65 | 1088.10 |
| **0** | **4** | 801.67 | 151.48 | 197 | 5.29 | <.0001 | 0.05 | 502.93 | 1100.40 |
| **0** | **5** | 554.38 | 154.76 | 197 | 3.58 | 0.0004 | 0.05 | 249.18 | 859.57 |
| **0** | **6** | 719.08 | 156.78 | 197 | 4.59 | <.0001 | 0.05 | 409.90 | 1028.27 |
| **0** | **7** | 728.46 | 158.03 | 197 | 4.61 | <.0001 | 0.05 | 416.81 | 1040.11 |
| **0** | **8** | 754.96 | 158.80 | 197 | 4.75 | <.0001 | 0.05 | 441.79 | 1068.13 |
| **1** | **2** | 1113.46 | 123.32 | 197 | 9.03 | <.0001 | 0.05 | 870.26 | 1356.65 |
| **1** | **3** | 1204.87 | 137.76 | 197 | 8.75 | <.0001 | 0.05 | 933.20 | 1476.55 |
| **1** | **4** | 1206.67 | 146.15 | 197 | 8.26 | <.0001 | 0.05 | 918.44 | 1494.89 |
| **1** | **5** | 959.37 | 151.48 | 197 | 6.33 | <.0001 | 0.05 | 660.64 | 1258.11 |
| **1** | **6** | 1124.08 | 154.76 | 197 | 7.26 | <.0001 | 0.05 | 818.89 | 1429.27 |
| **1** | **7** | 1133.46 | 156.78 | 197 | 7.23 | <.0001 | 0.05 | 824.27 | 1442.64 |
| **1** | **8** | 1159.96 | 158.03 | 197 | 7.34 | <.0001 | 0.05 | 848.31 | 1471.61 |
| **2** | **3** | 91.4167 | 123.32 | 197 | 0.74 | 0.4594 | 0.05 | -151.78 | 334.61 |
| **2** | **4** | 93.2083 | 137.76 | 197 | 0.68 | 0.4995 | 0.05 | -178.46 | 364.88 |
| **2** | **5** | -154.08 | 146.15 | 197 | -1.05 | 0.2931 | 0.05 | -442.31 | 134.14 |
| **2** | **6** | 10.6250 | 151.48 | 197 | 0.07 | 0.9442 | 0.05 | -288.11 | 309.36 |
| **2** | **7** | 20.0000 | 154.76 | 197 | 0.13 | 0.8973 | 0.05 | -285.19 | 325.19 |
| **2** | **8** | 46.5000 | 156.78 | 197 | 0.30 | 0.7671 | 0.05 | -262.68 | 355.68 |
| **3** | **4** | 1.7917 | 123.32 | 197 | 0.01 | 0.9884 | 0.05 | -241.40 | 244.99 |
| **3** | **5** | -245.50 | 137.76 | 197 | -1.78 | 0.0763 | 0.05 | -517.17 | 26.1726 |
| **3** | **6** | -80.7917 | 146.15 | 197 | -0.55 | 0.5810 | 0.05 | -369.02 | 207.43 |
| **3** | **7** | -71.4167 | 151.48 | 197 | -0.47 | 0.6378 | 0.05 | -370.15 | 227.32 |
| **3** | **8** | -44.9167 | 154.76 | 197 | -0.29 | 0.7719 | 0.05 | -350.11 | 260.27 |
| **4** | **5** | -247.29 | 123.32 | 197 | -2.01 | 0.0463 | 0.05 | -490.49 | -4.0982 |
| **4** | **6** | -82.5833 | 137.76 | 197 | -0.60 | 0.5495 | 0.05 | -354.26 | 189.09 |
| **4** | **7** | -73.2083 | 146.15 | 197 | -0.50 | 0.6170 | 0.05 | -361.43 | 215.02 |
| **4** | **8** | -46.7083 | 151.48 | 197 | -0.31 | 0.7581 | 0.05 | -345.44 | 252.02 |
| **5** | **6** | 164.71 | 123.32 | 197 | 1.34 | 0.1832 | 0.05 | -78.4852 | 407.90 |
| **5** | **7** | 174.08 | 137.76 | 197 | 1.26 | 0.2078 | 0.05 | -97.5893 | 445.76 |
| **5** | **8** | 200.58 | 146.15 | 197 | 1.37 | 0.1715 | 0.05 | -87.6401 | 488.81 |
| **6** | **7** | 9.3750 | 123.32 | 197 | 0.08 | 0.9395 | 0.05 | -233.82 | 252.57 |
| **6** | **8** | 35.8750 | 137.76 | 197 | 0.26 | 0.7948 | 0.05 | -235.80 | 307.55 |
| **7** | **8** | 26.5000 | 123.32 | 197 | 0.21 | 0.8301 | 0.05 | -216.69 | 269.69 |

| **TRT*day Least Squares Means** | | | | | | | | | | | | | |
| --- | --- | --- | --- | --- | --- | --- | --- | --- | --- | --- | --- | --- | --- |
| **TRT** | **day** | **Estimate** | **Standard Error** | **DF** | **t Value** | **Pr > \|t\|** | **Alpha** | **Lower** | **Upper** | **Mean** | **Standard Error Mean** | **Lower Mean** | **Upper Mean** |
| **Lying** | **0** | 2422.75 | 260.69 | 197 | 9.29 | <.0001 | 0.05 | 1908.64 | 2936.86 | 2422.75 | 260.69 | 1908.64 | 2936.86 |
| **Lying** | **1** | 3318.25 | 260.69 | 197 | 12.73 | <.0001 | 0.05 | 2804.14 | 3832.36 | 3318.25 | 260.69 | 2804.14 | 3832.36 |
| **Lying** | **2** | 1618.83 | 260.69 | 197 | 6.21 | <.0001 | 0.05 | 1104.72 | 2132.94 | 1618.83 | 260.69 | 1104.72 | 2132.94 |
| **Lying** | **3** | 1618.00 | 260.69 | 197 | 6.21 | <.0001 | 0.05 | 1103.89 | 2132.11 | 1618.00 | 260.69 | 1103.89 | 2132.11 |
| **Lying** | **4** | 1686.00 | 260.69 | 197 | 6.47 | <.0001 | 0.05 | 1171.89 | 2200.11 | 1686.00 | 260.69 | 1171.89 | 2200.11 |
| **Lying** | **5** | 2012.83 | 260.69 | 197 | 7.72 | <.0001 | 0.05 | 1498.72 | 2526.94 | 2012.83 | 260.69 | 1498.72 | 2526.94 |
| **Lying** | **6** | 1788.83 | 260.69 | 197 | 6.86 | <.0001 | 0.05 | 1274.72 | 2302.94 | 1788.83 | 260.69 | 1274.72 | 2302.94 |
| **Lying** | **7** | 1805.17 | 260.69 | 197 | 6.92 | <.0001 | 0.05 | 1291.06 | 2319.28 | 1805.17 | 260.69 | 1291.06 | 2319.28 |
| **Lying** | **8** | 1728.58 | 260.69 | 197 | 6.63 | <.0001 | 0.05 | 1214.47 | 2242.69 | 1728.58 | 260.69 | 1214.47 | 2242.69 |
| **Sleep** | **0** | 2623.33 | 287.38 | 197 | 9.13 | <.0001 | 0.05 | 2056.60 | 3190.07 | 2623.33 | 287.38 | 2056.60 | 3190.07 |
| **Sleep** | **1** | 2537.83 | 287.38 | 197 | 8.83 | <.0001 | 0.05 | 1971.10 | 3104.57 | 2537.83 | 287.38 | 1971.10 | 3104.57 |
| **Sleep** | **2** | 2010.33 | 287.38 | 197 | 7.00 | <.0001 | 0.05 | 1443.60 | 2577.07 | 2010.33 | 287.38 | 1443.60 | 2577.07 |
| **Sleep** | **3** | 1828.33 | 287.38 | 197 | 6.36 | <.0001 | 0.05 | 1261.60 | 2395.07 | 1828.33 | 287.38 | 1261.60 | 2395.07 |
| **Sleep** | **4** | 1756.75 | 287.38 | 197 | 6.11 | <.0001 | 0.05 | 1190.01 | 2323.49 | 1756.75 | 287.38 | 1190.01 | 2323.49 |
| **Sleep** | **5** | 1924.50 | 287.38 | 197 | 6.70 | <.0001 | 0.05 | 1357.76 | 2491.24 | 1924.50 | 287.38 | 1357.76 | 2491.24 |
| **Sleep** | **6** | 1819.08 | 287.38 | 197 | 6.33 | <.0001 | 0.05 | 1252.35 | 2385.82 | 1819.08 | 287.38 | 1252.35 | 2385.82 |
| **Sleep** | **7** | 1784.00 | 287.38 | 197 | 6.21 | <.0001 | 0.05 | 1217.26 | 2350.74 | 1784.00 | 287.38 | 1217.26 | 2350.74 |
| **Sleep** | **8** | 1807.58 | 287.38 | 197 | 6.29 | <.0001 | 0.05 | 1240.85 | 2374.32 | 1807.58 | 287.38 | 1240.85 | 2374.32 |


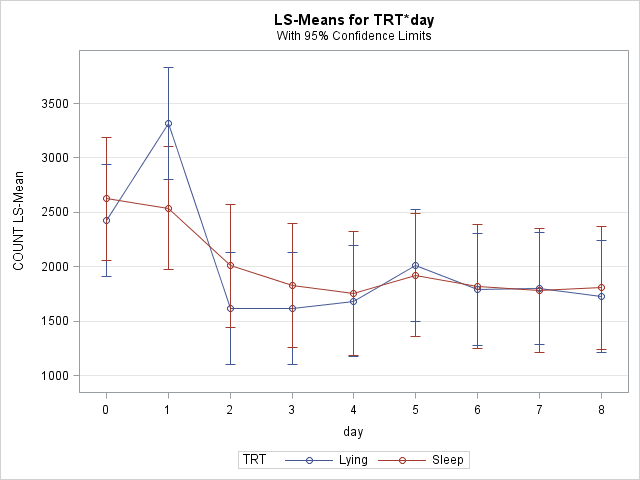


| **Differences of TRT*day Least Squares Means** | | | | | | | | | | | |
| --- | --- | --- | --- | --- | --- | --- | --- | --- | --- | --- | --- |
| **TRT** | **day** | **_TRT** | **_day** | **Estimate** | **Standard Error** | **DF** | **t Value** | **Pr > \|t\|** | **Alpha** | **Lower** | **Upper** |
| **Lying** | **0** | **Lying** | **1** | -895.50 | 192.78 | 197 | -4.65 | <.0001 | 0.05 | -1275.67 | -515.33 |
| **Lying** | **0** | **Lying** | **2** | 803.92 | 192.04 | 197 | 4.19 | <.0001 | 0.05 | 425.19 | 1182.64 |
| **Lying** | **0** | **Lying** | **3** | 804.75 | 191.09 | 197 | 4.21 | <.0001 | 0.05 | 427.90 | 1181.60 |
| **Lying** | **0** | **Lying** | **4** | 736.75 | 191.14 | 197 | 3.85 | 0.0002 | 0.05 | 359.80 | 1113.70 |
| **Lying** | **0** | **Lying** | **5** | 409.92 | 191.14 | 197 | 2.14 | 0.0332 | 0.05 | 32.9745 | 786.86 |
| **Lying** | **0** | **Lying** | **6** | 633.92 | 191.14 | 197 | 3.32 | 0.0011 | 0.05 | 256.97 | 1010.86 |
| **Lying** | **0** | **Lying** | **7** | 617.58 | 191.14 | 197 | 3.23 | 0.0014 | 0.05 | 240.64 | 994.53 |
| **Lying** | **0** | **Lying** | **8** | 694.17 | 191.14 | 197 | 3.63 | 0.0004 | 0.05 | 317.22 | 1071.11 |
| **Lying** | **0** | **Sleep** | **0** | -200.58 | 237.23 | 197 | -0.85 | 0.3988 | 0.05 | -668.42 | 267.25 |
| **Lying** | **0** | **Sleep** | **1** | -115.08 | 237.23 | 197 | -0.49 | 0.6281 | 0.05 | -582.92 | 352.75 |
| **Lying** | **0** | **Sleep** | **2** | 412.42 | 237.23 | 197 | 1.74 | 0.0837 | 0.05 | -55.4184 | 880.25 |
| **Lying** | **0** | **Sleep** | **3** | 594.42 | 237.23 | 197 | 2.51 | 0.0130 | 0.05 | 126.58 | 1062.25 |
| **Lying** | **0** | **Sleep** | **4** | 666.00 | 237.23 | 197 | 2.81 | 0.0055 | 0.05 | 198.16 | 1133.84 |
| **Lying** | **0** | **Sleep** | **5** | 498.25 | 237.23 | 197 | 2.10 | 0.0370 | 0.05 | 30.4149 | 966.09 |
| **Lying** | **0** | **Sleep** | **6** | 603.67 | 237.23 | 197 | 2.54 | 0.0117 | 0.05 | 135.83 | 1071.50 |
| **Lying** | **0** | **Sleep** | **7** | 638.75 | 237.23 | 197 | 2.69 | 0.0077 | 0.05 | 170.91 | 1106.59 |
| **Lying** | **0** | **Sleep** | **8** | 615.17 | 237.23 | 197 | 2.59 | 0.0102 | 0.05 | 147.33 | 1083.00 |
| **Lying** | **1** | **Lying** | **2** | 1699.42 | 192.78 | 197 | 8.82 | <.0001 | 0.05 | 1319.24 | 2079.59 |
| **Lying** | **1** | **Lying** | **3** | 1700.25 | 192.04 | 197 | 8.85 | <.0001 | 0.05 | 1321.52 | 2078.98 |
| **Lying** | **1** | **Lying** | **4** | 1632.25 | 191.09 | 197 | 8.54 | <.0001 | 0.05 | 1255.40 | 2009.10 |
| **Lying** | **1** | **Lying** | **5** | 1305.42 | 191.14 | 197 | 6.83 | <.0001 | 0.05 | 928.47 | 1682.36 |
| **Lying** | **1** | **Lying** | **6** | 1529.42 | 191.14 | 197 | 8.00 | <.0001 | 0.05 | 1152.47 | 1906.36 |
| **Lying** | **1** | **Lying** | **7** | 1513.08 | 191.14 | 197 | 7.92 | <.0001 | 0.05 | 1136.14 | 1890.03 |
| **Lying** | **1** | **Lying** | **8** | 1589.67 | 191.14 | 197 | 8.32 | <.0001 | 0.05 | 1212.72 | 1966.61 |
| **Lying** | **1** | **Sleep** | **0** | 694.92 | 237.23 | 197 | 2.93 | 0.0038 | 0.05 | 227.08 | 1162.75 |
| **Lying** | **1** | **Sleep** | **1** | 780.42 | 237.23 | 197 | 3.29 | 0.0012 | 0.05 | 312.58 | 1248.25 |
| **Lying** | **1** | **Sleep** | **2** | 1307.92 | 237.23 | 197 | 5.51 | <.0001 | 0.05 | 840.08 | 1775.75 |
| **Lying** | **1** | **Sleep** | **3** | 1489.92 | 237.23 | 197 | 6.28 | <.0001 | 0.05 | 1022.08 | 1957.75 |
| **Lying** | **1** | **Sleep** | **4** | 1561.50 | 237.23 | 197 | 6.58 | <.0001 | 0.05 | 1093.66 | 2029.34 |
| **Lying** | **1** | **Sleep** | **5** | 1393.75 | 237.23 | 197 | 5.88 | <.0001 | 0.05 | 925.91 | 1861.59 |
| **Lying** | **1** | **Sleep** | **6** | 1499.17 | 237.23 | 197 | 6.32 | <.0001 | 0.05 | 1031.33 | 1967.00 |
| **Lying** | **1** | **Sleep** | **7** | 1534.25 | 237.23 | 197 | 6.47 | <.0001 | 0.05 | 1066.41 | 2002.09 |
| **Lying** | **1** | **Sleep** | **8** | 1510.67 | 237.23 | 197 | 6.37 | <.0001 | 0.05 | 1042.83 | 1978.50 |
| **Lying** | **2** | **Lying** | **3** | 0.8333 | 192.78 | 197 | 0.00 | 0.9966 | 0.05 | -379.34 | 381.01 |
| **Lying** | **2** | **Lying** | **4** | -67.1667 | 192.04 | 197 | -0.35 | 0.7269 | 0.05 | -445.89 | 311.56 |
| **Lying** | **2** | **Lying** | **5** | -394.00 | 191.09 | 197 | -2.06 | 0.0405 | 0.05 | -770.85 | -17.1520 |
| **Lying** | **2** | **Lying** | **6** | -170.00 | 191.14 | 197 | -0.89 | 0.3749 | 0.05 | -546.95 | 206.95 |
| **Lying** | **2** | **Lying** | **7** | -186.33 | 191.14 | 197 | -0.97 | 0.3308 | 0.05 | -563.28 | 190.61 |
| **Lying** | **2** | **Lying** | **8** | -109.75 | 191.14 | 197 | -0.57 | 0.5665 | 0.05 | -486.69 | 267.19 |
| **Lying** | **2** | **Sleep** | **0** | -1004.50 | 237.23 | 197 | -4.23 | <.0001 | 0.05 | -1472.34 | -536.66 |
| **Lying** | **2** | **Sleep** | **1** | -919.00 | 237.23 | 197 | -3.87 | 0.0001 | 0.05 | -1386.84 | -451.16 |
| **Lying** | **2** | **Sleep** | **2** | -391.50 | 237.23 | 197 | -1.65 | 0.1005 | 0.05 | -859.34 | 76.3351 |
| **Lying** | **2** | **Sleep** | **3** | -209.50 | 237.23 | 197 | -0.88 | 0.3783 | 0.05 | -677.34 | 258.34 |
| **Lying** | **2** | **Sleep** | **4** | -137.92 | 237.23 | 197 | -0.58 | 0.5617 | 0.05 | -605.75 | 329.92 |
| **Lying** | **2** | **Sleep** | **5** | -305.67 | 237.23 | 197 | -1.29 | 0.1991 | 0.05 | -773.50 | 162.17 |
| **Lying** | **2** | **Sleep** | **6** | -200.25 | 237.23 | 197 | -0.84 | 0.3996 | 0.05 | -668.09 | 267.59 |
| **Lying** | **2** | **Sleep** | **7** | -165.17 | 237.23 | 197 | -0.70 | 0.4871 | 0.05 | -633.00 | 302.67 |
| **Lying** | **2** | **Sleep** | **8** | -188.75 | 237.23 | 197 | -0.80 | 0.4272 | 0.05 | -656.59 | 279.09 |
| **Lying** | **3** | **Lying** | **4** | -68.0000 | 192.78 | 197 | -0.35 | 0.7247 | 0.05 | -448.17 | 312.17 |
| **Lying** | **3** | **Lying** | **5** | -394.83 | 192.04 | 197 | -2.06 | 0.0411 | 0.05 | -773.56 | -16.1070 |
| **Lying** | **3** | **Lying** | **6** | -170.83 | 191.09 | 197 | -0.89 | 0.3724 | 0.05 | -547.68 | 206.01 |
| **Lying** | **3** | **Lying** | **7** | -187.17 | 191.14 | 197 | -0.98 | 0.3287 | 0.05 | -564.11 | 189.78 |
| **Lying** | **3** | **Lying** | **8** | -110.58 | 191.14 | 197 | -0.58 | 0.5636 | 0.05 | -487.53 | 266.36 |
| **Lying** | **3** | **Sleep** | **0** | -1005.33 | 237.23 | 197 | -4.24 | <.0001 | 0.05 | -1473.17 | -537.50 |
| **Lying** | **3** | **Sleep** | **1** | -919.83 | 237.23 | 197 | -3.88 | 0.0001 | 0.05 | -1387.67 | -452.00 |
| **Lying** | **3** | **Sleep** | **2** | -392.33 | 237.23 | 197 | -1.65 | 0.0998 | 0.05 | -860.17 | 75.5018 |
| **Lying** | **3** | **Sleep** | **3** | -210.33 | 237.23 | 197 | -0.89 | 0.3764 | 0.05 | -678.17 | 257.50 |
| **Lying** | **3** | **Sleep** | **4** | -138.75 | 237.23 | 197 | -0.58 | 0.5593 | 0.05 | -606.59 | 329.09 |
| **Lying** | **3** | **Sleep** | **5** | -306.50 | 237.23 | 197 | -1.29 | 0.1979 | 0.05 | -774.34 | 161.34 |
| **Lying** | **3** | **Sleep** | **6** | -201.08 | 237.23 | 197 | -0.85 | 0.3977 | 0.05 | -668.92 | 266.75 |
| **Lying** | **3** | **Sleep** | **7** | -166.00 | 237.23 | 197 | -0.70 | 0.4849 | 0.05 | -633.84 | 301.84 |
| **Lying** | **3** | **Sleep** | **8** | -189.58 | 237.23 | 197 | -0.80 | 0.4252 | 0.05 | -657.42 | 278.25 |
| **Lying** | **4** | **Lying** | **5** | -326.83 | 192.78 | 197 | -1.70 | 0.0916 | 0.05 | -707.01 | 53.3408 |
| **Lying** | **4** | **Lying** | **6** | -102.83 | 192.04 | 197 | -0.54 | 0.5929 | 0.05 | -481.56 | 275.89 |
| **Lying** | **4** | **Lying** | **7** | -119.17 | 191.09 | 197 | -0.62 | 0.5336 | 0.05 | -496.01 | 257.68 |
| **Lying** | **4** | **Lying** | **8** | -42.5833 | 191.14 | 197 | -0.22 | 0.8239 | 0.05 | -419.53 | 334.36 |
| **Lying** | **4** | **Sleep** | **0** | -937.33 | 237.23 | 197 | -3.95 | 0.0001 | 0.05 | -1405.17 | -469.50 |
| **Lying** | **4** | **Sleep** | **1** | -851.83 | 237.23 | 197 | -3.59 | 0.0004 | 0.05 | -1319.67 | -384.00 |
| **Lying** | **4** | **Sleep** | **2** | -324.33 | 237.23 | 197 | -1.37 | 0.1731 | 0.05 | -792.17 | 143.50 |
| **Lying** | **4** | **Sleep** | **3** | -142.33 | 237.23 | 197 | -0.60 | 0.5492 | 0.05 | -610.17 | 325.50 |
| **Lying** | **4** | **Sleep** | **4** | -70.7500 | 237.23 | 197 | -0.30 | 0.7658 | 0.05 | -538.59 | 397.09 |
| **Lying** | **4** | **Sleep** | **5** | -238.50 | 237.23 | 197 | -1.01 | 0.3160 | 0.05 | -706.34 | 229.34 |
| **Lying** | **4** | **Sleep** | **6** | -133.08 | 237.23 | 197 | -0.56 | 0.5754 | 0.05 | -600.92 | 334.75 |
| **Lying** | **4** | **Sleep** | **7** | -98.0000 | 237.23 | 197 | -0.41 | 0.6800 | 0.05 | -565.84 | 369.84 |
| **Lying** | **4** | **Sleep** | **8** | -121.58 | 237.23 | 197 | -0.51 | 0.6089 | 0.05 | -589.42 | 346.25 |
| **Lying** | **5** | **Lying** | **6** | 224.00 | 192.78 | 197 | 1.16 | 0.2467 | 0.05 | -156.17 | 604.17 |
| **Lying** | **5** | **Lying** | **7** | 207.67 | 192.04 | 197 | 1.08 | 0.2809 | 0.05 | -171.06 | 586.39 |
| **Lying** | **5** | **Lying** | **8** | 284.25 | 191.09 | 197 | 1.49 | 0.1385 | 0.05 | -92.5980 | 661.10 |
| **Lying** | **5** | **Sleep** | **0** | -610.50 | 237.23 | 197 | -2.57 | 0.0108 | 0.05 | -1078.34 | -142.66 |
| **Lying** | **5** | **Sleep** | **1** | -525.00 | 237.23 | 197 | -2.21 | 0.0280 | 0.05 | -992.84 | -57.1649 |
| **Lying** | **5** | **Sleep** | **2** | 2.5000 | 237.23 | 197 | 0.01 | 0.9916 | 0.05 | -465.34 | 470.34 |
| **Lying** | **5** | **Sleep** | **3** | 184.50 | 237.23 | 197 | 0.78 | 0.4377 | 0.05 | -283.34 | 652.34 |
| **Lying** | **5** | **Sleep** | **4** | 256.08 | 237.23 | 197 | 1.08 | 0.2817 | 0.05 | -211.75 | 723.92 |
| **Lying** | **5** | **Sleep** | **5** | 88.3333 | 237.23 | 197 | 0.37 | 0.7100 | 0.05 | -379.50 | 556.17 |
| **Lying** | **5** | **Sleep** | **6** | 193.75 | 237.23 | 197 | 0.82 | 0.4151 | 0.05 | -274.09 | 661.59 |
| **Lying** | **5** | **Sleep** | **7** | 228.83 | 237.23 | 197 | 0.96 | 0.3359 | 0.05 | -239.00 | 696.67 |
| **Lying** | **5** | **Sleep** | **8** | 205.25 | 237.23 | 197 | 0.87 | 0.3880 | 0.05 | -262.59 | 673.09 |
| **Lying** | **6** | **Lying** | **7** | -16.3333 | 192.78 | 197 | -0.08 | 0.9326 | 0.05 | -396.51 | 363.84 |
| **Lying** | **6** | **Lying** | **8** | 60.2500 | 192.04 | 197 | 0.31 | 0.7541 | 0.05 | -318.48 | 438.98 |
| **Lying** | **6** | **Sleep** | **0** | -834.50 | 237.23 | 197 | -3.52 | 0.0005 | 0.05 | -1302.34 | -366.66 |
| **Lying** | **6** | **Sleep** | **1** | -749.00 | 237.23 | 197 | -3.16 | 0.0018 | 0.05 | -1216.84 | -281.16 |
| **Lying** | **6** | **Sleep** | **2** | -221.50 | 237.23 | 197 | -0.93 | 0.3516 | 0.05 | -689.34 | 246.34 |
| **Lying** | **6** | **Sleep** | **3** | -39.5000 | 237.23 | 197 | -0.17 | 0.8679 | 0.05 | -507.34 | 428.34 |
| **Lying** | **6** | **Sleep** | **4** | 32.0833 | 237.23 | 197 | 0.14 | 0.8926 | 0.05 | -435.75 | 499.92 |
| **Lying** | **6** | **Sleep** | **5** | -135.67 | 237.23 | 197 | -0.57 | 0.5681 | 0.05 | -603.50 | 332.17 |
| **Lying** | **6** | **Sleep** | **6** | -30.2500 | 237.23 | 197 | -0.13 | 0.8987 | 0.05 | -498.09 | 437.59 |
| **Lying** | **6** | **Sleep** | **7** | 4.8333 | 237.23 | 197 | 0.02 | 0.9838 | 0.05 | -463.00 | 472.67 |
| **Lying** | **6** | **Sleep** | **8** | -18.7500 | 237.23 | 197 | -0.08 | 0.9371 | 0.05 | -486.59 | 449.09 |
| **Lying** | **7** | **Lying** | **8** | 76.5833 | 192.78 | 197 | 0.40 | 0.6916 | 0.05 | -303.59 | 456.76 |
| **Lying** | **7** | **Sleep** | **0** | -818.17 | 237.23 | 197 | -3.45 | 0.0007 | 0.05 | -1286.00 | -350.33 |
| **Lying** | **7** | **Sleep** | **1** | -732.67 | 237.23 | 197 | -3.09 | 0.0023 | 0.05 | -1200.50 | -264.83 |
| **Lying** | **7** | **Sleep** | **2** | -205.17 | 237.23 | 197 | -0.86 | 0.3882 | 0.05 | -673.00 | 262.67 |
| **Lying** | **7** | **Sleep** | **3** | -23.1667 | 237.23 | 197 | -0.10 | 0.9223 | 0.05 | -491.00 | 444.67 |
| **Lying** | **7** | **Sleep** | **4** | 48.4167 | 237.23 | 197 | 0.20 | 0.8385 | 0.05 | -419.42 | 516.25 |
| **Lying** | **7** | **Sleep** | **5** | -119.33 | 237.23 | 197 | -0.50 | 0.6155 | 0.05 | -587.17 | 348.50 |
| **Lying** | **7** | **Sleep** | **6** | -13.9167 | 237.23 | 197 | -0.06 | 0.9533 | 0.05 | -481.75 | 453.92 |
| **Lying** | **7** | **Sleep** | **7** | 21.1667 | 237.23 | 197 | 0.09 | 0.9290 | 0.05 | -446.67 | 489.00 |
| **Lying** | **7** | **Sleep** | **8** | -2.4167 | 237.23 | 197 | -0.01 | 0.9919 | 0.05 | -470.25 | 465.42 |
| **Lying** | **8** | **Sleep** | **0** | -894.75 | 237.23 | 197 | -3.77 | 0.0002 | 0.05 | -1362.59 | -426.91 |
| **Lying** | **8** | **Sleep** | **1** | -809.25 | 237.23 | 197 | -3.41 | 0.0008 | 0.05 | -1277.09 | -341.41 |
| **Lying** | **8** | **Sleep** | **2** | -281.75 | 237.23 | 197 | -1.19 | 0.2364 | 0.05 | -749.59 | 186.09 |
| **Lying** | **8** | **Sleep** | **3** | -99.7500 | 237.23 | 197 | -0.42 | 0.6746 | 0.05 | -567.59 | 368.09 |
| **Lying** | **8** | **Sleep** | **4** | -28.1667 | 237.23 | 197 | -0.12 | 0.9056 | 0.05 | -496.00 | 439.67 |
| **Lying** | **8** | **Sleep** | **5** | -195.92 | 237.23 | 197 | -0.83 | 0.4099 | 0.05 | -663.75 | 271.92 |
| **Lying** | **8** | **Sleep** | **6** | -90.5000 | 237.23 | 197 | -0.38 | 0.7033 | 0.05 | -558.34 | 377.34 |
| **Lying** | **8** | **Sleep** | **7** | -55.4167 | 237.23 | 197 | -0.23 | 0.8155 | 0.05 | -523.25 | 412.42 |
| **Lying** | **8** | **Sleep** | **8** | -79.0000 | 237.23 | 197 | -0.33 | 0.7395 | 0.05 | -546.84 | 388.84 |
| **Sleep** | **0** | **Sleep** | **1** | 85.5000 | 153.84 | 80.17 | 0.56 | 0.5799 | 0.05 | -220.64 | 391.64 |
| **Sleep** | **0** | **Sleep** | **2** | 613.00 | 197.56 | 131.5 | 3.10 | 0.0023 | 0.05 | 222.20 | 1003.80 |
| **Sleep** | **0** | **Sleep** | **3** | 795.00 | 221.19 | 197 | 3.59 | 0.0004 | 0.05 | 358.79 | 1231.21 |
| **Sleep** | **0** | **Sleep** | **4** | 866.58 | 235.06 | 197 | 3.69 | 0.0003 | 0.05 | 403.04 | 1330.13 |
| **Sleep** | **0** | **Sleep** | **5** | 698.83 | 243.44 | 197 | 2.87 | 0.0045 | 0.05 | 218.75 | 1178.91 |
| **Sleep** | **0** | **Sleep** | **6** | 804.25 | 248.57 | 197 | 3.24 | 0.0014 | 0.05 | 314.05 | 1294.45 |
| **Sleep** | **0** | **Sleep** | **7** | 839.33 | 251.72 | 197 | 3.33 | 0.0010 | 0.05 | 342.92 | 1335.74 |
| **Sleep** | **0** | **Sleep** | **8** | 815.75 | 253.65 | 197 | 3.22 | 0.0015 | 0.05 | 315.54 | 1315.96 |
| **Sleep** | **1** | **Sleep** | **2** | 527.50 | 153.84 | 80.17 | 3.43 | 0.0010 | 0.05 | 221.36 | 833.64 |
| **Sleep** | **1** | **Sleep** | **3** | 709.50 | 197.56 | 131.5 | 3.59 | 0.0005 | 0.05 | 318.70 | 1100.30 |
| **Sleep** | **1** | **Sleep** | **4** | 781.08 | 221.19 | 197 | 3.53 | 0.0005 | 0.05 | 344.88 | 1217.29 |
| **Sleep** | **1** | **Sleep** | **5** | 613.33 | 235.06 | 197 | 2.61 | 0.0098 | 0.05 | 149.79 | 1076.88 |
| **Sleep** | **1** | **Sleep** | **6** | 718.75 | 243.44 | 197 | 2.95 | 0.0035 | 0.05 | 238.67 | 1198.83 |
| **Sleep** | **1** | **Sleep** | **7** | 753.83 | 248.57 | 197 | 3.03 | 0.0028 | 0.05 | 263.63 | 1244.03 |
| **Sleep** | **1** | **Sleep** | **8** | 730.25 | 251.72 | 197 | 2.90 | 0.0041 | 0.05 | 233.84 | 1226.66 |
| **Sleep** | **2** | **Sleep** | **3** | 182.00 | 153.84 | 80.17 | 1.18 | 0.2403 | 0.05 | -124.14 | 488.14 |
| **Sleep** | **2** | **Sleep** | **4** | 253.58 | 197.56 | 131.5 | 1.28 | 0.2015 | 0.05 | -137.22 | 644.39 |
| **Sleep** | **2** | **Sleep** | **5** | 85.8333 | 221.19 | 197 | 0.39 | 0.6984 | 0.05 | -350.37 | 522.04 |
| **Sleep** | **2** | **Sleep** | **6** | 191.25 | 235.06 | 197 | 0.81 | 0.4168 | 0.05 | -272.30 | 654.80 |
| **Sleep** | **2** | **Sleep** | **7** | 226.33 | 243.44 | 197 | 0.93 | 0.3536 | 0.05 | -253.75 | 706.41 |
| **Sleep** | **2** | **Sleep** | **8** | 202.75 | 248.57 | 197 | 0.82 | 0.4157 | 0.05 | -287.45 | 692.95 |
| **Sleep** | **3** | **Sleep** | **4** | 71.5833 | 153.84 | 80.17 | 0.47 | 0.6430 | 0.05 | -234.55 | 377.72 |
| **Sleep** | **3** | **Sleep** | **5** | -96.1667 | 197.56 | 131.5 | -0.49 | 0.6272 | 0.05 | -486.97 | 294.64 |
| **Sleep** | **3** | **Sleep** | **6** | 9.2500 | 221.19 | 197 | 0.04 | 0.9667 | 0.05 | -426.96 | 445.46 |
| **Sleep** | **3** | **Sleep** | **7** | 44.3333 | 235.06 | 197 | 0.19 | 0.8506 | 0.05 | -419.21 | 507.88 |
| **Sleep** | **3** | **Sleep** | **8** | 20.7500 | 243.44 | 197 | 0.09 | 0.9322 | 0.05 | -459.33 | 500.83 |
| **Sleep** | **4** | **Sleep** | **5** | -167.75 | 153.84 | 80.17 | -1.09 | 0.2788 | 0.05 | -473.89 | 138.39 |
| **Sleep** | **4** | **Sleep** | **6** | -62.3333 | 197.56 | 131.5 | -0.32 | 0.7529 | 0.05 | -453.14 | 328.47 |
| **Sleep** | **4** | **Sleep** | **7** | -27.2500 | 221.19 | 197 | -0.12 | 0.9021 | 0.05 | -463.46 | 408.96 |
| **Sleep** | **4** | **Sleep** | **8** | -50.8333 | 235.06 | 197 | -0.22 | 0.8290 | 0.05 | -514.38 | 412.71 |
| **Sleep** | **5** | **Sleep** | **6** | 105.42 | 153.84 | 80.17 | 0.69 | 0.4952 | 0.05 | -200.72 | 411.55 |
| **Sleep** | **5** | **Sleep** | **7** | 140.50 | 197.56 | 131.5 | 0.71 | 0.4782 | 0.05 | -250.30 | 531.30 |
| **Sleep** | **5** | **Sleep** | **8** | 116.92 | 221.19 | 197 | 0.53 | 0.5977 | 0.05 | -319.29 | 553.12 |
| **Sleep** | **6** | **Sleep** | **7** | 35.0833 | 153.84 | 80.17 | 0.23 | 0.8202 | 0.05 | -271.05 | 341.22 |
| **Sleep** | **6** | **Sleep** | **8** | 11.5000 | 197.56 | 131.5 | 0.06 | 0.9537 | 0.05 | -379.30 | 402.30 |
| **Sleep** | **7** | **Sleep** | **8** | -23.5833 | 153.84 | 80.17 | -0.15 | 0.8785 | 0.05 | -329.72 | 282.55 |

# Somatic cell

Effect=Day Method=LSD(P<.05) Set=3

| **Obs** | **Trt** | **Day** | **Period** | **Estimate** | **Standard Error** | **Mean** | **Standard Error of Mean** | **UnTrans_Mean** | **UnTrans_Stderr** | **Letter Group** | **BT_Mean** | **BT_StdErr** |
| --- | --- | --- | --- | --- | --- | --- | --- | --- | --- | --- | --- | --- |
| **5** |  | 0 | _ | 10.9948 | 0.2862 | 10.9948 | 0.2862 | 88579 | 24915 | A | 59562.44 | 17048.07 |
| **6** |  | 1 | _ | 10.6563 | 0.2862 | 10.6563 | 0.2862 | 71315 | 24915 | B | 42459.18 | 12152.74 |
| **7** |  | 2 | _ | 11.0755 | 0.2873 | 11.0755 | 0.2873 | 106088 | 25003 | A | 64568.41 | 18549.43 |
| **8** |  | 3 | _ | 10.8211 | 0.2885 | 10.8211 | 0.2885 | 89561 | 25101 | AB | 50066.01 | 14444.55 |

# Fat

Effect=Day Method=LSD(P<.05) Set=3

| **Obs** | **Trt** | **Day** | **Period** | **Estimate** | **Standard Error** | **Mean** | **Standard Error of Mean** | **UnTrans_Mean** | **UnTrans_Stderr** | **Letter Group** | **BT_Mean** | **BT_StdErr** |
| --- | --- | --- | --- | --- | --- | --- | --- | --- | --- | --- | --- | --- |
| **5** |  | 0 | _ | 8.1229 | 0.05992 | 8.1229 | 0.05992 | 3429.92 | 233.79 | A | 3370.71 | 201.963 |
| **6** |  | 1 | _ | 7.9965 | 0.05992 | 7.9965 | 0.05992 | 3030.53 | 233.79 | B | 2970.63 | 177.992 |
| **7** |  | 2 | _ | 8.1793 | 0.06034 | 8.1793 | 0.06034 | 3649.59 | 235.40 | A | 3566.36 | 215.181 |
| **8** |  | 3 | _ | 8.1896 | 0.06103 | 8.1896 | 0.06103 | 3742.00 | 238.09 | A | 3603.40 | 219.925 |


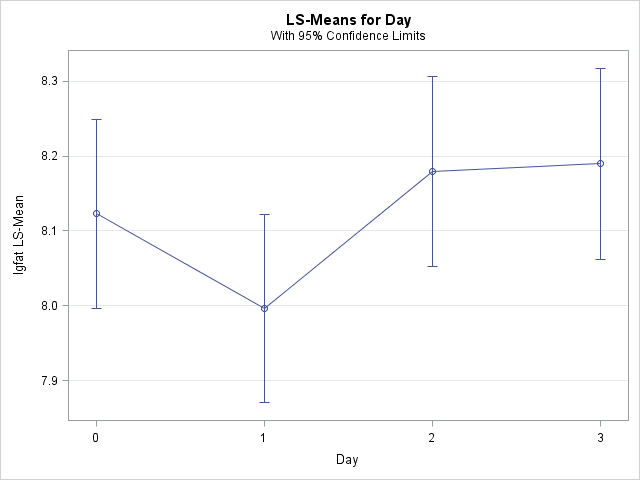


| **Differences of Day Least Squares Means** | | | | | | | | | |
| --- | --- | --- | --- | --- | --- | --- | --- | --- | --- |
| **Day** | **_Day** | **Estimate** | **Standard Error** | **DF** | **t Value** | **Pr > \|t\|** | **Alpha** | **Lower** | **Upper** |
| **0** | **1** | 0.1263 | 0.04472 | 65.32 | 2.83 | 0.0063 | 0.05 | 0.03705 | 0.2156 |
| **0** | **2** | -0.05642 | 0.04536 | 65.36 | -1.24 | 0.2180 | 0.05 | -0.1470 | 0.03415 |
| **0** | **3** | -0.06676 | 0.04613 | 65.4 | -1.45 | 0.1526 | 0.05 | -0.1589 | 0.02535 |
| **1** | **2** | -0.1828 | 0.04536 | 65.36 | -4.03 | 0.0001 | 0.05 | -0.2733 | -0.09219 |
| **1** | **3** | -0.1931 | 0.04613 | 65.4 | -4.19 | <.0001 | 0.05 | -0.2852 | -0.1010 |
| **2** | **3** | -0.01033 | 0.04683 | 65.46 | -0.22 | 0.8260 | 0.05 | -0.1038 | 0.08317 |

# Protein

Effect=Trt Method=LSD(P<.05) Set=2

| **Obs** | **Trt** | **Day** | **Period** | **Estimate** | **Standard Error** | **Mean** | **Standard Error of Mean** | **UnTrans_Mean** | **UnTrans_Stderr** | **Letter Group** |
| --- | --- | --- | --- | --- | --- | --- | --- | --- | --- | --- |
| **3** | Lying | _ | _ | 44.2281 | 6.8592 | 44.2281 | 6.8592 | 2917.09 | 109.42 | A |
| **4** | Sleep | _ | _ | 36.2343 | 6.8317 | 36.2343 | 6.8317 | 2886.56 | 108.09 | B |
