# Supplementary material for: Effects of acute lying and sleep deprivation on the behavior of lactating dairy cows
Source: PLoS One. 2019 Aug 28;14(8):e0212823. doi: 10.1371/journal.pone.0212823 (PMC6713338; doi:10.1371/journal.pone.0212823)
Supplement: S3 File — Supplemental data to support conclusions drawn on the effects of treatment on daily milk fat from cows. (DOCX) [file pone.0212823.s005.docx]

| The SAS System |
| --- |
| GLIMMix ANOVA for fat |

The GLIMMIX Procedure

| **Class Level Information** | | |
| --- | --- | --- |
| **Class** | **Levels** | **Values** |
| **Cow_ID** | 12 | 4444 4479 4481 4484 4486 4490 4507 4512 4518 6302 6725 13162 |
| **Trt** | 2 | Lying Sleep |
| **Day** | 4 | 0 1 2 3 |
| **Period** | 2 | 1 2 |

| **Number of Observations Read** | 94 |
| --- | --- |
| **Number of Observations Used** | 85 |

| Convergence criterion (GCONV=1E-8) satisfied. |
| --- |

| **Estimated G matrix is not positive definite.** |
| --- |

| **Fit Statistics** | |
| --- | --- |
| **-2 Res Log Likelihood** | -40.42 |
| **AIC (smaller is better)** | -34.42 |
| **AICC (smaller is better)** | -34.09 |
| **BIC (smaller is better)** | -32.96 |
| **CAIC (smaller is better)** | -29.96 |
| **HQIC (smaller is better)** | -34.96 |
| **Generalized Chi-Square** | 1.29 |
| **Gener. Chi-Square / DF** | 0.02 |

| **Covariance Parameter Estimates** | | |
| --- | --- | --- |
| **Cov Parm** | **Estimate** | **Standard Error** |
| **Cow_ID** | 0.02842 | 0.01314 |
| **Cow_ID*Trt*Period** | 0 | . |
| **Cow_I*Trt*Day*Period** | 0.001170 | 0.003164 |
| **Residual (VC)** | 0.01691 | . |

| **Type III Tests of Fixed Effects** | | | | |
| --- | --- | --- | --- | --- |
| **Effect** | **Num DF** | **Den DF** | **F Value** | **Pr > F** |
| **Period** | 1 | 66.74 | 0.13 | 0.7170 |
| **Trt** | 1 | 67.08 | 0.01 | 0.9266 |
| **Day** | 3 | 65.34 | 7.66 | 0.0002 |
| **Trt*Day** | 3 | 65.41 | 1.07 | 0.3700 |

| The SAS System |
| --- |
| Mean separation for log fat |
| Differences of Least Squares Means |

Effect=Period bygroup=1

| **Obs** | **ADJUSTMENT** | **adjp** | **Trt** | **Day** | **Period** | **_Trt** | **_Day** | **_Period** | **Estimate** | **StdErr** | **DF** | **tValue** | **Probt** |
| --- | --- | --- | --- | --- | --- | --- | --- | --- | --- | --- | --- | --- | --- |
| **1** | LSD(P<.05) | 0.71697 |  | _ | 1 |  | _ | 2 | -0.01119 | 0.03074 | 66.74 | -0.36 | 0.7170 |

Effect=Trt bygroup=2

| **Obs** | **ADJUSTMENT** | **adjp** | **Trt** | **Day** | **Period** | **_Trt** | **_Day** | **_Period** | **Estimate** | **StdErr** | **DF** | **tValue** | **Probt** |
| --- | --- | --- | --- | --- | --- | --- | --- | --- | --- | --- | --- | --- | --- |
| **2** | LSD(P<.05) | 0.92661 | Lying | _ | _ | Sleep | _ | _ | -0.00284 | 0.03077 | 67.08 | -0.09 | 0.9266 |

Effect=Day bygroup=3

| **Obs** | **ADJUSTMENT** | **adjp** | **Trt** | **Day** | **Period** | **_Trt** | **_Day** | **_Period** | **Estimate** | **StdErr** | **DF** | **tValue** | **Probt** |
| --- | --- | --- | --- | --- | --- | --- | --- | --- | --- | --- | --- | --- | --- |
| **3** | LSD(P<.05) | 0.00272 |  | 0 | _ |  | 1 | _ | 0.1263 | 0.04054 | 65.28 | 3.12 | 0.0027 |
| **4** | LSD(P<.05) | 0.17445 |  | 0 | _ |  | 2 | _ | -0.05646 | 0.04112 | 65.32 | -1.37 | 0.1744 |
| **5** | LSD(P<.05) | 0.55111 |  | 0 | _ |  | 3 | _ | -0.02506 | 0.04182 | 65.35 | -0.60 | 0.5511 |
| **6** | LSD(P<.05) | 0.00003 |  | 1 | _ |  | 2 | _ | -0.1828 | 0.04112 | 65.32 | -4.45 | <.0001 |
| **7** | LSD(P<.05) | 0.00057 |  | 1 | _ |  | 3 | _ | -0.1514 | 0.04182 | 65.35 | -3.62 | 0.0006 |
| **8** | LSD(P<.05) | 0.46214 |  | 2 | _ |  | 3 | _ | 0.03140 | 0.04245 | 65.41 | 0.74 | 0.4621 |

Effect=Trt*Day bygroup=4

| **Obs** | **ADJUSTMENT** | **adjp** | **Trt** | **Day** | **Period** | **_Trt** | **_Day** | **_Period** | **Estimate** | **StdErr** | **DF** | **tValue** | **Probt** |
| --- | --- | --- | --- | --- | --- | --- | --- | --- | --- | --- | --- | --- | --- |
| **9** | LSD(P<.05) | 0.02669 | Lying | 0 | _ | Lying | 1 | _ | 0.1300 | 0.05733 | 65.28 | 2.27 | 0.0267 |
| **10** | LSD(P<.05) | 0.14225 | Lying | 0 | _ | Lying | 2 | _ | -0.08759 | 0.05897 | 65.36 | -1.49 | 0.1422 |
| **11** | LSD(P<.05) | 0.15633 | Lying | 0 | _ | Lying | 3 | _ | -0.08741 | 0.06095 | 65.43 | -1.43 | 0.1563 |
| **12** | LSD(P<.05) | 0.41306 | Lying | 0 | _ | Sleep | 0 | _ | -0.04776 | 0.05798 | 65.72 | -0.82 | 0.4131 |
| **13** | LSD(P<.05) | 0.20071 | Lying | 0 | _ | Sleep | 1 | _ | 0.07494 | 0.05798 | 65.72 | 1.29 | 0.2007 |
| **14** | LSD(P<.05) | 0.21189 | Lying | 0 | _ | Sleep | 2 | _ | -0.07309 | 0.05798 | 65.72 | -1.26 | 0.2119 |
| **15** | LSD(P<.05) | 0.85813 | Lying | 0 | _ | Sleep | 3 | _ | -0.01047 | 0.05834 | 65.86 | -0.18 | 0.8581 |
| **16** | LSD(P<.05) | 0.00046 | Lying | 1 | _ | Lying | 2 | _ | -0.2176 | 0.05897 | 65.36 | -3.69 | 0.0005 |
| **17** | LSD(P<.05) | 0.00068 | Lying | 1 | _ | Lying | 3 | _ | -0.2174 | 0.06095 | 65.43 | -3.57 | 0.0007 |
| **18** | LSD(P<.05) | 0.00315 | Lying | 1 | _ | Sleep | 0 | _ | -0.1778 | 0.05798 | 65.72 | -3.07 | 0.0031 |
| **19** | LSD(P<.05) | 0.34585 | Lying | 1 | _ | Sleep | 1 | _ | -0.05505 | 0.05798 | 65.72 | -0.95 | 0.3459 |
| **20** | LSD(P<.05) | 0.00083 | Lying | 1 | _ | Sleep | 2 | _ | -0.2031 | 0.05798 | 65.72 | -3.50 | 0.0008 |
| **21** | LSD(P<.05) | 0.01887 | Lying | 1 | _ | Sleep | 3 | _ | -0.1405 | 0.05834 | 65.86 | -2.41 | 0.0189 |
| **22** | LSD(P<.05) | 0.99768 | Lying | 2 | _ | Lying | 3 | _ | 0.000183 | 0.06268 | 65.53 | 0.00 | 0.9977 |
| **23** | LSD(P<.05) | 0.50677 | Lying | 2 | _ | Sleep | 0 | _ | 0.03983 | 0.05967 | 65.82 | 0.67 | 0.5068 |
| **24** | LSD(P<.05) | 0.00825 | Lying | 2 | _ | Sleep | 1 | _ | 0.1625 | 0.05967 | 65.82 | 2.72 | 0.0083 |
| **25** | LSD(P<.05) | 0.80878 | Lying | 2 | _ | Sleep | 2 | _ | 0.01450 | 0.05967 | 65.82 | 0.24 | 0.8088 |
| **26** | LSD(P<.05) | 0.20338 | Lying | 2 | _ | Sleep | 3 | _ | 0.07712 | 0.06003 | 65.96 | 1.28 | 0.2034 |
| **27** | LSD(P<.05) | 0.52290 | Lying | 3 | _ | Sleep | 0 | _ | 0.03965 | 0.06172 | 65.92 | 0.64 | 0.5229 |
| **28** | LSD(P<.05) | 0.01061 | Lying | 3 | _ | Sleep | 1 | _ | 0.1623 | 0.06172 | 65.92 | 2.63 | 0.0106 |
| **29** | LSD(P<.05) | 0.81733 | Lying | 3 | _ | Sleep | 2 | _ | 0.01431 | 0.06172 | 65.92 | 0.23 | 0.8173 |
| **30** | LSD(P<.05) | 0.22221 | Lying | 3 | _ | Sleep | 3 | _ | 0.07694 | 0.06244 | 66.16 | 1.23 | 0.2222 |
| **31** | LSD(P<.05) | 0.03608 | Sleep | 0 | _ | Sleep | 1 | _ | 0.1227 | 0.05733 | 65.28 | 2.14 | 0.0361 |
| **32** | LSD(P<.05) | 0.66007 | Sleep | 0 | _ | Sleep | 2 | _ | -0.02533 | 0.05733 | 65.28 | -0.44 | 0.6601 |
| **33** | LSD(P<.05) | 0.52017 | Sleep | 0 | _ | Sleep | 3 | _ | 0.03729 | 0.05768 | 65.41 | 0.65 | 0.5202 |
| **34** | LSD(P<.05) | 0.01208 | Sleep | 1 | _ | Sleep | 2 | _ | -0.1480 | 0.05733 | 65.28 | -2.58 | 0.0121 |
| **35** | LSD(P<.05) | 0.14344 | Sleep | 1 | _ | Sleep | 3 | _ | -0.08541 | 0.05768 | 65.41 | -1.48 | 0.1434 |
| **36** | LSD(P<.05) | 0.28156 | Sleep | 2 | _ | Sleep | 3 | _ | 0.06262 | 0.05768 | 65.41 | 1.09 | 0.2816 |

| The SAS System |
| --- |
| Mean separation for log fat |
| Differences of Least Squares Means |

| **Set** | **Average Sig Diff Value** | **Minimum Sig Diff Value** | **Maximum Sig Diff Value** |
| --- | --- | --- | --- |
| 1 | 0.06136 | 0.06136 | 0.06136 |
| 2 | 0.06142 | 0.06142 | 0.06142 |
| 3 | 0.08283 | 0.08096 | 0.08478 |
| 4 | 0.1181 | 0.11449 | 0.12515 |

| The SAS System |
| --- |
| Back-transformed (bt) Mean Separation for log fat |

Effect=Period Method=LSD(P<.05) Set=1

| **Obs** | **Trt** | **Day** | **Period** | **Estimate** | **Standard Error** | **Mean** | **Standard Error of Mean** | **UnTrans_Mean** | **UnTrans_Stderr** | **Letter Group** | **BT_Mean** | **BT_StdErr** |
| --- | --- | --- | --- | --- | --- | --- | --- | --- | --- | --- | --- | --- |
| **1** |  | _ | 1 | 1.2003 | 0.05405 | 1.2003 | 0.05405 | 3.3912 | 0.1811 | A | 3.32114 | 0.17952 |
| **2** |  | _ | 2 | 1.2115 | 0.05249 | 1.2115 | 0.05249 | 3.4423 | 0.1760 | A | 3.35852 | 0.17629 |

Effect=Trt Method=LSD(P<.05) Set=2

| **Obs** | **Trt** | **Day** | **Period** | **Estimate** | **Standard Error** | **Mean** | **Standard Error of Mean** | **UnTrans_Mean** | **UnTrans_Stderr** | **Letter Group** | **BT_Mean** | **BT_StdErr** |
| --- | --- | --- | --- | --- | --- | --- | --- | --- | --- | --- | --- | --- |
| **3** | Lying | _ | _ | 1.2045 | 0.05346 | 1.2045 | 0.05346 | 3.4127 | 0.1791 | A | 3.33503 | 0.17828 |
| **4** | Sleep | _ | _ | 1.2073 | 0.05311 | 1.2073 | 0.05311 | 3.4208 | 0.1780 | A | 3.34453 | 0.17762 |

Effect=Day Method=LSD(P<.05) Set=3

| **Obs** | **Trt** | **Day** | **Period** | **Estimate** | **Standard Error** | **Mean** | **Standard Error of Mean** | **UnTrans_Mean** | **UnTrans_Stderr** | **Letter Group** | **BT_Mean** | **BT_StdErr** |
| --- | --- | --- | --- | --- | --- | --- | --- | --- | --- | --- | --- | --- |
| **5** |  | 0 | _ | 1.2171 | 0.05665 | 1.2171 | 0.05665 | 3.4406 | 0.1896 | A | 3.37742 | 0.19134 |
| **6** |  | 1 | _ | 1.0908 | 0.05665 | 1.0908 | 0.05665 | 3.0412 | 0.1896 | B | 2.97655 | 0.16863 |
| **7** |  | 2 | _ | 1.2736 | 0.05702 | 1.2736 | 0.05702 | 3.6606 | 0.1908 | A | 3.57360 | 0.20376 |
| **8** |  | 3 | _ | 1.2422 | 0.05762 | 1.2422 | 0.05762 | 3.5246 | 0.1928 | A | 3.46312 | 0.19956 |

Effect=Trt*Day Method=LSD(P<.05) Set=4

| **Obs** | **Trt** | **Day** | **Period** | **Estimate** | **Standard Error** | **Mean** | **Standard Error of Mean** | **UnTrans_Mean** | **UnTrans_Stderr** | **Letter Group** | **BT_Mean** | **BT_StdErr** |
| --- | --- | --- | --- | --- | --- | --- | --- | --- | --- | --- | --- | --- |
| **9** | Lying | 0 | _ | 1.1932 | 0.06364 | 1.1932 | 0.06364 | 3.3495 | 0.2125 | AB | 3.29772 | 0.20987 |
| **10** | Lying | 1 | _ | 1.0632 | 0.06364 | 1.0632 | 0.06364 | 2.9628 | 0.2125 | C | 2.89573 | 0.18428 |
| **11** | Lying | 2 | _ | 1.2808 | 0.06505 | 1.2808 | 0.06505 | 3.6715 | 0.2172 | A | 3.59960 | 0.23417 |
| **12** | Lying | 3 | _ | 1.2806 | 0.06707 | 1.2806 | 0.06707 | 3.6670 | 0.2238 | A | 3.59894 | 0.24140 |
| **13** | Sleep | 0 | _ | 1.2410 | 0.06364 | 1.2410 | 0.06364 | 3.5318 | 0.2125 | A | 3.45905 | 0.22013 |
| **14** | Sleep | 1 | _ | 1.1183 | 0.06364 | 1.1183 | 0.06364 | 3.1196 | 0.2125 | BC | 3.05962 | 0.19472 |
| **15** | Sleep | 2 | _ | 1.2663 | 0.06364 | 1.2663 | 0.06364 | 3.6496 | 0.2125 | A | 3.54779 | 0.22578 |
| **16** | Sleep | 3 | _ | 1.2037 | 0.06396 | 1.2037 | 0.06396 | 3.3822 | 0.2136 | AB | 3.33243 | 0.21315 |

| The SAS System |
| --- |
| Plot of day*trt least squares means for log fat |


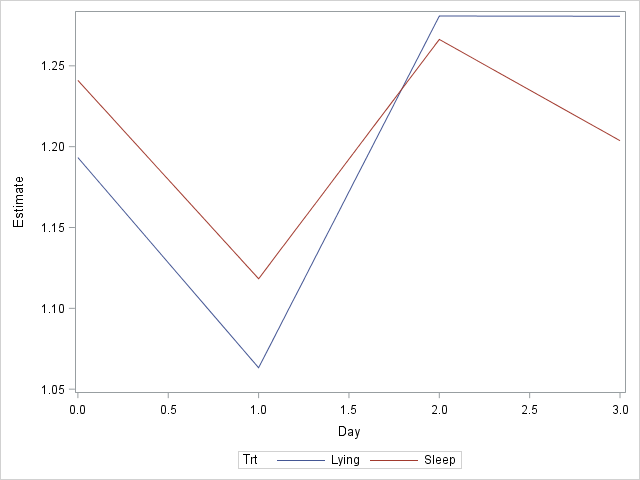


| The SAS System |
| --- |
| Plot of day*trt back-transformed means for fat |


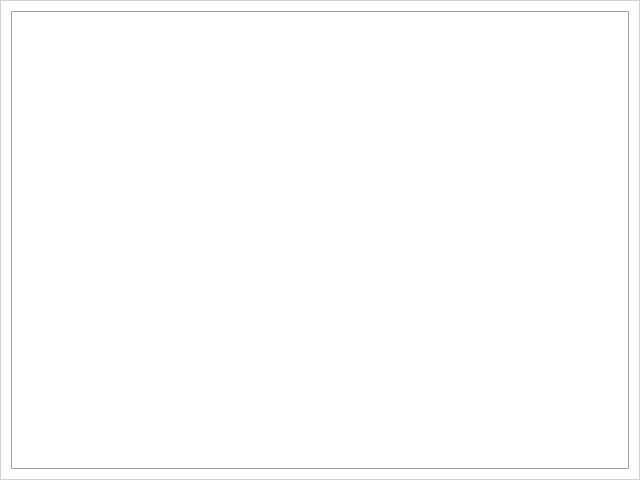


| The SAS System |
| --- |
| Plot of day*trt untransformed least squares means for fat |


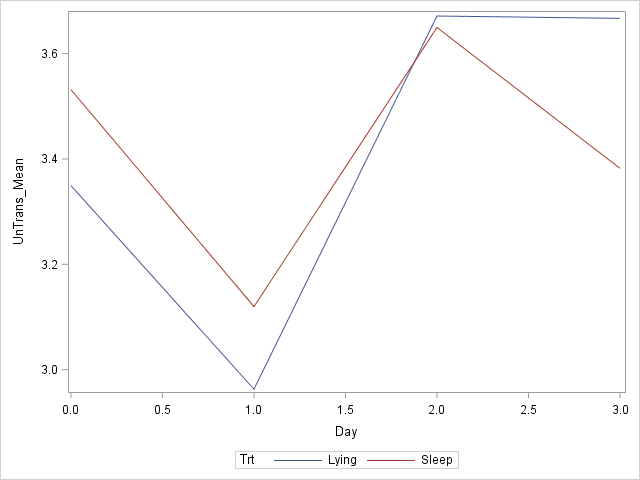


| The SAS System |
| --- |
| Check on normality for log fat |

The UNIVARIATE Procedure

Variable: residual (Residual (Mu scale))

| **Tests for Normality** | | | | |
| --- | --- | --- | --- | --- |
| **Test** | **Statistic** | | **p Value** | |
| **Shapiro-Wilk** | **W** | 0.959173 | **Pr < W** | 0.0088 |
| **Kolmogorov-Smirnov** | **D** | 0.072093 | **Pr > D** | >0.1500 |
| **Cramer-von Mises** | **W-Sq** | 0.102855 | **Pr > W-Sq** | 0.1021 |
| **Anderson-Darling** | **A-Sq** | 0.631695 | **Pr > A-Sq** | 0.0973 |

| **Extreme Observations** | | | |
| --- | --- | --- | --- |
| **Lowest** | | **Highest** | |
| **Value** | **Obs** | **Value** | **Obs** |
| -0.463561 | 36 | 0.177098 | 71 |
| -0.202644 | 26 | 0.189199 | 89 |
| -0.197782 | 50 | 0.222017 | 73 |
| -0.189651 | 92 | 0.236740 | 12 |
| -0.174291 | 63 | 0.267568 | 37 |

| **Missing Values** | | | |
| --- | --- | --- | --- |
| **Missing Value** | **Count** | **Percent Of** | |
|  |  | **All Obs** | **Missing Obs** |
| . | 9 | 9.57 | 100.00 |
