# Supplementary material for: Effects of acute lying and sleep deprivation on the behavior of lactating dairy cows
Source: PLoS One. 2019 Aug 28;14(8):e0212823. doi: 10.1371/journal.pone.0212823 (PMC6713338; doi:10.1371/journal.pone.0212823)
Supplement: S4 File — Supplemental data from revised SAS model to support conclusions drawn on the effects of treatment on daily milk fat from cows. (DOCX) [file pone.0212823.s006.docx]

Fat: looking at it by dat and day*trt interaction. Could not look at specific times and day interaction because not equal samples taken across baseline, trt or Recov

| The SAS System |
| --- |
| GLIMMix ANOVA for fat |

The GLIMMIX Procedure

| **Class Level Information** | | |
| --- | --- | --- |
| **Class** | **Levels** | **Values** |
| **Cow_ID** | 12 | 4444 4479 4481 4484 4486 4490 4507 4512 4518 6302 6725 13162 |
| **Day** | 3 | 0 1 2 |
| **Trt** | 2 | Lying Sleep |
| **Period** | 2 | 1 2 |

| **Number of Observations Read** | 70 |
| --- | --- |
| **Number of Observations Used** | 64 |

| Convergence criterion (GCONV=1E-8) satisfied. |
| --- |

| **Fit Statistics** | |
| --- | --- |
| **-2 Res Log Likelihood** | -26.88 |
| **AIC (smaller is better)** | -18.88 |
| **AICC (smaller is better)** | -18.11 |
| **BIC (smaller is better)** | -16.94 |
| **CAIC (smaller is better)** | -12.94 |
| **HQIC (smaller is better)** | -19.59 |
| **Generalized Chi-Square** | 0.91 |
| **Gener. Chi-Square / DF** | 0.02 |

| **Covariance Parameter Estimates** | | | |
| --- | --- | --- | --- |
| **Cov Parm** | **Subject** | **Estimate** | **Standard Error** |
| **Cow_ID** |  | 0.03148 | 0.01476 |
| **Cow_ID*Trt*Period** |  | 0.001382 | 0.003284 |
| **AR(1)** | **Cow_ID*Trt*Period** | -0.1466 | 0.2612 |
| **Residual** |  | 0.01598 | 0.003945 |

| **Type III Tests of Fixed Effects** | | | | |
| --- | --- | --- | --- | --- |
| **Effect** | **Num DF** | **Den DF** | **F Value** | **Pr > F** |
| **Period** | 1 | 9.064 | 0.30 | 0.5991 |
| **Trt** | 1 | 9.509 | 0.65 | 0.4385 |
| **Day** | 2 | 29.05 | 2.91 | 0.0705 |
| **Day*Trt** | 2 | 28.89 | 3.96 | 0.0303 |

| The SAS System |
| --- |
| Mean separation for log fat |
| Differences of Least Squares Means |

Effect=Period bygroup=1

| **Obs** | **ADJUSTMENT** | **adjp** | **Day** | **Trt** | **Period** | **_Day** | **_Trt** | **_Period** | **Estimate** | **StdErr** | **DF** | **tValue** | **Probt** |
| --- | --- | --- | --- | --- | --- | --- | --- | --- | --- | --- | --- | --- | --- |
| **1** | LSD(P<.05) | 0.59910 | _ |  | 1 | _ |  | 2 | -0.01851 | 0.03398 | 9.064 | -0.54 | 0.5991 |

Effect=Trt bygroup=2

| **Obs** | **ADJUSTMENT** | **adjp** | **Day** | **Trt** | **Period** | **_Day** | **_Trt** | **_Period** | **Estimate** | **StdErr** | **DF** | **tValue** | **Probt** |
| --- | --- | --- | --- | --- | --- | --- | --- | --- | --- | --- | --- | --- | --- |
| **2** | LSD(P<.05) | 0.43854 | _ | Lying | _ | _ | Sleep | _ | 0.02747 | 0.03397 | 9.509 | 0.81 | 0.4385 |

Effect=Day bygroup=3

| **Obs** | **ADJUSTMENT** | **adjp** | **Day** | **Trt** | **Period** | **_Day** | **_Trt** | **_Period** | **Estimate** | **StdErr** | **DF** | **tValue** | **Probt** |
| --- | --- | --- | --- | --- | --- | --- | --- | --- | --- | --- | --- | --- | --- |
| **3** | LSD(P<.05) | 0.44582 | 0 |  | _ | 1 |  | _ | 0.03193 | 0.04131 | 28.77 | 0.77 | 0.4458 |
| **4** | LSD(P<.05) | 0.10047 | 0 |  | _ | 2 |  | _ | -0.06896 | 0.04053 | 26.72 | -1.70 | 0.1005 |
| **5** | LSD(P<.05) | 0.02450 | 1 |  | _ | 2 |  | _ | -0.1009 | 0.04272 | 31.66 | -2.36 | 0.0245 |

Effect=Day*Trt bygroup=4

| **Obs** | **ADJUSTMENT** | **adjp** | **Day** | **Trt** | **Period** | **_Day** | **_Trt** | **_Period** | **Estimate** | **StdErr** | **DF** | **tValue** | **Probt** |
| --- | --- | --- | --- | --- | --- | --- | --- | --- | --- | --- | --- | --- | --- |
| **6** | LSD(P<.05) | 0.41785 | 0 | Lying | _ | 0 | Sleep | _ | -0.04678 | 0.05714 | 39.51 | -0.82 | 0.4179 |
| **7** | LSD(P<.05) | 0.65377 | 0 | Lying | _ | 1 | Lying | _ | 0.02645 | 0.05834 | 28.6 | 0.45 | 0.6538 |
| **8** | LSD(P<.05) | 0.87077 | 0 | Lying | _ | 1 | Sleep | _ | -0.00936 | 0.05716 | 39.27 | -0.16 | 0.8708 |
| **9** | LSD(P<.05) | 0.00652 | 0 | Lying | _ | 2 | Lying | _ | -0.1749 | 0.05922 | 26.56 | -2.95 | 0.0065 |
| **10** | LSD(P<.05) | 0.86527 | 0 | Lying | _ | 2 | Sleep | _ | -0.00986 | 0.05773 | 39.94 | -0.17 | 0.8653 |
| **11** | LSD(P<.05) | 0.20748 | 0 | Sleep | _ | 1 | Lying | _ | 0.07323 | 0.05714 | 39.51 | 1.28 | 0.2075 |
| **12** | LSD(P<.05) | 0.52741 | 0 | Sleep | _ | 1 | Sleep | _ | 0.03742 | 0.05850 | 28.94 | 0.64 | 0.5274 |
| **13** | LSD(P<.05) | 0.04370 | 0 | Sleep | _ | 2 | Lying | _ | -0.1281 | 0.06154 | 41.08 | -2.08 | 0.0437 |
| **14** | LSD(P<.05) | 0.51421 | 0 | Sleep | _ | 2 | Sleep | _ | 0.03693 | 0.05585 | 26.54 | 0.66 | 0.5142 |
| **15** | LSD(P<.05) | 0.53468 | 1 | Lying | _ | 1 | Sleep | _ | -0.03580 | 0.05716 | 39.27 | -0.63 | 0.5347 |
| **16** | LSD(P<.05) | 0.00289 | 1 | Lying | _ | 2 | Lying | _ | -0.2013 | 0.06237 | 31.81 | -3.23 | 0.0029 |
| **17** | LSD(P<.05) | 0.53301 | 1 | Lying | _ | 2 | Sleep | _ | -0.03630 | 0.05773 | 39.94 | -0.63 | 0.5330 |
| **18** | LSD(P<.05) | 0.01026 | 1 | Sleep | _ | 2 | Lying | _ | -0.1655 | 0.06148 | 40.71 | -2.69 | 0.0103 |
| **19** | LSD(P<.05) | 0.99331 | 1 | Sleep | _ | 2 | Sleep | _ | -0.00050 | 0.05895 | 31.54 | -0.01 | 0.9933 |
| **20** | LSD(P<.05) | 0.01163 | 2 | Lying | _ | 2 | Sleep | _ | 0.1650 | 0.06253 | 41.93 | 2.64 | 0.0116 |

| The SAS System |
| --- |
| Mean separation for log fat |
| Differences of Least Squares Means |

| **Set** | **Average Sig Diff Value** | **Minimum Sig Diff Value** | **Maximum Sig Diff Value** |
| --- | --- | --- | --- |
| 1 | 0.07678 | 0.07678 | 0.07678 |
| 2 | 0.07623 | 0.07623 | 0.07623 |
| 3 | 0.08493 | 0.08321 | 0.08705 |
| 4 | 0.11952 | 0.11469 | 0.12708 |

| The SAS System |
| --- |
| Back-transformed (bt) Mean Separation for log fat |

Effect=Period Method=LSD(P<.05) Set=1

| **Obs** | **Day** | **Trt** | **Period** | **Estimate** | **Standard Error** | **Mean** | **Standard Error of Mean** | **UnTrans_Mean** | **UnTrans_Stderr** | **Letter Group** | **BT_Mean** | **BT_StdErr** |
| --- | --- | --- | --- | --- | --- | --- | --- | --- | --- | --- | --- | --- |
| **1** | _ |  | 1 | 1.2182 | 0.05761 | 1.2182 | 0.05761 | 3.4410 | 0.2167 | A | 3.38099 | 0.19477 |
| **2** | _ |  | 2 | 1.2367 | 0.05545 | 1.2367 | 0.05545 | 3.5557 | 0.2034 | A | 3.44414 | 0.19099 |

Effect=Trt Method=LSD(P<.05) Set=2

| **Obs** | **Day** | **Trt** | **Period** | **Estimate** | **Standard Error** | **Mean** | **Standard Error of Mean** | **UnTrans_Mean** | **UnTrans_Stderr** | **Letter Group** | **BT_Mean** | **BT_StdErr** |
| --- | --- | --- | --- | --- | --- | --- | --- | --- | --- | --- | --- | --- |
| **3** | _ | Lying | _ | 1.2412 | 0.05676 | 1.2412 | 0.05676 | 3.5798 | 0.2112 | A | 3.45961 | 0.19638 |
| **4** | _ | Sleep | _ | 1.2137 | 0.05631 | 1.2137 | 0.05631 | 3.4168 | 0.2087 | A | 3.36587 | 0.18955 |

Effect=Day Method=LSD(P<.05) Set=3

| **Obs** | **Day** | **Trt** | **Period** | **Estimate** | **Standard Error** | **Mean** | **Standard Error of Mean** | **UnTrans_Mean** | **UnTrans_Stderr** | **Letter Group** | **BT_Mean** | **BT_StdErr** |
| --- | --- | --- | --- | --- | --- | --- | --- | --- | --- | --- | --- | --- |
| **5** | 0 |  | _ | 1.2151 | 0.05865 | 1.2151 | 0.05865 | 3.4289 | 0.2178 | AB | 3.37056 | 0.19768 |
| **6** | 1 |  | _ | 1.1831 | 0.05866 | 1.1831 | 0.05866 | 3.3178 | 0.2178 | B | 3.26462 | 0.19149 |
| **7** | 2 |  | _ | 1.2840 | 0.05974 | 1.2840 | 0.05974 | 3.7482 | 0.2238 | A | 3.61121 | 0.21574 |

Effect=Day*Trt Method=LSD(P<.05) Set=4

| **Obs** | **Day** | **Trt** | **Period** | **Estimate** | **Standard Error** | **Mean** | **Standard Error of Mean** | **UnTrans_Mean** | **UnTrans_Stderr** | **Letter Group** | **BT_Mean** | **BT_StdErr** |
| --- | --- | --- | --- | --- | --- | --- | --- | --- | --- | --- | --- | --- |
| **8** | 0 | Lying | _ | 1.1917 | 0.06520 | 1.1917 | 0.06520 | 3.3396 | 0.2532 | B | 3.29263 | 0.21467 |
| **9** | 0 | Sleep | _ | 1.2385 | 0.06528 | 1.2385 | 0.06528 | 3.5182 | 0.2537 | B | 3.45033 | 0.22523 |
| **10** | 1 | Lying | _ | 1.1652 | 0.06520 | 1.1652 | 0.06520 | 3.2643 | 0.2532 | B | 3.20670 | 0.20907 |
| **11** | 1 | Sleep | _ | 1.2010 | 0.06530 | 1.2010 | 0.06530 | 3.3714 | 0.2537 | B | 3.32359 | 0.21703 |
| **12** | 2 | Lying | _ | 1.3665 | 0.06903 | 1.3665 | 0.06903 | 4.1355 | 0.2732 | A | 3.92176 | 0.27073 |
| **13** | 2 | Sleep | _ | 1.2015 | 0.06578 | 1.2015 | 0.06578 | 3.3610 | 0.2564 | B | 3.32525 | 0.21875 |

| The SAS System |
| --- |
| Plot of day*trt least squares means for log fat |

| SAS System |
| --- |
| Plot of day*trt untransformed least squares means for fat |

| The SAS System |
| --- |
| Check on normality for log fat |

The UNIVARIATE Procedure

Variable: residual (Residual (Mu scale))

| **Tests for Normality** | | | | |
| --- | --- | --- | --- | --- |
| **Test** | **Statistic** | | **p Value** | |
| **Shapiro-Wilk** | **W** | 0.945218 | **Pr < W** | 0.0067 |
| **Kolmogorov-Smirnov** | **D** | 0.107465 | **Pr > D** | 0.0664 |
| **Cramer-von Mises** | **W-Sq** | 0.08009 | **Pr > W-Sq** | 0.2105 |
| **Anderson-Darling** | **A-Sq** | 0.555402 | **Pr > A-Sq** | 0.1491 |

| **Extreme Observations** | | | |
| --- | --- | --- | --- |
| **Lowest** | | **Highest** | |
| **Value** | **Obs** | **Value** | **Obs** |
| -0.187536 | 38 | 0.151138 | 58 |
| -0.173616 | 1 | 0.171016 | 56 |
| -0.168531 | 57 | 0.185741 | 50 |
| -0.167660 | 70 | 0.226914 | 34 |
| -0.162358 | 12 | 0.429425 | 67 |

| **Missing Values** | | | |
| --- | --- | --- | --- |
| **Missing Value** | **Count** | **Percent Of** | |
|  |  | **All Obs** | **Missing Obs** |
| . | 6 | 8.57 | 100.00 |
