# Supplementary material for: Effects of acute lying and sleep deprivation on the behavior of lactating dairy cows
Source: PLoS One. 2019 Aug 28;14(8):e0212823. doi: 10.1371/journal.pone.0212823 (PMC6713338; doi:10.1371/journal.pone.0212823)
Supplement: S5 File — Supplemental data from revised SAS model to support conclusions drawn on the effects of treatment on daily milk fat from cows. (DOCX) [file pone.0212823.s007.docx]

| **Number of Observations Read** | 94 |
| --- | --- |
| **Number of Observations Used** | 85 |

| **Dimensions** | |
| --- | --- |
| **G-side Cov. Parameters** | 3 |
| **R-side Cov. Parameters** | 1 |
| **Columns in X** | 17 |
| **Columns in Z** | 131 |
| **Subjects (Blocks in V)** | 1 |
| **Max Obs per Subject** | 85 |

| **Optimization Information** | |
| --- | --- |
| **Optimization Technique** | Dual Quasi-Newton |
| **Parameters in Optimization** | 3 |
| **Lower Boundaries** | 3 |
| **Upper Boundaries** | 0 |
| **Fixed Effects** | Profiled |
| **Residual Variance** | Profiled |
| **Starting From** | Data |

| **Iteration History** | | | | | |
| --- | --- | --- | --- | --- | --- |
| **Iteration** | **Restarts** | **Evaluations** | **Objective Function** | **Change** | **Max Gradient** |
| **0** | **0** | 4 | 152.32955155 | . | 31.28421 |
| **1** | **0** | 2 | 143.40423634 | 8.92531521 | 5.741566 |
| **2** | **0** | 3 | 143.23106465 | 0.17317170 | 5.395552 |
| **3** | **0** | 8 | 141.95716244 | 1.27390220 | 0.856892 |
| **4** | **0** | 6 | 141.87345164 | 0.08371080 | 0.109831 |
| **5** | **0** | 4 | 141.87159536 | 0.00185628 | 0.003027 |
| **6** | **0** | 3 | 141.87159388 | 0.00000148 | 0.000028 |
| **7** | **0** | 3 | 141.87159388 | 0.00000000 | 5.49E-10 |

| Convergence criterion (GCONV=1E-8) satisfied. |
| --- |

| **Estimated G matrix is not positive definite.** |
| --- |

| **Fit Statistics** | |
| --- | --- |
| **-2 Res Log Likelihood** | 141.87 |
| **AIC (smaller is better)** | 147.87 |
| **AICC (smaller is better)** | 148.20 |
| **BIC (smaller is better)** | 149.33 |
| **CAIC (smaller is better)** | 152.33 |
| **HQIC (smaller is better)** | 147.33 |
| **Generalized Chi-Square** | 15.07 |
| **Gener. Chi-Square / DF** | 0.20 |

| **Covariance Parameter Estimates** | | |
| --- | --- | --- |
| **Cov Parm** | **Estimate** | **Standard Error** |
| **cow** | 0.3207 | 0.1479 |
| **cow*period2*Trt** | 0 | . |
| **cow*period2*Trt*Day** | 3.314E-9 | 0.03470 |
| **Residual (VC)** | 0.1983 | . |

| **Type III Tests of Fixed Effects** | | | | |
| --- | --- | --- | --- | --- |
| **Effect** | **Num DF** | **Den DF** | **F Value** | **Pr > F** |
| **period2** | 1 | 66.71 | 0.25 | 0.6170 |
| **Trt** | 1 | 67.05 | 0.01 | 0.9370 |
| **Day** | 3 | 65.34 | 7.70 | 0.0002 |
| **Trt*Day** | 3 | 65.4 | 1.18 | 0.3258 |

| **Trt Least Squares Means** | | | | | | | | | | | | |
| --- | --- | --- | --- | --- | --- | --- | --- | --- | --- | --- | --- | --- |
| **Trt** | **Estimate** | **Standard Error** | **DF** | **t Value** | **Pr > \|t\|** | **Alpha** | **Lower** | **Upper** | **Mean** | **Standard Error Mean** | **Lower Mean** | **Upper Mean** |
| **Lying** | 3.4127 | 0.1791 | 13.46 | 19.05 | <.0001 | 0.05 | 3.0271 | 3.7984 | 3.4127 | 0.1791 | 3.0271 | 3.7984 |
| **Sleep** | 3.4208 | 0.1780 | 13.13 | 19.22 | <.0001 | 0.05 | 3.0367 | 3.8049 | 3.4208 | 0.1780 | 3.0367 | 3.8049 |


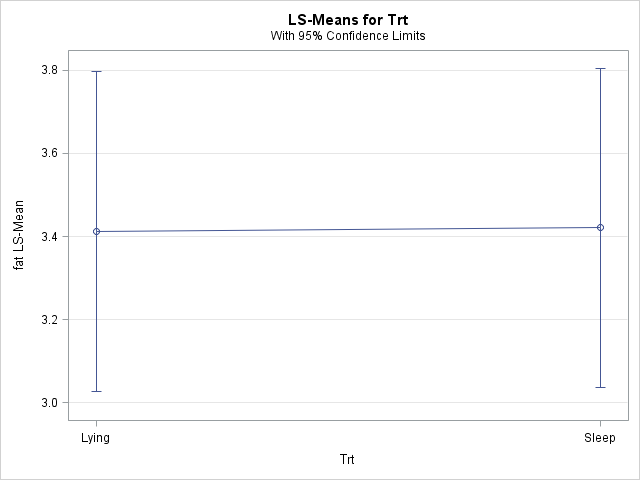


| **Differences of Trt Least Squares Means** | | | | | | | | | |
| --- | --- | --- | --- | --- | --- | --- | --- | --- | --- |
| **Trt** | **_Trt** | **Estimate** | **Standard Error** | **DF** | **t Value** | **Pr > \|t\|** | **Alpha** | **Lower** | **Upper** |
| **Lying** | **Sleep** | -0.00808 | 0.1019 | 67.05 | -0.08 | 0.9370 | 0.05 | -0.2115 | 0.1953 |

| **Day Least Squares Means** | | | | | | | | | | | | |
| --- | --- | --- | --- | --- | --- | --- | --- | --- | --- | --- | --- | --- |
| **Day** | **Estimate** | **Standard Error** | **DF** | **t Value** | **Pr > \|t\|** | **Alpha** | **Lower** | **Upper** | **Mean** | **Standard Error Mean** | **Lower Mean** | **Upper Mean** |
| **0** | 3.4406 | 0.1896 | 16.81 | 18.15 | <.0001 | 0.05 | 3.0403 | 3.8410 | 3.4406 | 0.1896 | 3.0403 | 3.8410 |
| **1** | 3.0412 | 0.1896 | 16.81 | 16.04 | <.0001 | 0.05 | 2.6409 | 3.4416 | 3.0412 | 0.1896 | 2.6409 | 3.4416 |
| **2** | 3.6606 | 0.1908 | 17.21 | 19.19 | <.0001 | 0.05 | 3.2584 | 4.0627 | 3.6606 | 0.1908 | 3.2584 | 4.0627 |
| **3** | 3.5246 | 0.1928 | 17.88 | 18.28 | <.0001 | 0.05 | 3.1194 | 3.9298 | 3.5246 | 0.1928 | 3.1194 | 3.9298 |


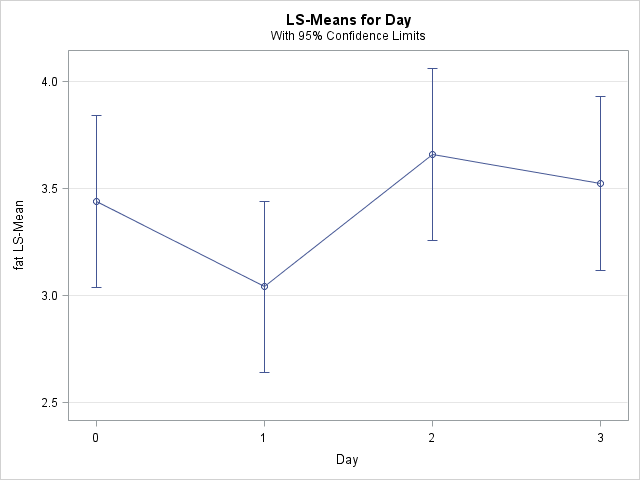


| **Differences of Day Least Squares Means** | | | | | | | | | |
| --- | --- | --- | --- | --- | --- | --- | --- | --- | --- |
| **Day** | **_Day** | **Estimate** | **Standard Error** | **DF** | **t Value** | **Pr > \|t\|** | **Alpha** | **Lower** | **Upper** |
| **0** | **1** | 0.3994 | 0.1343 | 65.28 | 2.97 | 0.0041 | 0.05 | 0.1313 | 0.6675 |
| **0** | **2** | -0.2199 | 0.1362 | 65.32 | -1.62 | 0.1111 | 0.05 | -0.4919 | 0.05200 |
| **0** | **3** | -0.08397 | 0.1385 | 65.35 | -0.61 | 0.5464 | 0.05 | -0.3605 | 0.1926 |
| **1** | **2** | -0.6193 | 0.1362 | 65.32 | -4.55 | <.0001 | 0.05 | -0.8913 | -0.3474 |
| **1** | **3** | -0.4834 | 0.1385 | 65.35 | -3.49 | 0.0009 | 0.05 | -0.7599 | -0.2068 |
| **2** | **3** | 0.1360 | 0.1406 | 65.41 | 0.97 | 0.3370 | 0.05 | -0.1448 | 0.4167 |

| **Trt*Day Least Squares Means** | | | | | | | | | | | | | |
| --- | --- | --- | --- | --- | --- | --- | --- | --- | --- | --- | --- | --- | --- |
| **Trt** | **Day** | **Estimate** | **Standard Error** | **DF** | **t Value** | **Pr > \|t\|** | **Alpha** | **Lower** | **Upper** | **Mean** | **Standard Error Mean** | **Lower Mean** | **Upper Mean** |
| **Lying** | **0** | 3.3495 | 0.2125 | 25.36 | 15.76 | <.0001 | 0.05 | 2.9121 | 3.7869 | 3.3495 | 0.2125 | 2.9121 | 3.7869 |
| **Lying** | **1** | 2.9628 | 0.2125 | 25.36 | 13.94 | <.0001 | 0.05 | 2.5255 | 3.4002 | 2.9628 | 0.2125 | 2.5255 | 3.4002 |
| **Lying** | **2** | 3.6715 | 0.2172 | 27.25 | 16.91 | <.0001 | 0.05 | 3.2261 | 4.1169 | 3.6715 | 0.2172 | 3.2261 | 4.1169 |
| **Lying** | **3** | 3.6670 | 0.2238 | 29.98 | 16.39 | <.0001 | 0.05 | 3.2100 | 4.1241 | 3.6670 | 0.2238 | 3.2100 | 4.1241 |
| **Sleep** | **0** | 3.5318 | 0.2125 | 25.36 | 16.62 | <.0001 | 0.05 | 3.0944 | 3.9691 | 3.5318 | 0.2125 | 3.0944 | 3.9691 |
| **Sleep** | **1** | 3.1196 | 0.2125 | 25.36 | 14.68 | <.0001 | 0.05 | 2.6823 | 3.5570 | 3.1196 | 0.2125 | 2.6823 | 3.5570 |
| **Sleep** | **2** | 3.6496 | 0.2125 | 25.36 | 17.17 | <.0001 | 0.05 | 3.2123 | 4.0870 | 3.6496 | 0.2125 | 3.2123 | 4.0870 |
| **Sleep** | **3** | 3.3822 | 0.2136 | 25.72 | 15.84 | <.0001 | 0.05 | 2.9429 | 3.8214 | 3.3822 | 0.2136 | 2.9429 | 3.8214 |


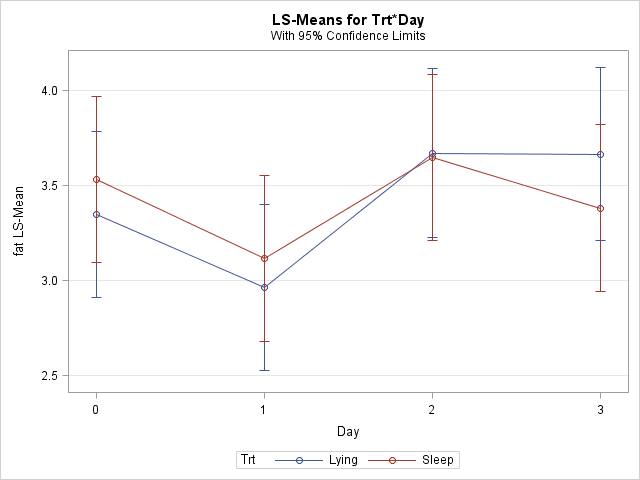


| **Differences of Trt*Day Least Squares Means** | | | | | | | | | | | |
| --- | --- | --- | --- | --- | --- | --- | --- | --- | --- | --- | --- |
| **Trt** | **Day** | **_Trt** | **_Day** | **Estimate** | **Standard Error** | **DF** | **t Value** | **Pr > \|t\|** | **Alpha** | **Lower** | **Upper** |
| **Lying** | **0** | **Lying** | **1** | 0.3867 | 0.1899 | 65.28 | 2.04 | 0.0458 | 0.05 | 0.007518 | 0.7658 |
| **Lying** | **0** | **Lying** | **2** | -0.3220 | 0.1953 | 65.36 | -1.65 | 0.1040 | 0.05 | -0.7120 | 0.06795 |
| **Lying** | **0** | **Lying** | **3** | -0.3175 | 0.2018 | 65.43 | -1.57 | 0.1205 | 0.05 | -0.7206 | 0.08554 |
| **Lying** | **0** | **Sleep** | **0** | -0.1823 | 0.1920 | 65.71 | -0.95 | 0.3460 | 0.05 | -0.5657 | 0.2011 |
| **Lying** | **0** | **Sleep** | **1** | 0.2299 | 0.1920 | 65.71 | 1.20 | 0.2356 | 0.05 | -0.1535 | 0.6133 |
| **Lying** | **0** | **Sleep** | **2** | -0.3001 | 0.1920 | 65.71 | -1.56 | 0.1228 | 0.05 | -0.6835 | 0.08326 |
| **Lying** | **0** | **Sleep** | **3** | -0.03266 | 0.1932 | 65.85 | -0.17 | 0.8663 | 0.05 | -0.4184 | 0.3531 |
| **Lying** | **1** | **Lying** | **2** | -0.7087 | 0.1953 | 65.36 | -3.63 | 0.0006 | 0.05 | -1.0986 | -0.3187 |
| **Lying** | **1** | **Lying** | **3** | -0.7042 | 0.2018 | 65.43 | -3.49 | 0.0009 | 0.05 | -1.1073 | -0.3011 |
| **Lying** | **1** | **Sleep** | **0** | -0.5689 | 0.1920 | 65.71 | -2.96 | 0.0042 | 0.05 | -0.9523 | -0.1855 |
| **Lying** | **1** | **Sleep** | **1** | -0.1568 | 0.1920 | 65.71 | -0.82 | 0.4171 | 0.05 | -0.5402 | 0.2266 |
| **Lying** | **1** | **Sleep** | **2** | -0.6868 | 0.1920 | 65.71 | -3.58 | 0.0007 | 0.05 | -1.0702 | -0.3034 |
| **Lying** | **1** | **Sleep** | **3** | -0.4193 | 0.1932 | 65.85 | -2.17 | 0.0336 | 0.05 | -0.8051 | -0.03355 |
| **Lying** | **2** | **Lying** | **3** | 0.004472 | 0.2076 | 65.53 | 0.02 | 0.9829 | 0.05 | -0.4100 | 0.4189 |
| **Lying** | **2** | **Sleep** | **0** | 0.1397 | 0.1976 | 65.81 | 0.71 | 0.4819 | 0.05 | -0.2548 | 0.5343 |
| **Lying** | **2** | **Sleep** | **1** | 0.5519 | 0.1976 | 65.81 | 2.79 | 0.0068 | 0.05 | 0.1573 | 0.9464 |
| **Lying** | **2** | **Sleep** | **2** | 0.02187 | 0.1976 | 65.81 | 0.11 | 0.9122 | 0.05 | -0.3727 | 0.4164 |
| **Lying** | **2** | **Sleep** | **3** | 0.2893 | 0.1988 | 65.94 | 1.46 | 0.1503 | 0.05 | -0.1076 | 0.6863 |
| **Lying** | **3** | **Sleep** | **0** | 0.1353 | 0.2044 | 65.91 | 0.66 | 0.5104 | 0.05 | -0.2729 | 0.5434 |
| **Lying** | **3** | **Sleep** | **1** | 0.5474 | 0.2044 | 65.91 | 2.68 | 0.0093 | 0.05 | 0.1393 | 0.9555 |
| **Lying** | **3** | **Sleep** | **2** | 0.01739 | 0.2044 | 65.91 | 0.09 | 0.9324 | 0.05 | -0.3907 | 0.4255 |
| **Lying** | **3** | **Sleep** | **3** | 0.2849 | 0.2068 | 66.14 | 1.38 | 0.1729 | 0.05 | -0.1279 | 0.6977 |
| **Sleep** | **0** | **Sleep** | **1** | 0.4121 | 0.1899 | 65.28 | 2.17 | 0.0336 | 0.05 | 0.03297 | 0.7913 |
| **Sleep** | **0** | **Sleep** | **2** | -0.1179 | 0.1899 | 65.28 | -0.62 | 0.5368 | 0.05 | -0.4970 | 0.2613 |
| **Sleep** | **0** | **Sleep** | **3** | 0.1496 | 0.1910 | 65.41 | 0.78 | 0.4363 | 0.05 | -0.2318 | 0.5310 |
| **Sleep** | **1** | **Sleep** | **2** | -0.5300 | 0.1899 | 65.28 | -2.79 | 0.0069 | 0.05 | -0.9091 | -0.1509 |
| **Sleep** | **1** | **Sleep** | **3** | -0.2625 | 0.1910 | 65.41 | -1.37 | 0.1740 | 0.05 | -0.6439 | 0.1189 |
| **Sleep** | **2** | **Sleep** | **3** | 0.2675 | 0.1910 | 65.41 | 1.40 | 0.1661 | 0.05 | -0.1139 | 0.6489 |


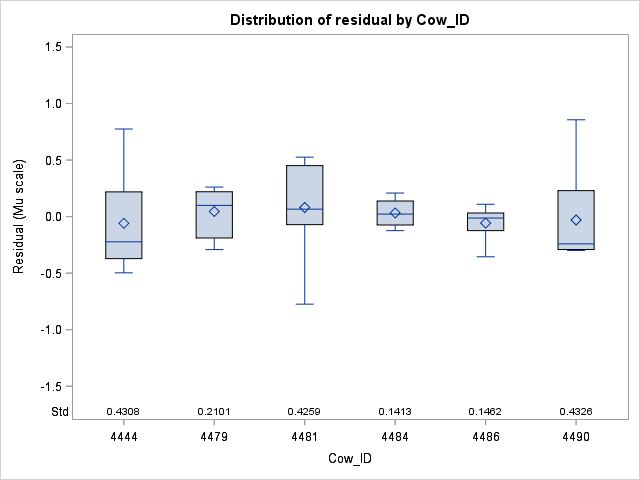


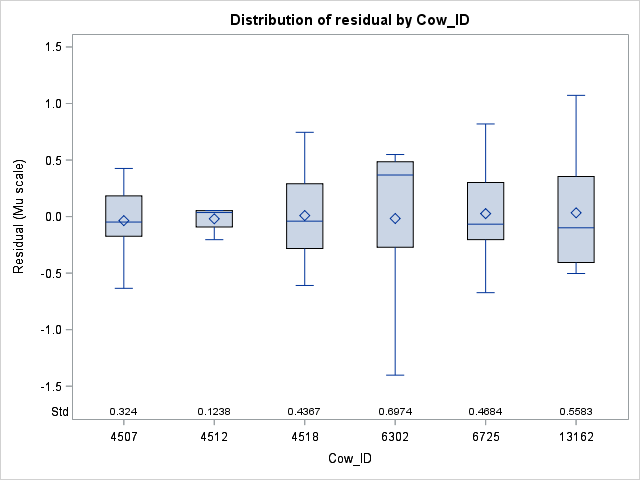


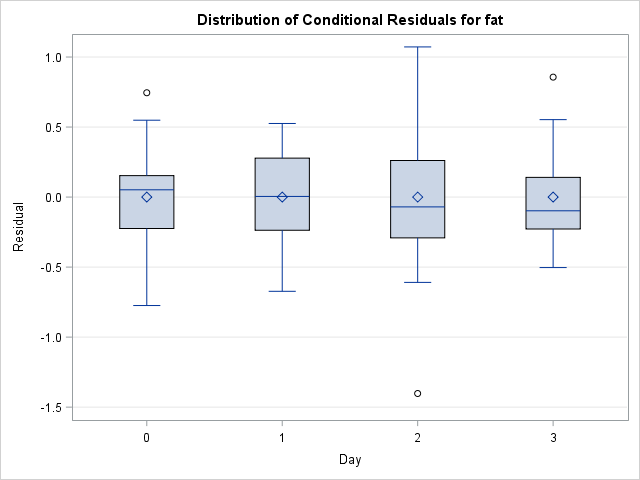


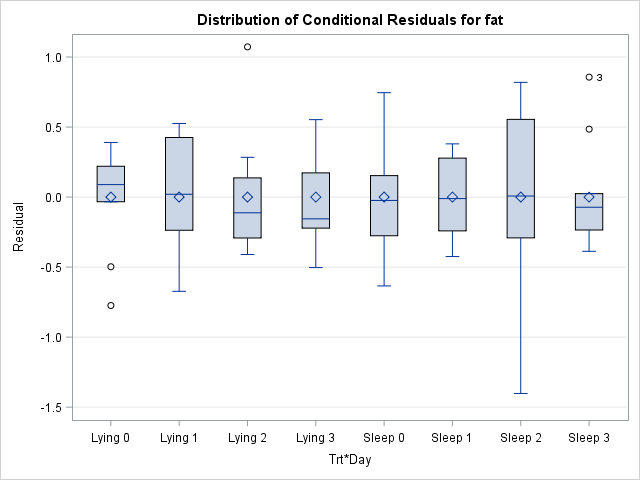


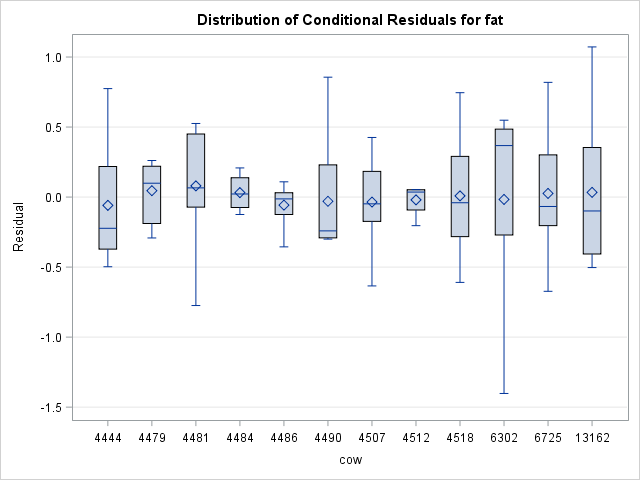


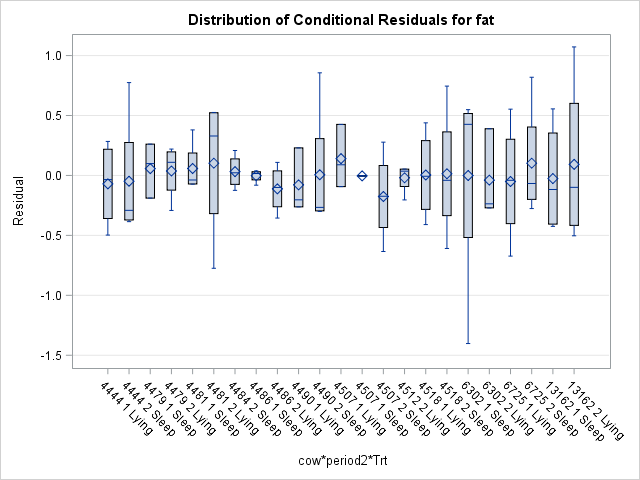


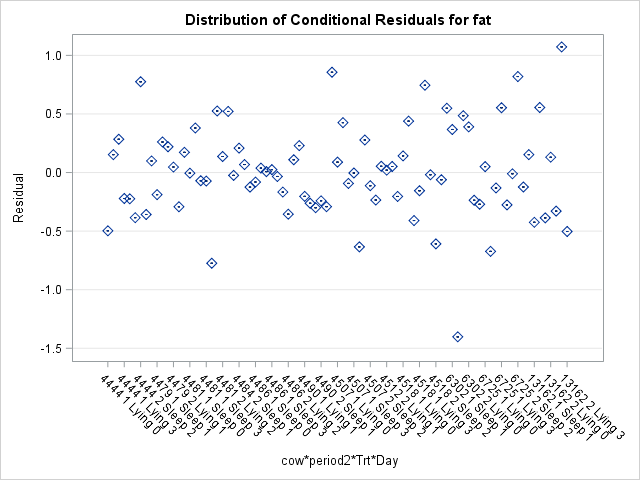


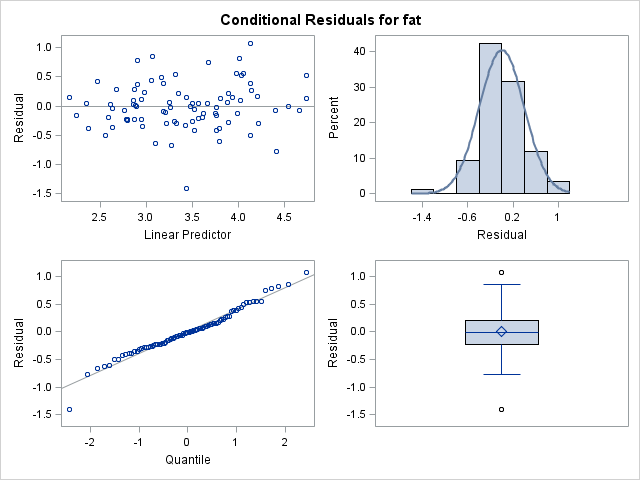


| **Tests for Normality** | | | | |
| --- | --- | --- | --- | --- |
| **Test** | **Statistic** | | **p Value** | |
| **Shapiro-Wilk** | **W** | 0.975998 | **Pr < W** | 0.1137 |
| **Kolmogorov-Smirnov** | **D** | 0.078465 | **Pr > D** | >0.1500 |
| **Cramer-von Mises** | **W-Sq** | 0.09223 | **Pr > W-Sq** | 0.1432 |
| **Anderson-Darling** | **A-Sq** | 0.578509 | **Pr > A-Sq** | 0.1337 |
