# Supplementary material for: Effects of acute lying and sleep deprivation on the behavior of lactating dairy cows
Source: PLoS One. 2019 Aug 28;14(8):e0212823. doi: 10.1371/journal.pone.0212823 (PMC6713338; doi:10.1371/journal.pone.0212823)
Supplement: S6 File — Supplemental data from SAS model to support conclusions drawn on the effects of treatment on daily milk production from cows. (DOCX) [file pone.0212823.s008.docx]

Milk Production data with the baseline being on sand bedding, NOT mattress. This way when I compare base and recover, they were on the same bedding – sand

| The SAS System |
| --- |
| GLIMMix ANOVA for TOTALkg |

The GLIMMIX Procedure

| **Class Level Information** | | |
| --- | --- | --- |
| **Class** | **Levels** | **Values** |
| **COW** | 12 | 4444 4479 4481 4484 4486 4490 4507 4512 4518 6302 6725 13162 |
| **day** | 5 | 0 2 3 4 5 |
| **TRT** | 2 | Lying Sleep |
| **period2** | 2 | 1 2 |

| **Number of Observations Read** | 120 |
| --- | --- |
| **Number of Observations Used** | 120 |

| Convergence criterion (GCONV=1E-8) satisfied. |
| --- |

| **Fit Statistics** | |
| --- | --- |
| **-2 Res Log Likelihood** | 583.90 |
| **AIC (smaller is better)** | 591.90 |
| **AICC (smaller is better)** | 592.28 |
| **BIC (smaller is better)** | 593.84 |
| **CAIC (smaller is better)** | 597.84 |
| **HQIC (smaller is better)** | 591.18 |
| **Generalized Chi-Square** | 108.86 |
| **Gener. Chi-Square / DF** | 1.00 |

| **Covariance Parameter Estimates** | | |
| --- | --- | --- |
| **Cov Parm** | **Estimate** | **Standard Error** |
| **COW** | 65.6792 | 28.8792 |
| **COW*TRT*period2** | 2.9982 | 1.8101 |
| **COW*day*TRT*period2** | 4.1720 | 0.7795 |
| **Residual (VC)** | 0.9987 | . |

| **Type III Tests of Fixed Effects** | | | | |
| --- | --- | --- | --- | --- |
| **Effect** | **Num DF** | **Den DF** | **F Value** | **Pr > F** |
| **period2** | 1 | 10 | 3.37 | 0.0963 |
| **TRT** | 1 | 10 | 2.28 | 0.1622 |
| **day** | 4 | 88 | 5.42 | 0.0006 |
| **day*TRT** | 4 | 88 | 3.11 | 0.0192 |

| The SAS System |
| --- |
| Mean separation for TOTALkg |
| Differences of Least Squares Means |

Effect=period2 bygroup=1

| **Obs** | **ADJUSTMENT** | **adjp** | **day** | **TRT** | **period2** | **_day** | **_TRT** | **_period2** | **Estimate** | **StdErr** | **DF** | **tValue** | **Probt** |
| --- | --- | --- | --- | --- | --- | --- | --- | --- | --- | --- | --- | --- | --- |
| **1** | LSD(P<.05) | 0.096295 | _ |  | 1 | _ |  | 2 | 1.5048 | 0.8198 | 10 | 1.84 | 0.0963 |

Effect=TRT bygroup=2

| **Obs** | **ADJUSTMENT** | **adjp** | **day** | **TRT** | **period2** | **_day** | **_TRT** | **_period2** | **Estimate** | **StdErr** | **DF** | **tValue** | **Probt** |
| --- | --- | --- | --- | --- | --- | --- | --- | --- | --- | --- | --- | --- | --- |
| **2** | LSD(P<.05) | 0.16220 | _ | Lying | _ | _ | Sleep | _ | -1.2372 | 0.8198 | 10 | -1.51 | 0.1622 |

Effect=day bygroup=3

| **Obs** | **ADJUSTMENT** | **adjp** | **day** | **TRT** | **period2** | **_day** | **_TRT** | **_period2** | **Estimate** | **StdErr** | **DF** | **tValue** | **Probt** |
| --- | --- | --- | --- | --- | --- | --- | --- | --- | --- | --- | --- | --- | --- |
| **3** | LSD(P<.05) | 0.00721 | 0 |  | _ | 2 |  | _ | 1.8059 | 0.6564 | 88 | 2.75 | 0.0072 |
| **4** | LSD(P<.05) | 0.00432 | 0 |  | _ | 3 |  | _ | 1.9230 | 0.6564 | 88 | 2.93 | 0.0043 |
| **5** | LSD(P<.05) | 0.95535 | 0 |  | _ | 4 |  | _ | 0.03685 | 0.6564 | 88 | 0.06 | 0.9554 |
| **6** | LSD(P<.05) | 0.63694 | 0 |  | _ | 5 |  | _ | -0.3109 | 0.6564 | 88 | -0.47 | 0.6369 |
| **7** | LSD(P<.05) | 0.85873 | 2 |  | _ | 3 |  | _ | 0.1172 | 0.6564 | 88 | 0.18 | 0.8587 |
| **8** | LSD(P<.05) | 0.00843 | 2 |  | _ | 4 |  | _ | -1.7690 | 0.6564 | 88 | -2.69 | 0.0084 |
| **9** | LSD(P<.05) | 0.00177 | 2 |  | _ | 5 |  | _ | -2.1168 | 0.6564 | 88 | -3.22 | 0.0018 |
| **10** | LSD(P<.05) | 0.00509 | 3 |  | _ | 4 |  | _ | -1.8862 | 0.6564 | 88 | -2.87 | 0.0051 |
| **11** | LSD(P<.05) | 0.00100 | 3 |  | _ | 5 |  | _ | -2.2339 | 0.6564 | 88 | -3.40 | 0.0010 |
| **12** | LSD(P<.05) | 0.59761 | 4 |  | _ | 5 |  | _ | -0.3478 | 0.6564 | 88 | -0.53 | 0.5976 |

Effect=day*TRT bygroup=4

| **Obs** | **ADJUSTMENT** | **adjp** | **day** | **TRT** | **period2** | **_day** | **_TRT** | **_period2** | **Estimate** | **StdErr** | **DF** | **tValue** | **Probt** |
| --- | --- | --- | --- | --- | --- | --- | --- | --- | --- | --- | --- | --- | --- |
| **13** | LSD(P<.05) | 0.44092 | 0 | Lying | _ | 0 | Sleep | _ | -0.9091 | 1.1668 | 36.66 | -0.78 | 0.4409 |
| **14** | LSD(P<.05) | 0.00111 | 0 | Lying | _ | 2 | Lying | _ | 3.1298 | 0.9283 | 88 | 3.37 | 0.0011 |
| **15** | LSD(P<.05) | 0.71642 | 0 | Lying | _ | 2 | Sleep | _ | -0.4271 | 1.1668 | 36.66 | -0.37 | 0.7164 |
| **16** | LSD(P<.05) | 0.02354 | 0 | Lying | _ | 3 | Lying | _ | 2.1394 | 0.9283 | 88 | 2.30 | 0.0235 |
| **17** | LSD(P<.05) | 0.49857 | 0 | Lying | _ | 3 | Sleep | _ | 0.7976 | 1.1668 | 36.66 | 0.68 | 0.4986 |
| **18** | LSD(P<.05) | 0.75462 | 0 | Lying | _ | 4 | Lying | _ | 0.2911 | 0.9283 | 88 | 0.31 | 0.7546 |
| **19** | LSD(P<.05) | 0.34069 | 0 | Lying | _ | 4 | Sleep | _ | -1.1264 | 1.1668 | 36.66 | -0.97 | 0.3407 |
| **20** | LSD(P<.05) | 0.16974 | 0 | Lying | _ | 5 | Lying | _ | -1.2852 | 0.9283 | 88 | -1.38 | 0.1697 |
| **21** | LSD(P<.05) | 0.83439 | 0 | Lying | _ | 5 | Sleep | _ | -0.2457 | 1.1668 | 36.66 | -0.21 | 0.8344 |
| **22** | LSD(P<.05) | 0.00138 | 0 | Sleep | _ | 2 | Lying | _ | 4.0389 | 1.1668 | 36.66 | 3.46 | 0.0014 |
| **23** | LSD(P<.05) | 0.60496 | 0 | Sleep | _ | 2 | Sleep | _ | 0.4819 | 0.9283 | 88 | 0.52 | 0.6050 |
| **24** | LSD(P<.05) | 0.01295 | 0 | Sleep | _ | 3 | Lying | _ | 3.0485 | 1.1668 | 36.66 | 2.61 | 0.0129 |
| **25** | LSD(P<.05) | 0.06938 | 0 | Sleep | _ | 3 | Sleep | _ | 1.7066 | 0.9283 | 88 | 1.84 | 0.0694 |
| **26** | LSD(P<.05) | 0.31044 | 0 | Sleep | _ | 4 | Lying | _ | 1.2001 | 1.1668 | 36.66 | 1.03 | 0.3104 |
| **27** | LSD(P<.05) | 0.81543 | 0 | Sleep | _ | 4 | Sleep | _ | -0.2173 | 0.9283 | 88 | -0.23 | 0.8154 |
| **28** | LSD(P<.05) | 0.74903 | 0 | Sleep | _ | 5 | Lying | _ | -0.3761 | 1.1668 | 36.66 | -0.32 | 0.7490 |
| **29** | LSD(P<.05) | 0.47675 | 0 | Sleep | _ | 5 | Sleep | _ | 0.6634 | 0.9283 | 88 | 0.71 | 0.4768 |
| **30** | LSD(P<.05) | 0.00425 | 2 | Lying | _ | 2 | Sleep | _ | -3.5569 | 1.1668 | 36.66 | -3.05 | 0.0043 |
| **31** | LSD(P<.05) | 0.28898 | 2 | Lying | _ | 3 | Lying | _ | -0.9903 | 0.9283 | 88 | -1.07 | 0.2890 |
| **32** | LSD(P<.05) | 0.05309 | 2 | Lying | _ | 3 | Sleep | _ | -2.3322 | 1.1668 | 36.66 | -2.00 | 0.0531 |
| **33** | LSD(P<.05) | 0.00295 | 2 | Lying | _ | 4 | Lying | _ | -2.8387 | 0.9283 | 88 | -3.06 | 0.0030 |
| **34** | LSD(P<.05) | 0.00082 | 2 | Lying | _ | 4 | Sleep | _ | -4.2562 | 1.1668 | 36.66 | -3.65 | 0.0008 |
| **35** | LSD(P<.05) | 0.00001 | 2 | Lying | _ | 5 | Lying | _ | -4.4150 | 0.9283 | 88 | -4.76 | <.0001 |
| **36** | LSD(P<.05) | 0.00639 | 2 | Lying | _ | 5 | Sleep | _ | -3.3755 | 1.1668 | 36.66 | -2.89 | 0.0064 |
| **37** | LSD(P<.05) | 0.03422 | 2 | Sleep | _ | 3 | Lying | _ | 2.5666 | 1.1668 | 36.66 | 2.20 | 0.0342 |
| **38** | LSD(P<.05) | 0.19051 | 2 | Sleep | _ | 3 | Sleep | _ | 1.2247 | 0.9283 | 88 | 1.32 | 0.1905 |
| **39** | LSD(P<.05) | 0.54203 | 2 | Sleep | _ | 4 | Lying | _ | 0.7182 | 1.1668 | 36.66 | 0.62 | 0.5420 |
| **40** | LSD(P<.05) | 0.45329 | 2 | Sleep | _ | 4 | Sleep | _ | -0.6993 | 0.9283 | 88 | -0.75 | 0.4533 |
| **41** | LSD(P<.05) | 0.46680 | 2 | Sleep | _ | 5 | Lying | _ | -0.8580 | 1.1668 | 36.66 | -0.74 | 0.4668 |
| **42** | LSD(P<.05) | 0.84550 | 2 | Sleep | _ | 5 | Sleep | _ | 0.1814 | 0.9283 | 88 | 0.20 | 0.8455 |
| **43** | LSD(P<.05) | 0.25758 | 3 | Lying | _ | 3 | Sleep | _ | -1.3419 | 1.1668 | 36.66 | -1.15 | 0.2576 |
| **44** | LSD(P<.05) | 0.04957 | 3 | Lying | _ | 4 | Lying | _ | -1.8484 | 0.9283 | 88 | -1.99 | 0.0496 |
| **45** | LSD(P<.05) | 0.00813 | 3 | Lying | _ | 4 | Sleep | _ | -3.2659 | 1.1668 | 36.66 | -2.80 | 0.0081 |
| **46** | LSD(P<.05) | 0.00039 | 3 | Lying | _ | 5 | Lying | _ | -3.4246 | 0.9283 | 88 | -3.69 | 0.0004 |
| **47** | LSD(P<.05) | 0.04818 | 3 | Lying | _ | 5 | Sleep | _ | -2.3851 | 1.1668 | 36.66 | -2.04 | 0.0482 |
| **48** | LSD(P<.05) | 0.66677 | 3 | Sleep | _ | 4 | Lying | _ | -0.5065 | 1.1668 | 36.66 | -0.43 | 0.6668 |
| **49** | LSD(P<.05) | 0.04114 | 3 | Sleep | _ | 4 | Sleep | _ | -1.9240 | 0.9283 | 88 | -2.07 | 0.0411 |
| **50** | LSD(P<.05) | 0.08255 | 3 | Sleep | _ | 5 | Lying | _ | -2.0827 | 1.1668 | 36.66 | -1.78 | 0.0825 |
| **51** | LSD(P<.05) | 0.26415 | 3 | Sleep | _ | 5 | Sleep | _ | -1.0433 | 0.9283 | 88 | -1.12 | 0.2642 |
| **52** | LSD(P<.05) | 0.23221 | 4 | Lying | _ | 4 | Sleep | _ | -1.4175 | 1.1668 | 36.66 | -1.21 | 0.2322 |
| **53** | LSD(P<.05) | 0.09306 | 4 | Lying | _ | 5 | Lying | _ | -1.5762 | 0.9283 | 88 | -1.70 | 0.0931 |
| **54** | LSD(P<.05) | 0.64823 | 4 | Lying | _ | 5 | Sleep | _ | -0.5368 | 1.1668 | 36.66 | -0.46 | 0.6482 |
| **55** | LSD(P<.05) | 0.89252 | 4 | Sleep | _ | 5 | Lying | _ | -0.1588 | 1.1668 | 36.66 | -0.14 | 0.8925 |
| **56** | LSD(P<.05) | 0.34536 | 4 | Sleep | _ | 5 | Sleep | _ | 0.8807 | 0.9283 | 88 | 0.95 | 0.3454 |
| **57** | LSD(P<.05) | 0.37882 | 5 | Lying | _ | 5 | Sleep | _ | 1.0395 | 1.1668 | 36.66 | 0.89 | 0.3788 |

| The SAS System |
| --- |
| Mean separation for TOTALkg |
| Differences of Least Squares Means |

| **Set** | **Average Sig Diff Value** | **Minimum Sig Diff Value** | **Maximum Sig Diff Value** |
| --- | --- | --- | --- |
| 1 | 1.82662 | 1.82662 | 1.82662 |
| 2 | 1.82662 | 1.82662 | 1.82662 |
| 3 | 1.30451 | 1.30451 | 1.30451 |
| 4 | 2.13381 | 1.84485 | 2.36497 |

| The SAS System |
| --- |
| PDMIX Mean Separation for TOTALkg |

Effect=period2 Method=LSD(P<.05) Set=1

| **Obs** | **day** | **TRT** | **period2** | **Estimate** | **Standard Error** | **Mean** | **Standard Error of Mean** | **Letter Group** |
| --- | --- | --- | --- | --- | --- | --- | --- | --- |
| **1** | _ |  | 1 | 35.4388 | 2.4102 | 35.4388 | 2.4102 | A |
| **2** | _ |  | 2 | 33.9340 | 2.4102 | 33.9340 | 2.4102 | A |

Effect=TRT Method=LSD(P<.05) Set=2

| **Obs** | **day** | **TRT** | **period2** | **Estimate** | **Standard Error** | **Mean** | **Standard Error of Mean** | **Letter Group** |
| --- | --- | --- | --- | --- | --- | --- | --- | --- |
| **3** | _ | Lying | _ | 34.0678 | 2.4102 | 34.0678 | 2.4102 | A |
| **4** | _ | Sleep | _ | 35.3050 | 2.4102 | 35.3050 | 2.4102 | A |

Effect=day Method=LSD(P<.05) Set=3

| **Obs** | **day** | **TRT** | **period2** | **Estimate** | **Standard Error** | **Mean** | **Standard Error of Mean** | **Letter Group** |
| --- | --- | --- | --- | --- | --- | --- | --- | --- |
| **5** | 0 |  | _ | 35.3773 | 2.4111 | 35.3773 | 2.4111 | A |
| **6** | 2 |  | _ | 33.5715 | 2.4111 | 33.5715 | 2.4111 | B |
| **7** | 3 |  | _ | 33.4543 | 2.4111 | 33.4543 | 2.4111 | B |
| **8** | 4 |  | _ | 35.3405 | 2.4111 | 35.3405 | 2.4111 | A |
| **9** | 5 |  | _ | 35.6882 | 2.4111 | 35.6882 | 2.4111 | A |

Effect=day*TRT Method=LSD(P<.05) Set=4

| **Obs** | **day** | **TRT** | **period2** | **Estimate** | **Standard Error** | **Mean** | **Standard Error of Mean** | **Letter Group** |
| --- | --- | --- | --- | --- | --- | --- | --- | --- |
| **10** | 0 | Lying | _ | 34.9228 | 2.4807 | 34.9228 | 2.4807 | AB |
| **11** | 0 | Sleep | _ | 35.8319 | 2.4807 | 35.8319 | 2.4807 | AB |
| **12** | 2 | Lying | _ | 31.7930 | 2.4807 | 31.7930 | 2.4807 | C |
| **13** | 2 | Sleep | _ | 35.3499 | 2.4807 | 35.3499 | 2.4807 | AB |
| **14** | 3 | Lying | _ | 32.7834 | 2.4807 | 32.7834 | 2.4807 | C |
| **15** | 3 | Sleep | _ | 34.1252 | 2.4807 | 34.1252 | 2.4807 | BC |
| **16** | 4 | Lying | _ | 34.6317 | 2.4807 | 34.6317 | 2.4807 | AB |
| **17** | 4 | Sleep | _ | 36.0492 | 2.4807 | 36.0492 | 2.4807 | A |
| **18** | 5 | Lying | _ | 36.2080 | 2.4807 | 36.2080 | 2.4807 | AB |
| **19** | 5 | Sleep | _ | 35.1685 | 2.4807 | 35.1685 | 2.4807 | AB |

The UNIVARIATE Procedure

Variable: residual (Residual (Mu scale))

| **Tests for Normality** | | | | |
| --- | --- | --- | --- | --- |
| **Test** | **Statistic** | | **p Value** | |
| **Shapiro-Wilk** | **W** | 0.901515 | **Pr < W** | <0.0001 |
| **Kolmogorov-Smirnov** | **D** | 0.099231 | **Pr > D** | <0.0100 |
| **Cramer-von Mises** | **W-Sq** | 0.26129 | **Pr > W-Sq** | <0.0050 |
| **Anderson-Darling** | **A-Sq** | 1.778662 | **Pr > A-Sq** | <0.0050 |

**Milk production with baseline being mattress**

| The SAS System |
| --- |
| GLIMMix ANOVA for TOTALkg |

The GLIMMIX Procedure

| **Class Level Information** | | |
| --- | --- | --- |
| **Class** | **Levels** | **Values** |
| **COW** | 12 | 4444 4479 4481 4484 4486 4490 4507 4512 4518 6302 6725 13162 |
| **day** | 5 | 0 2 3 4 5 |
| **TRT** | 2 | Lying Sleep |
| **period2** | 2 | 1 2 |

| **Number of Observations Read** | 120 |
| --- | --- |
| **Number of Observations Used** | 120 |

| Convergence criterion (GCONV=1E-8) satisfied. |
| --- |

| **Fit Statistics** | |
| --- | --- |
| **-2 Res Log Likelihood** | 597.39 |
| **AIC (smaller is better)** | 605.39 |
| **AICC (smaller is better)** | 605.77 |
| **BIC (smaller is better)** | 607.33 |
| **CAIC (smaller is better)** | 611.33 |
| **HQIC (smaller is better)** | 604.67 |
| **Generalized Chi-Square** | 108.74 |
| **Gener. Chi-Square / DF** | 1.00 |

| **Covariance Parameter Estimates** | | |
| --- | --- | --- |
| **Cov Parm** | **Estimate** | **Standard Error** |
| **COW** | 59.9581 | 25.9711 |
| **COW*TRT*period2** | 0.5419 | 0.8622 |
| **COW*day*TRT*period2** | 5.6658 | 1.0045 |
| **Residual (VC)** | 0.9976 | . |

| **Type III Tests of Fixed Effects** | | | | |
| --- | --- | --- | --- | --- |
| **Effect** | **Num DF** | **Den DF** | **F Value** | **Pr > F** |
| **period2** | 1 | 9.998 | 7.97 | 0.0181 |
| **TRT** | 1 | 9.998 | 6.32 | 0.0307 |
| **day** | 4 | 88 | 3.80 | 0.0067 |
| **day*TRT** | 4 | 88 | 2.42 | 0.0547 |

| The SAS System |
| --- |
| Mean separation for TOTALkg |
| Differences of Least Squares Means |

Effect=period2 bygroup=1

| **Obs** | **ADJUSTMENT** | **adjp** | **day** | **TRT** | **period2** | **_day** | **_TRT** | **_period2** | **Estimate** | **StdErr** | **DF** | **tValue** | **Probt** |
| --- | --- | --- | --- | --- | --- | --- | --- | --- | --- | --- | --- | --- | --- |
| **1** | LSD(P<.05) | 0.018082 | _ |  | 1 | _ |  | 2 | 1.5777 | 0.5590 | 9.998 | 2.82 | 0.0181 |

Effect=TRT bygroup=2

| **Obs** | **ADJUSTMENT** | **adjp** | **day** | **TRT** | **period2** | **_day** | **_TRT** | **_period2** | **Estimate** | **StdErr** | **DF** | **tValue** | **Probt** |
| --- | --- | --- | --- | --- | --- | --- | --- | --- | --- | --- | --- | --- | --- |
| **2** | LSD(P<.05) | 0.030691 | _ | Lying | _ | _ | Sleep | _ | -1.4054 | 0.5590 | 9.998 | -2.51 | 0.0307 |

Effect=day bygroup=3

| **Obs** | **ADJUSTMENT** | **adjp** | **day** | **TRT** | **period2** | **_day** | **_TRT** | **_period2** | **Estimate** | **StdErr** | **DF** | **tValue** | **Probt** |
| --- | --- | --- | --- | --- | --- | --- | --- | --- | --- | --- | --- | --- | --- |
| **3** | LSD(P<.05) | 0.49368 | 0 |  | _ | 2 |  | _ | 0.5122 | 0.7452 | 88 | 0.69 | 0.4937 |
| **4** | LSD(P<.05) | 0.40063 | 0 |  | _ | 3 |  | _ | 0.6294 | 0.7452 | 88 | 0.84 | 0.4006 |
| **5** | LSD(P<.05) | 0.09522 | 0 |  | _ | 4 |  | _ | -1.2568 | 0.7452 | 88 | -1.69 | 0.0952 |
| **6** | LSD(P<.05) | 0.03403 | 0 |  | _ | 5 |  | _ | -1.6046 | 0.7452 | 88 | -2.15 | 0.0340 |
| **7** | LSD(P<.05) | 0.87541 | 2 |  | _ | 3 |  | _ | 0.1172 | 0.7452 | 88 | 0.16 | 0.8754 |
| **8** | LSD(P<.05) | 0.01977 | 2 |  | _ | 4 |  | _ | -1.7690 | 0.7452 | 88 | -2.37 | 0.0198 |
| **9** | LSD(P<.05) | 0.00559 | 2 |  | _ | 5 |  | _ | -2.1168 | 0.7452 | 88 | -2.84 | 0.0056 |
| **10** | LSD(P<.05) | 0.01314 | 3 |  | _ | 4 |  | _ | -1.8862 | 0.7452 | 88 | -2.53 | 0.0131 |
| **11** | LSD(P<.05) | 0.00353 | 3 |  | _ | 5 |  | _ | -2.2339 | 0.7452 | 88 | -3.00 | 0.0035 |
| **12** | LSD(P<.05) | 0.64188 | 4 |  | _ | 5 |  | _ | -0.3478 | 0.7452 | 88 | -0.47 | 0.6419 |

Effect=day*TRT bygroup=4

| **Obs** | **ADJUSTMENT** | **adjp** | **day** | **TRT** | **period2** | **_day** | **_TRT** | **_period2** | **Estimate** | **StdErr** | **DF** | **tValue** | **Probt** |
| --- | --- | --- | --- | --- | --- | --- | --- | --- | --- | --- | --- | --- | --- |
| **13** | LSD(P<.05) | 0.11435 | 0 | Lying | _ | 0 | Sleep | _ | -1.7501 | 1.0958 | 76.98 | -1.60 | 0.1144 |
| **14** | LSD(P<.05) | 0.18264 | 0 | Lying | _ | 2 | Lying | _ | 1.4156 | 1.0538 | 88 | 1.34 | 0.1826 |
| **15** | LSD(P<.05) | 0.05433 | 0 | Lying | _ | 2 | Sleep | _ | -2.1413 | 1.0958 | 76.98 | -1.95 | 0.0543 |
| **16** | LSD(P<.05) | 0.68754 | 0 | Lying | _ | 3 | Lying | _ | 0.4252 | 1.0538 | 88 | 0.40 | 0.6875 |
| **17** | LSD(P<.05) | 0.40548 | 0 | Lying | _ | 3 | Sleep | _ | -0.9166 | 1.0958 | 76.98 | -0.84 | 0.4055 |
| **18** | LSD(P<.05) | 0.18033 | 0 | Lying | _ | 4 | Lying | _ | -1.4231 | 1.0538 | 88 | -1.35 | 0.1803 |
| **19** | LSD(P<.05) | 0.01141 | 0 | Lying | _ | 4 | Sleep | _ | -2.8406 | 1.0958 | 76.98 | -2.59 | 0.0114 |
| **20** | LSD(P<.05) | 0.00550 | 0 | Lying | _ | 5 | Lying | _ | -2.9994 | 1.0538 | 88 | -2.85 | 0.0055 |
| **21** | LSD(P<.05) | 0.07763 | 0 | Lying | _ | 5 | Sleep | _ | -1.9599 | 1.0958 | 76.98 | -1.79 | 0.0776 |
| **22** | LSD(P<.05) | 0.00502 | 0 | Sleep | _ | 2 | Lying | _ | 3.1657 | 1.0958 | 76.98 | 2.89 | 0.0050 |
| **23** | LSD(P<.05) | 0.71135 | 0 | Sleep | _ | 2 | Sleep | _ | -0.3912 | 1.0538 | 88 | -0.37 | 0.7114 |
| **24** | LSD(P<.05) | 0.05070 | 0 | Sleep | _ | 3 | Lying | _ | 2.1754 | 1.0958 | 76.98 | 1.99 | 0.0507 |
| **25** | LSD(P<.05) | 0.43113 | 0 | Sleep | _ | 3 | Sleep | _ | 0.8335 | 1.0538 | 88 | 0.79 | 0.4311 |
| **26** | LSD(P<.05) | 0.76623 | 0 | Sleep | _ | 4 | Lying | _ | 0.3270 | 1.0958 | 76.98 | 0.30 | 0.7662 |
| **27** | LSD(P<.05) | 0.30359 | 0 | Sleep | _ | 4 | Sleep | _ | -1.0905 | 1.0538 | 88 | -1.03 | 0.3036 |
| **28** | LSD(P<.05) | 0.25782 | 0 | Sleep | _ | 5 | Lying | _ | -1.2493 | 1.0958 | 76.98 | -1.14 | 0.2578 |
| **29** | LSD(P<.05) | 0.84267 | 0 | Sleep | _ | 5 | Sleep | _ | -0.2098 | 1.0538 | 88 | -0.20 | 0.8427 |
| **30** | LSD(P<.05) | 0.00173 | 2 | Lying | _ | 2 | Sleep | _ | -3.5569 | 1.0958 | 76.98 | -3.25 | 0.0017 |
| **31** | LSD(P<.05) | 0.34992 | 2 | Lying | _ | 3 | Lying | _ | -0.9903 | 1.0538 | 88 | -0.94 | 0.3499 |
| **32** | LSD(P<.05) | 0.03652 | 2 | Lying | _ | 3 | Sleep | _ | -2.3322 | 1.0958 | 76.98 | -2.13 | 0.0365 |
| **33** | LSD(P<.05) | 0.00846 | 2 | Lying | _ | 4 | Lying | _ | -2.8387 | 1.0538 | 88 | -2.69 | 0.0085 |
| **34** | LSD(P<.05) | 0.00022 | 2 | Lying | _ | 4 | Sleep | _ | -4.2562 | 1.0958 | 76.98 | -3.88 | 0.0002 |
| **35** | LSD(P<.05) | 0.00007 | 2 | Lying | _ | 5 | Lying | _ | -4.4150 | 1.0538 | 88 | -4.19 | <.0001 |
| **36** | LSD(P<.05) | 0.00287 | 2 | Lying | _ | 5 | Sleep | _ | -3.3755 | 1.0958 | 76.98 | -3.08 | 0.0029 |
| **37** | LSD(P<.05) | 0.02176 | 2 | Sleep | _ | 3 | Lying | _ | 2.5666 | 1.0958 | 76.98 | 2.34 | 0.0218 |
| **38** | LSD(P<.05) | 0.24832 | 2 | Sleep | _ | 3 | Sleep | _ | 1.2247 | 1.0538 | 88 | 1.16 | 0.2483 |
| **39** | LSD(P<.05) | 0.51418 | 2 | Sleep | _ | 4 | Lying | _ | 0.7182 | 1.0958 | 76.98 | 0.66 | 0.5142 |
| **40** | LSD(P<.05) | 0.50870 | 2 | Sleep | _ | 4 | Sleep | _ | -0.6993 | 1.0538 | 88 | -0.66 | 0.5087 |
| **41** | LSD(P<.05) | 0.43603 | 2 | Sleep | _ | 5 | Lying | _ | -0.8580 | 1.0958 | 76.98 | -0.78 | 0.4360 |
| **42** | LSD(P<.05) | 0.86370 | 2 | Sleep | _ | 5 | Sleep | _ | 0.1814 | 1.0538 | 88 | 0.17 | 0.8637 |
| **43** | LSD(P<.05) | 0.22449 | 3 | Lying | _ | 3 | Sleep | _ | -1.3419 | 1.0958 | 76.98 | -1.22 | 0.2245 |
| **44** | LSD(P<.05) | 0.08292 | 3 | Lying | _ | 4 | Lying | _ | -1.8484 | 1.0538 | 88 | -1.75 | 0.0829 |
| **45** | LSD(P<.05) | 0.00385 | 3 | Lying | _ | 4 | Sleep | _ | -3.2659 | 1.0958 | 76.98 | -2.98 | 0.0039 |
| **46** | LSD(P<.05) | 0.00164 | 3 | Lying | _ | 5 | Lying | _ | -3.4246 | 1.0538 | 88 | -3.25 | 0.0016 |
| **47** | LSD(P<.05) | 0.03258 | 3 | Lying | _ | 5 | Sleep | _ | -2.3851 | 1.0958 | 76.98 | -2.18 | 0.0326 |
| **48** | LSD(P<.05) | 0.64523 | 3 | Sleep | _ | 4 | Lying | _ | -0.5065 | 1.0958 | 76.98 | -0.46 | 0.6452 |
| **49** | LSD(P<.05) | 0.07129 | 3 | Sleep | _ | 4 | Sleep | _ | -1.9240 | 1.0538 | 88 | -1.83 | 0.0713 |
| **50** | LSD(P<.05) | 0.06110 | 3 | Sleep | _ | 5 | Lying | _ | -2.0827 | 1.0958 | 76.98 | -1.90 | 0.0611 |
| **51** | LSD(P<.05) | 0.32490 | 3 | Sleep | _ | 5 | Sleep | _ | -1.0433 | 1.0538 | 88 | -0.99 | 0.3249 |
| **52** | LSD(P<.05) | 0.19971 | 4 | Lying | _ | 4 | Sleep | _ | -1.4175 | 1.0958 | 76.98 | -1.29 | 0.1997 |
| **53** | LSD(P<.05) | 0.13830 | 4 | Lying | _ | 5 | Lying | _ | -1.5762 | 1.0538 | 88 | -1.50 | 0.1383 |
| **54** | LSD(P<.05) | 0.62566 | 4 | Lying | _ | 5 | Sleep | _ | -0.5368 | 1.0958 | 76.98 | -0.49 | 0.6257 |
| **55** | LSD(P<.05) | 0.88519 | 4 | Sleep | _ | 5 | Lying | _ | -0.1588 | 1.0958 | 76.98 | -0.14 | 0.8852 |
| **56** | LSD(P<.05) | 0.40557 | 4 | Sleep | _ | 5 | Sleep | _ | 0.8807 | 1.0538 | 88 | 0.84 | 0.4056 |
| **57** | LSD(P<.05) | 0.34581 | 5 | Lying | _ | 5 | Sleep | _ | 1.0395 | 1.0958 | 76.98 | 0.95 | 0.3458 |

| The SAS System |
| --- |
| Mean separation for TOTALkg |
| Differences of Least Squares Means |

| **Set** | **Average Sig Diff Value** | **Minimum Sig Diff Value** | **Maximum Sig Diff Value** |
| --- | --- | --- | --- |
| 1 | 1.24547 | 1.24547 | 1.24547 |
| 2 | 1.24547 | 1.24547 | 1.24547 |
| 3 | 1.48087 | 1.48087 | 1.48087 |
| 4 | 2.14307 | 2.09426 | 2.18212 |

| The SAS System |
| --- |
| PDMIX Mean Separation for TOTALkg |

Effect=period2 Method=LSD(P<.05) Set=1

| **Obs** | **day** | **TRT** | **period2** | **Estimate** | **Standard Error** | **Mean** | **Standard Error of Mean** | **Letter Group** |
| --- | --- | --- | --- | --- | --- | --- | --- | --- |
| **1** | _ |  | 1 | 35.2165 | 2.2700 | 35.2165 | 2.2700 | A |
| **2** | _ |  | 2 | 33.6388 | 2.2700 | 33.6388 | 2.2700 | B |

Effect=TRT Method=LSD(P<.05) Set=2

| **Obs** | **day** | **TRT** | **period2** | **Estimate** | **Standard Error** | **Mean** | **Standard Error of Mean** | **Letter Group** |
| --- | --- | --- | --- | --- | --- | --- | --- | --- |
| **3** | _ | Lying | _ | 33.7249 | 2.2700 | 33.7249 | 2.2700 | B |
| **4** | _ | Sleep | _ | 35.1303 | 2.2700 | 35.1303 | 2.2700 | A |

Effect=day Method=LSD(P<.05) Set=3

| **Obs** | **day** | **TRT** | **period2** | **Estimate** | **Standard Error** | **Mean** | **Standard Error of Mean** | **Letter Group** |
| --- | --- | --- | --- | --- | --- | --- | --- | --- |
| **5** | 0 |  | _ | 34.0837 | 2.3015 | 34.0837 | 2.3015 | BC |
| **6** | 2 |  | _ | 33.5715 | 2.3015 | 33.5715 | 2.3015 | C |
| **7** | 3 |  | _ | 33.4543 | 2.3015 | 33.4543 | 2.3015 | C |
| **8** | 4 |  | _ | 35.3405 | 2.3015 | 35.3405 | 2.3015 | AB |
| **9** | 5 |  | _ | 35.6882 | 2.3015 | 35.6882 | 2.3015 | A |

Effect=day*TRT Method=LSD(P<.05) Set=4

| **Obs** | **day** | **TRT** | **period2** | **Estimate** | **Standard Error** | **Mean** | **Standard Error of Mean** | **Letter Group** |
| --- | --- | --- | --- | --- | --- | --- | --- | --- |
| **10** | 0 | Lying | _ | 33.2086 | 2.3658 | 33.2086 | 2.3658 | BCD |
| **11** | 0 | Sleep | _ | 34.9587 | 2.3658 | 34.9587 | 2.3658 | ABC |
| **12** | 2 | Lying | _ | 31.7930 | 2.3658 | 31.7930 | 2.3658 | D |
| **13** | 2 | Sleep | _ | 35.3499 | 2.3658 | 35.3499 | 2.3658 | AB |
| **14** | 3 | Lying | _ | 32.7834 | 2.3658 | 32.7834 | 2.3658 | CD |
| **15** | 3 | Sleep | _ | 34.1252 | 2.3658 | 34.1252 | 2.3658 | ABC |
| **16** | 4 | Lying | _ | 34.6317 | 2.3658 | 34.6317 | 2.3658 | ABC |
| **17** | 4 | Sleep | _ | 36.0492 | 2.3658 | 36.0492 | 2.3658 | A |
| **18** | 5 | Lying | _ | 36.2080 | 2.3658 | 36.2080 | 2.3658 | A |
| **19** | 5 | Sleep | _ | 35.1685 | 2.3658 | 35.1685 | 2.3658 | AB |
