# Supplementary material for: Effects of acute lying and sleep deprivation on the behavior of lactating dairy cows
Source: PLoS One. 2019 Aug 28;14(8):e0212823. doi: 10.1371/journal.pone.0212823 (PMC6713338; doi:10.1371/journal.pone.0212823)
Supplement: S7 File — Supplemental data from SAS model to support conclusions drawn on the effects of treatment on daily milk protein from cows. (DOCX) [file pone.0212823.s009.docx]

protein: looking at it by dat and day*trt interaction. Could not look at specific times and day interaction because not equal samples taken across baseline, trt or Recov

| The SAS System |
| --- |
| GLIMMix ANOVA for protein |

The GLIMMIX Procedure

| **Class Level Information** | | |
| --- | --- | --- |
| **Class** | **Levels** | **Values** |
| **Cow_ID** | 12 | 4444 4479 4481 4484 4486 4490 4507 4512 4518 6302 6725 13162 |
| **Day** | 3 | 0 1 2 |
| **Trt** | 2 | Lying Sleep |
| **Period** | 2 | 1 2 |

| **Number of Observations Read** | 70 |
| --- | --- |
| **Number of Observations Used** | 63 |

| Convergence criterion (GCONV=1E-8) satisfied. |
| --- |

| **Fit Statistics** | |
| --- | --- |
| **-2 Res Log Likelihood** | -161.76 |
| **AIC (smaller is better)** | -153.76 |
| **AICC (smaller is better)** | -152.97 |
| **BIC (smaller is better)** | -151.82 |
| **CAIC (smaller is better)** | -147.82 |
| **HQIC (smaller is better)** | -154.48 |
| **Generalized Chi-Square** | 0.04 |
| **Gener. Chi-Square / DF** | 0.00 |

| **Covariance Parameter Estimates** | | | |
| --- | --- | --- | --- |
| **Cov Parm** | **Subject** | **Estimate** | **Standard Error** |
| **Cow_ID** |  | 0.01483 | 0.006576 |
| **Cow_ID*Trt*Period** |  | 0.000787 | 0.000457 |
| **AR(1)** | **Cow_ID*Trt*Period** | -0.4995 | 0.2408 |
| **Residual** |  | 0.000717 | 0.000170 |

| **Type III Tests of Fixed Effects** | | | | |
| --- | --- | --- | --- | --- |
| **Effect** | **Num DF** | **Den DF** | **F Value** | **Pr > F** |
| **Period** | 1 | 8.303 | 4.76 | 0.0594 |
| **Trt** | 1 | 8.772 | 7.91 | 0.0208 |
| **Day** | 2 | 21.68 | 2.70 | 0.0897 |
| **Day*Trt** | 2 | 21.5 | 4.00 | 0.0333 |

| The SAS System |
| --- |
| Mean separation for log protein |
| Differences of Least Squares Means |

Effect=Period bygroup=1

| **Obs** | **ADJUSTMENT** | **adjp** | **Day** | **Trt** | **Period** | **_Day** | **_Trt** | **_Period** | **Estimate** | **StdErr** | **DF** | **tValue** | **Probt** |
| --- | --- | --- | --- | --- | --- | --- | --- | --- | --- | --- | --- | --- | --- |
| **1** | LSD(P<.05) | 0.059440 | _ |  | 1 | _ |  | 2 | -0.02901 | 0.01330 | 8.303 | -2.18 | 0.0594 |

Effect=Trt bygroup=2

| **Obs** | **ADJUSTMENT** | **adjp** | **Day** | **Trt** | **Period** | **_Day** | **_Trt** | **_Period** | **Estimate** | **StdErr** | **DF** | **tValue** | **Probt** |
| --- | --- | --- | --- | --- | --- | --- | --- | --- | --- | --- | --- | --- | --- |
| **2** | LSD(P<.05) | 0.020795 | _ | Lying | _ | _ | Sleep | _ | 0.03786 | 0.01346 | 8.772 | 2.81 | 0.0208 |

Effect=Day bygroup=3

| **Obs** | **ADJUSTMENT** | **adjp** | **Day** | **Trt** | **Period** | **_Day** | **_Trt** | **_Period** | **Estimate** | **StdErr** | **DF** | **tValue** | **Probt** |
| --- | --- | --- | --- | --- | --- | --- | --- | --- | --- | --- | --- | --- | --- |
| **3** | LSD(P<.05) | 0.28649 | 0 |  | _ | 1 |  | _ | -0.01077 | 0.009873 | 23.26 | -1.09 | 0.2865 |
| **4** | LSD(P<.05) | 0.03362 | 0 |  | _ | 2 |  | _ | -0.01874 | 0.007995 | 14.69 | -2.34 | 0.0336 |
| **5** | LSD(P<.05) | 0.44911 | 1 |  | _ | 2 |  | _ | -0.00797 | 0.01037 | 26.94 | -0.77 | 0.4491 |

Effect=Day*Trt bygroup=4

| **Obs** | **ADJUSTMENT** | **adjp** | **Day** | **Trt** | **Period** | **_Day** | **_Trt** | **_Period** | **Estimate** | **StdErr** | **DF** | **tValue** | **Probt** |
| --- | --- | --- | --- | --- | --- | --- | --- | --- | --- | --- | --- | --- | --- |
| **6** | LSD(P<.05) | 0.52345 | 0 | Lying | _ | 0 | Sleep | _ | 0.01098 | 0.01691 | 19.72 | 0.65 | 0.5235 |
| **7** | LSD(P<.05) | 0.04423 | 0 | Lying | _ | 1 | Lying | _ | -0.02972 | 0.01396 | 22.9 | -2.13 | 0.0442 |
| **8** | LSD(P<.05) | 0.27366 | 0 | Lying | _ | 1 | Sleep | _ | 0.01916 | 0.01701 | 19.56 | 1.13 | 0.2737 |
| **9** | LSD(P<.05) | 0.00429 | 0 | Lying | _ | 2 | Lying | _ | -0.04011 | 0.01191 | 14.81 | -3.37 | 0.0043 |
| **10** | LSD(P<.05) | 0.43185 | 0 | Lying | _ | 2 | Sleep | _ | 0.01362 | 0.01698 | 19.99 | 0.80 | 0.4318 |
| **11** | LSD(P<.05) | 0.02601 | 0 | Sleep | _ | 1 | Lying | _ | -0.04070 | 0.01691 | 19.72 | -2.41 | 0.0260 |
| **12** | LSD(P<.05) | 0.56381 | 0 | Sleep | _ | 1 | Sleep | _ | 0.008177 | 0.01397 | 23.63 | 0.59 | 0.5638 |
| **13** | LSD(P<.05) | 0.00928 | 0 | Sleep | _ | 2 | Lying | _ | -0.05109 | 0.01803 | 23.61 | -2.83 | 0.0093 |
| **14** | LSD(P<.05) | 0.80880 | 0 | Sleep | _ | 2 | Sleep | _ | 0.002637 | 0.01070 | 14.3 | 0.25 | 0.8088 |
| **15** | LSD(P<.05) | 0.00954 | 1 | Lying | _ | 1 | Sleep | _ | 0.04888 | 0.01701 | 19.56 | 2.87 | 0.0095 |
| **16** | LSD(P<.05) | 0.49955 | 1 | Lying | _ | 2 | Lying | _ | -0.01039 | 0.01519 | 28.37 | -0.68 | 0.4995 |
| **17** | LSD(P<.05) | 0.01898 | 1 | Lying | _ | 2 | Sleep | _ | 0.04334 | 0.01698 | 19.99 | 2.55 | 0.0190 |
| **18** | LSD(P<.05) | 0.00330 | 1 | Sleep | _ | 2 | Lying | _ | -0.05927 | 0.01811 | 23.35 | -3.27 | 0.0033 |
| **19** | LSD(P<.05) | 0.69897 | 1 | Sleep | _ | 2 | Sleep | _ | -0.00554 | 0.01416 | 25.32 | -0.39 | 0.6990 |
| **20** | LSD(P<.05) | 0.00671 | 2 | Lying | _ | 2 | Sleep | _ | 0.05373 | 0.01811 | 24.1 | 2.97 | 0.0067 |

| The SAS System |
| --- |
| Mean separation for log protein |
| Differences of Least Squares Means |

| **Set** | **Average Sig Diff Value** | **Minimum Sig Diff Value** | **Maximum Sig Diff Value** |
| --- | --- | --- | --- |
| 1 | 0.03047 | 0.03047 | 0.03047 |
| 2 | 0.03058 | 0.03058 | 0.03058 |
| 3 | 0.01959 | 0.01707 | 0.02128 |
| 4 | 0.03273 | 0.0229 | 0.03744 |

| The SAS System |
| --- |
| Back-transformed (bt) Mean Separation for log protein |

Effect=Period Method=LSD(P<.05) Set=1

| **Obs** | **Day** | **Trt** | **Period** | **Estimate** | **Standard Error** | **Mean** | **Standard Error of Mean** | **UnTrans_Mean** | **UnTrans_Stderr** | **Letter Group** | **BT_Mean** | **BT_StdErr** |
| --- | --- | --- | --- | --- | --- | --- | --- | --- | --- | --- | --- | --- |
| **1** | _ |  | 1 | 1.0517 | 0.03659 | 1.0517 | 0.03659 | 2.8814 | 0.1050 | A | 2.86257 | 0.10473 |
| **2** | _ |  | 2 | 1.0807 | 0.03621 | 1.0807 | 0.03621 | 2.9714 | 0.1039 | A | 2.94684 | 0.10670 |

Effect=Trt Method=LSD(P<.05) Set=2

| **Obs** | **Day** | **Trt** | **Period** | **Estimate** | **Standard Error** | **Mean** | **Standard Error of Mean** | **UnTrans_Mean** | **UnTrans_Stderr** | **Letter Group** | **BT_Mean** | **BT_StdErr** |
| --- | --- | --- | --- | --- | --- | --- | --- | --- | --- | --- | --- | --- |
| **3** | _ | Lying | _ | 1.0852 | 0.03647 | 1.0852 | 0.03647 | 2.9850 | 0.1046 | A | 2.95991 | 0.10793 |
| **4** | _ | Sleep | _ | 1.0473 | 0.03636 | 1.0473 | 0.03636 | 2.8678 | 0.1043 | B | 2.84993 | 0.10363 |

Effect=Day Method=LSD(P<.05) Set=3

| **Obs** | **Day** | **Trt** | **Period** | **Estimate** | **Standard Error** | **Mean** | **Standard Error of Mean** | **UnTrans_Mean** | **UnTrans_Stderr** | **Letter Group** | **BT_Mean** | **BT_StdErr** |
| --- | --- | --- | --- | --- | --- | --- | --- | --- | --- | --- | --- | --- |
| **5** | 0 |  | _ | 1.0564 | 0.03616 | 1.0564 | 0.03616 | 2.8955 | 0.1037 | B | 2.87598 | 0.10399 |
| **6** | 1 |  | _ | 1.0672 | 0.03617 | 1.0672 | 0.03617 | 2.9266 | 0.1037 | AB | 2.90712 | 0.10515 |
| **7** | 2 |  | _ | 1.0751 | 0.03628 | 1.0751 | 0.03628 | 2.9572 | 0.1041 | A | 2.93037 | 0.10631 |

Effect=Day*Trt Method=LSD(P<.05) Set=4

| **Obs** | **Day** | **Trt** | **Period** | **Estimate** | **Standard Error** | **Mean** | **Standard Error of Mean** | **UnTrans_Mean** | **UnTrans_Stderr** | **Letter Group** | **BT_Mean** | **BT_StdErr** |
| --- | --- | --- | --- | --- | --- | --- | --- | --- | --- | --- | --- | --- |
| **8** | 0 | Lying | _ | 1.0619 | 0.03716 | 1.0619 | 0.03716 | 2.9123 | 0.1066 | B | 2.89182 | 0.10745 |
| **9** | 0 | Sleep | _ | 1.0509 | 0.03711 | 1.0509 | 0.03711 | 2.8787 | 0.1065 | B | 2.86023 | 0.10614 |
| **10** | 1 | Lying | _ | 1.0916 | 0.03716 | 1.0916 | 0.03716 | 3.0011 | 0.1066 | A | 2.97904 | 0.11069 |
| **11** | 1 | Sleep | _ | 1.0427 | 0.03716 | 1.0427 | 0.03716 | 2.8520 | 0.1066 | B | 2.83693 | 0.10541 |
| **12** | 2 | Lying | _ | 1.1020 | 0.03765 | 1.1020 | 0.03765 | 3.0417 | 0.1082 | A | 3.01016 | 0.11332 |
| **13** | 2 | Sleep | _ | 1.0483 | 0.03714 | 1.0483 | 0.03714 | 2.8727 | 0.1066 | B | 2.85269 | 0.10595 |

| The SAS System |
| --- |
| Check on normality for log protein |

The UNIVARIATE Procedure

Variable: residual (Residual (Mu scale))

| **Tests for Normality** | | | | |
| --- | --- | --- | --- | --- |
| **Test** | **Statistic** | | **p Value** | |
| **Shapiro-Wilk** | **W** | 0.953317 | **Pr < W** | 0.0180 |
| **Kolmogorov-Smirnov** | **D** | 0.087385 | **Pr > D** | >0.1500 |
| **Cramer-von Mises** | **W-Sq** | 0.099067 | **Pr > W-Sq** | 0.1159 |
| **Anderson-Darling** | **A-Sq** | 0.740308 | **Pr > A-Sq** | 0.0510 |

| **Extreme Observations** | | | |
| --- | --- | --- | --- |
| **Lowest** | | **Highest** | |
| **Value** | **Obs** | **Value** | **Obs** |
| -0.0643417 | 28 | 0.0325336 | 58 |
| -0.0635025 | 35 | 0.0340645 | 29 |
| -0.0326235 | 56 | 0.0340658 | 47 |
| -0.0296688 | 48 | 0.0545138 | 27 |
| -0.0236030 | 34 | 0.0803852 | 36 |

| **Missing Values** | | | |
| --- | --- | --- | --- |
| **Missing Value** | **Count** | **Percent Of** | |
|  |  | **All Obs** | **Missing Obs** |
| . | 7 | 10.00 | 100.00 |
