# Supplementary material for: Effects of acute lying and sleep deprivation on the behavior of lactating dairy cows
Source: PLoS One. 2019 Aug 28;14(8):e0212823. doi: 10.1371/journal.pone.0212823 (PMC6713338; doi:10.1371/journal.pone.0212823)
Supplement: S8 File — Supplemental data from SAS model to support conclusions drawn on the effects of treatment on somatic cell count from cows. (DOCX) [file pone.0212823.s010.docx]

SCC: looking at it by dat and day*trt interaction. Could not look at specific times and day interaction because not equal samples taken across baseline, trt or Recov

| The SAS System |
| --- |
| GLIMMix ANOVA for scc |

The GLIMMIX Procedure

| **Class Level Information** | | |
| --- | --- | --- |
| **Class** | **Levels** | **Values** |
| **Cow_ID** | 12 | 4444 4479 4481 4484 4486 4490 4507 4512 4518 6302 6725 13162 |
| **Day** | 3 | 0 1 2 |
| **Trt** | 2 | Lying Sleep |
| **Period** | 2 | 1 2 |

| **Number of Observations Read** | 70 |
| --- | --- |
| **Number of Observations Used** | 69 |

| Convergence criterion (GCONV=1E-8) satisfied. |
| --- |

| **Fit Statistics** | |
| --- | --- |
| **-2 Res Log Likelihood** | 113.16 |
| **AIC (smaller is better)** | 121.16 |
| **AICC (smaller is better)** | 121.86 |
| **BIC (smaller is better)** | 123.10 |
| **CAIC (smaller is better)** | 127.10 |
| **HQIC (smaller is better)** | 120.44 |
| **Generalized Chi-Square** | 6.87 |
| **Gener. Chi-Square / DF** | 0.11 |

| **Covariance Parameter Estimates** | | | |
| --- | --- | --- | --- |
| **Cov Parm** | **Subject** | **Estimate** | **Standard Error** |
| **Cow_ID** |  | 0.8688 | 0.3917 |
| **Cow_ID*Trt*Period** |  | 0.06593 | 0.04595 |
| **AR(1)** | **Cow_ID*Trt*Period** | -0.1982 | 0.2807 |
| **Residual** |  | 0.1109 | 0.02804 |

| **Type III Tests of Fixed Effects** | | | | |
| --- | --- | --- | --- | --- |
| **Effect** | **Num DF** | **Den DF** | **F Value** | **Pr > F** |
| **Period** | 1 | 11.05 | 3.75 | 0.0788 |
| **Trt** | 1 | 11.22 | 3.45 | 0.0896 |
| **Day** | 2 | 28.47 | 0.45 | 0.6445 |
| **Day*Trt** | 2 | 28.33 | 2.02 | 0.1509 |

| The SAS System |
| --- |
| Mean separation for log scc |
| Differences of Least Squares Means |

Effect=Period bygroup=1

| **Obs** | **ADJUSTMENT** | **adjp** | **Day** | **Trt** | **Period** | **_Day** | **_Trt** | **_Period** | **Estimate** | **StdErr** | **DF** | **tValue** | **Probt** |
| --- | --- | --- | --- | --- | --- | --- | --- | --- | --- | --- | --- | --- | --- |
| **1** | LSD(P<.05) | 0.078806 | _ |  | 1 | _ |  | 2 | -0.2412 | 0.1246 | 11.05 | -1.94 | 0.0788 |

Effect=Trt bygroup=2

| **Obs** | **ADJUSTMENT** | **adjp** | **Day** | **Trt** | **Period** | **_Day** | **_Trt** | **_Period** | **Estimate** | **StdErr** | **DF** | **tValue** | **Probt** |
| --- | --- | --- | --- | --- | --- | --- | --- | --- | --- | --- | --- | --- | --- |
| **2** | LSD(P<.05) | 0.089609 | _ | Lying | _ | _ | Sleep | _ | 0.2319 | 0.1248 | 11.22 | 1.86 | 0.0896 |

Effect=Day bygroup=3

| **Obs** | **ADJUSTMENT** | **adjp** | **Day** | **Trt** | **Period** | **_Day** | **_Trt** | **_Period** | **Estimate** | **StdErr** | **DF** | **tValue** | **Probt** |
| --- | --- | --- | --- | --- | --- | --- | --- | --- | --- | --- | --- | --- | --- |
| **3** | LSD(P<.05) | 0.73285 | 0 |  | _ | 1 |  | _ | -0.03732 | 0.1084 | 33.46 | -0.34 | 0.7329 |
| **4** | LSD(P<.05) | 0.53227 | 0 |  | _ | 2 |  | _ | 0.06610 | 0.1041 | 20.99 | 0.64 | 0.5323 |
| **5** | LSD(P<.05) | 0.35717 | 1 |  | _ | 2 |  | _ | 0.1034 | 0.1109 | 35.53 | 0.93 | 0.3572 |

Effect=Day*Trt bygroup=4

| **Obs** | **ADJUSTMENT** | **adjp** | **Day** | **Trt** | **Period** | **_Day** | **_Trt** | **_Period** | **Estimate** | **StdErr** | **DF** | **tValue** | **Probt** |
| --- | --- | --- | --- | --- | --- | --- | --- | --- | --- | --- | --- | --- | --- |
| **6** | LSD(P<.05) | 0.97682 | 0 | Lying | _ | 0 | Sleep | _ | -0.00514 | 0.1757 | 32.27 | -0.03 | 0.9768 |
| **7** | LSD(P<.05) | 0.21746 | 0 | Lying | _ | 1 | Lying | _ | -0.1898 | 0.1509 | 32.5 | -1.26 | 0.2175 |
| **8** | LSD(P<.05) | 0.52649 | 0 | Lying | _ | 1 | Sleep | _ | 0.1100 | 0.1717 | 30.55 | 0.64 | 0.5265 |
| **9** | LSD(P<.05) | 0.36645 | 0 | Lying | _ | 2 | Lying | _ | -0.1370 | 0.1483 | 20.43 | -0.92 | 0.3664 |
| **10** | LSD(P<.05) | 0.13608 | 0 | Lying | _ | 2 | Sleep | _ | 0.2640 | 0.1726 | 31.31 | 1.53 | 0.1361 |
| **11** | LSD(P<.05) | 0.30094 | 0 | Sleep | _ | 1 | Lying | _ | -0.1847 | 0.1757 | 32.27 | -1.05 | 0.3009 |
| **12** | LSD(P<.05) | 0.46460 | 0 | Sleep | _ | 1 | Sleep | _ | 0.1152 | 0.1557 | 34.36 | 0.74 | 0.4646 |
| **13** | LSD(P<.05) | 0.47999 | 0 | Sleep | _ | 2 | Lying | _ | -0.1318 | 0.1847 | 35.36 | -0.71 | 0.4800 |
| **14** | LSD(P<.05) | 0.08022 | 0 | Sleep | _ | 2 | Sleep | _ | 0.2692 | 0.1466 | 21.39 | 1.84 | 0.0802 |
| **15** | LSD(P<.05) | 0.09086 | 1 | Lying | _ | 1 | Sleep | _ | 0.2998 | 0.1717 | 30.55 | 1.75 | 0.0909 |
| **16** | LSD(P<.05) | 0.74414 | 1 | Lying | _ | 2 | Lying | _ | 0.05284 | 0.1607 | 35.8 | 0.33 | 0.7441 |
| **17** | LSD(P<.05) | 0.01313 | 1 | Lying | _ | 2 | Sleep | _ | 0.4539 | 0.1726 | 31.31 | 2.63 | 0.0131 |
| **18** | LSD(P<.05) | 0.18054 | 1 | Sleep | _ | 2 | Lying | _ | -0.2470 | 0.1806 | 33.57 | -1.37 | 0.1805 |
| **19** | LSD(P<.05) | 0.32292 | 1 | Sleep | _ | 2 | Sleep | _ | 0.1540 | 0.1536 | 35.17 | 1.00 | 0.3229 |
| **20** | LSD(P<.05) | 0.03436 | 2 | Lying | _ | 2 | Sleep | _ | 0.4010 | 0.1821 | 34.88 | 2.20 | 0.0344 |

| The SAS System |
| --- |
| Mean separation for log scc |
| Differences of Least Squares Means |

| **Set** | **Average Sig Diff Value** | **Minimum Sig Diff Value** | **Maximum Sig Diff Value** |
| --- | --- | --- | --- |
| 1 | 0.27399 | 0.27399 | 0.27399 |
| 2 | 0.27408 | 0.27408 | 0.27408 |
| 3 | 0.22064 | 0.21648 | 0.22495 |
| 4 | 0.34043 | 0.30447 | 0.37476 |

| The SAS System |
| --- |
| Back-transformed (bt) Mean Separation for log scc |

Effect=Period Method=LSD(P<.05) Set=1

| **Obs** | **Day** | **Trt** | **Period** | **Estimate** | **Standard Error** | **Mean** | **Standard Error of Mean** | **UnTrans_Mean** | **UnTrans_Stderr** | **Letter Group** | **BT_Mean** | **BT_StdErr** |
| --- | --- | --- | --- | --- | --- | --- | --- | --- | --- | --- | --- | --- |
| **1** | _ |  | 1 | 10.7616 | 0.2831 | 10.7616 | 0.2831 | 75096 | 23336 | A | 47175.50 | 13353.92 |
| **2** | _ |  | 2 | 11.0028 | 0.2831 | 11.0028 | 0.2831 | 96375 | 23334 | A | 60043.02 | 16998.06 |

Effect=Trt Method=LSD(P<.05) Set=2

| **Obs** | **Day** | **Trt** | **Period** | **Estimate** | **Standard Error** | **Mean** | **Standard Error of Mean** | **UnTrans_Mean** | **UnTrans_Stderr** | **Letter Group** | **BT_Mean** | **BT_StdErr** |
| --- | --- | --- | --- | --- | --- | --- | --- | --- | --- | --- | --- | --- |
| **3** | _ | Lying | _ | 10.9982 | 0.2834 | 10.9982 | 0.2834 | 90735 | 23376 | A | 59764.98 | 16935.90 |
| **4** | _ | Sleep | _ | 10.7663 | 0.2829 | 10.7663 | 0.2829 | 80736 | 23317 | A | 47394.96 | 13405.76 |

Effect=Day Method=LSD(P<.05) Set=3

| **Obs** | **Day** | **Trt** | **Period** | **Estimate** | **Standard Error** | **Mean** | **Standard Error of Mean** | **UnTrans_Mean** | **UnTrans_Stderr** | **Letter Group** | **BT_Mean** | **BT_StdErr** |
| --- | --- | --- | --- | --- | --- | --- | --- | --- | --- | --- | --- | --- |
| **5** | 0 |  | _ | 10.8918 | 0.2830 | 10.8918 | 0.2830 | 79936 | 23191 | A | 53734.85 | 15209.23 |
| **6** | 1 |  | _ | 10.9291 | 0.2824 | 10.9291 | 0.2824 | 87428 | 23115 | A | 55778.23 | 15753.88 |
| **7** | 2 |  | _ | 10.8257 | 0.2838 | 10.8257 | 0.2838 | 89843 | 23262 | A | 50297.65 | 14272.26 |

Effect=Day*Trt Method=LSD(P<.05) Set=4

| **Obs** | **Day** | **Trt** | **Period** | **Estimate** | **Standard Error** | **Mean** | **Standard Error of Mean** | **UnTrans_Mean** | **UnTrans_Stderr** | **Letter Group** | **BT_Mean** | **BT_StdErr** |
| --- | --- | --- | --- | --- | --- | --- | --- | --- | --- | --- | --- | --- |
| **8** | 0 | Lying | _ | 10.8892 | 0.2952 | 10.8892 | 0.2952 | 72694 | 24624 | AB | 53596.84 | 15820.82 |
| **9** | 0 | Sleep | _ | 10.8944 | 0.2975 | 10.8944 | 0.2975 | 87177 | 24884 | AB | 53873.21 | 16028.74 |
| **10** | 1 | Lying | _ | 11.0791 | 0.2952 | 11.0791 | 0.2952 | 93500 | 24624 | A | 64800.00 | 19127.79 |
| **11** | 1 | Sleep | _ | 10.7792 | 0.2952 | 10.7792 | 0.2952 | 81355 | 24600 | AB | 48012.51 | 14174.30 |
| **12** | 2 | Lying | _ | 11.0262 | 0.3003 | 11.0262 | 0.3003 | 106011 | 25178 | A | 61464.77 | 18456.42 |
| **13** | 2 | Sleep | _ | 10.6252 | 0.2957 | 10.6252 | 0.2957 | 73675 | 24674 | B | 41159.41 | 12171.56 |

|  |
| --- |
|  |

|  |
| --- |
|  |

| The SAS System |
| --- |
| Plot of day*trt untransformed least squares means for scc |

| The SAS System |
| --- |
| Check on normality for log scc |

The UNIVARIATE Procedure

Variable: residual (Residual (Mu scale))

| **Tests for Normality** | | | | |
| --- | --- | --- | --- | --- |
| **Test** | **Statistic** | | **p Value** | |
| **Shapiro-Wilk** | **W** | 0.970678 | **Pr < W** | 0.1043 |
| **Kolmogorov-Smirnov** | **D** | 0.132278 | **Pr > D** | <0.0100 |
| **Cramer-von Mises** | **W-Sq** | 0.132783 | **Pr > W-Sq** | 0.0412 |
| **Anderson-Darling** | **A-Sq** | 0.796304 | **Pr > A-Sq** | 0.0390 |
